# Enzyme‐Triggered l‐*α*/d‐Peptide Hydrogels as a Long‐Acting Injectable Platform for Systemic Delivery of HIV/AIDS Drugs

**DOI:** 10.1002/adhm.202203198

**Published:** 2023-03-17

**Authors:** Sophie M. Coulter, Sreekanth Pentlavalli, Lalitkumar K. Vora, Yuming An, Emily R. Cross, Ke Peng, Kate McAulay, Ralf Schweins, Ryan F. Donnelly, Helen O. McCarthy, Garry Laverty

**Affiliations:** ^1^ School of Pharmacy Queen's University Belfast Medical Biology Centre 97 Lisburn Road Belfast, Co. Antrim Northern Ireland BT9 7BL UK; ^2^ School of Chemistry University of Glasgow Joseph Black Building Glasgow Scotland G12 8QQ UK; ^3^ School of Computing, Engineering and Built Environment Glasgow Caledonian University Glasgow Scotland G4 0BA UK; ^4^ Large Scale Structures Group Institut Laue – Langevin 71 Avenue des Martyrs, CS 20156 Grenoble Cedex 9 38042 France

**Keywords:** enzyme instructed self‐assembly, HIV/AIDS, long‐acting injectables, peptide hydrogels, sustained release

## Abstract

Eradicating HIV/AIDS by 2030 is a central goal of the World Health Organization. Patient adherence to complicated dosage regimens remains a key barrier. There is a need for convenient long‐acting formulations that deliver drugs over sustained periods. This paper presents an alternative platform, an injectable in situ forming hydrogel implant to deliver a model antiretroviral drug (zidovudine [AZT]) over 28 days. The formulation is a self‐assembling ultrashort d or l‐*α* peptide hydrogelator, namely phosphorylated (naphthalene‐2‐ly)‐acetyl‐diphenylalanine‐lysine‐tyrosine‐OH (NapFFKY[p]‐OH), covalently conjugated to zidovudine via an ester linkage. Rheological analysis demonstrates phosphatase enzyme instructed self‐assembly, with hydrogels forming within minutes. Small angle neutron scattering data suggest hydrogels form narrow radius (≈2 nm), large length fibers closely fitting the flexible cylinder elliptical model. d‐Peptides are particularly promising for long‐acting delivery, displaying protease resistance for 28 days. Drug release, via hydrolysis of the ester linkage, progress under physiological conditions (37 °C, pH 7.4, H_2_O). Subcutaneous administration of Napffk(AZT)Y[p]G‐OH in Sprague Dawley rats demonstrate zidovudine blood plasma concentrations within the half maximal inhibitory concentration (IC_50_) range (30–130 ng mL^−1^) for 35 days. This work is a proof‐of‐concept for the development of a long‐acting combined injectable in situ forming peptide hydrogel implant. These products are imperative given their potential impact on society.

## Graphical abstract

This article highlights enzyme‐responsive peptide hydrogels as a long‐acting injectable platform for the systemic delivery of HIV/AIDS drugs. The versatility of the peptide motif enables the precise covalent attachment of model HIV/AIDS drug zidovudine, reducing burst release. The soluble formulation forms a hydrogel implant upon injection, in response to the presence of a phosphatase enzyme trigger, providing sustained drug release.

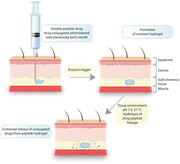

## 1 Introduction

Based on most recent statistics from 2021, there were ≈38.4 million people worldwide living with HIV/AIDS and 1.5 million new infections.^[^ ^1^ ^]^ Developing nations bear the brunt of the HIV/AIDS epidemic due to a combination of economic, environmental, and societal factors that limit the distribution and use of existing treatments.^[^ ^2^ ^]^ Traditional oral treatment with antiretroviral therapy (ART) necessitates complex multi‐drug oral regimens with a high pill burden often resulting in adherence issues. Attempts to overcome this issue have included the development of once daily pills for oral ART, however, pill fatigue is still reported.^[^ ^3^ ^]^ Adherence remains the strongest predictor in HIV prevention and viral suppression, disease progression, and death among HIV positive individuals. Indeed, adequate adherence is mandatory to reduce the risk of viral rebound, drug resistance, and treatment failure.^[^ ^4^ ^]^ Efforts to overcome adherence issues by increasing patient therapeutic choice have focused on the development of various sustained release formulations including oral, buccal, injectable and implantable technologies, with injectables receiving particular interest.^[^ ^5^ ^]^

The recent approval of new long‐acting injectable antiretroviral formulations, for example, GSK/Janssen's injectable Cabenuva (cabotegravir and rilpivirine),^[^ ^6^ ^]^ demonstrates the promise of long‐acting injectables in HIV/AIDS and throughout healthcare. However, one limitation of injectable technologies is that they are generally formulated as a suspension of hydrophobic drug. While suspensions often enable high drug loading they are fundamentally unstable, requiring formulation strategies to ensure that physical stability and drug particle size are maintained over the product shelf‐life.^[^ ^7^ ^]^ An increase in drug particle size over time, attributed to Ostwald Ripening, can lead to syringe clogging and incomplete dose administration, necessitating the employment of larger needles (16G) which are associated with increased pain upon injection and reduced patient acceptability. Ostwald Ripening accelerates under fluctuating temperatures, and this is a major concern for HIV/AIDS formulations, which are particularly required in developing regions such as sub‐Saharan Africa.^[^ ^8^ ^]^ These areas tend to experience extreme differences in climates and possess limited infrastructure required for reliable storage of such medicines, for example cold‐chain supply.

The use of pre‐formed implants comprising non‐biodegradable polymers, for example those employed in the contraceptive Nexplanon, would require costly and painful surgical procedures for insertion and removal. This approach can also produce a relatively high initial drug concentration in the systemic compartment, the so‐called burst effect, with drug concentrations declining steadily thereafter. This makes it difficult to optimize drug dosing and pharmacokinetics.

Recent efforts to provide a convenient oral formulation for HIV/AIDS prevention have focused on pre‐exposure prophylaxis (PrEP) with various formulations in the clinical pipeline. Currently approved PrEP consists of a two‐drug regimen, a once daily oral tablet containing tenofovir disoproxil fumarate and emtricitabine (Truvada)^[^ ^9^ ^]^ or a once daily oral tablet containing tenofovir alafenamide and emtricitabine (Descovy).^[^ ^10^ ^]^ PrEP's efficacy in preventing HIV/AIDS varies according to the degree of adherence, which is therefore key in determining its effectiveness.^[^ ^11^ ^]^ The use of oral PrEP for the prevention of HIV‐1 transmission has been widely favorable with compliant use thought to reduce transmission rates by ≈90%.^[^ ^12^ ^]^ However, strict adherence to dosage regimens is crucial for the maintenance of protective plasma and tissue concentration of drugs.^[^ ^13^ ^]^ This can be more difficult to achieve in developing countries. Research also shows that most patients prefer monthly injections to vaginal rings or daily tablets, therefore justifying a possible focus on developing injectable implants and the importance of wider product choice to patients.^[^ ^14^ ^]^

**Scheme 1:**
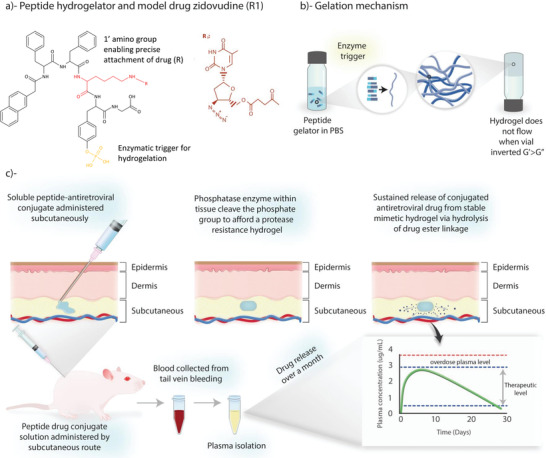
Concept for using peptide hydrogelators as an enzyme‐triggered, long‐acting injectable delivery platform for HIV/AIDS. a) Chemical structure of L‐*α* peptide hydrogelator NapFFKY(p)G‐OH and covalently conjugated antiretroviral drug (R1). R1 depicts the model antiretroviral zidovudine attached to peptide sequence via ester linkage. b) Assembly of peptide gelators upon exposure to an enzyme trigger resulting in the formation of a 3D network of entangled fibers which entrap water and form a hydrogel. Propensity to gelate is initially screened by the absence of flow upon vial inversion and confirmed via oscillatory rheology. Adapted from work by Adams and by Zhou.^[^ ^19^ ^]^ c) Demonstrates the intended clinical application for this platform. Soluble peptide‐antiretroviral conjugate is administered monthly via subcutaneous injection; phosphatase enzymes within the tissue cleave the phosphate group from the peptide sequence backbone, forming a protease resistant hydrogel. Sustained release of the conjugated antiretroviral drug from stable peptide hydrogel then occurs via hydrolysis of drug‐ester linkage. In vivo studies demonstrate zidovudine drug release within therapeutic levels over the dosage interval.

**Figure 1:**
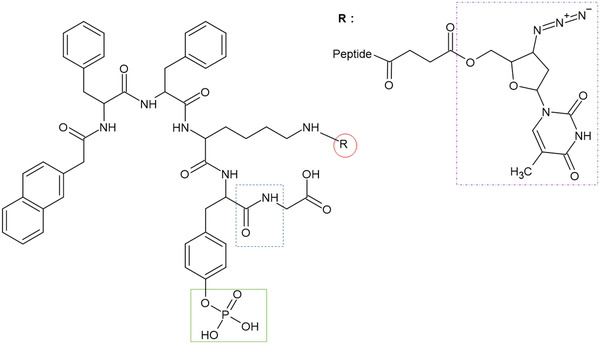
Chemical structure of peptide hydrogelator NapFFKY(p)G‐OH and covalently conjugated antiretroviral drug (R). R depicts the model antiretroviral zidovudine (shown in purple dash dot box) attached to peptide sequence via ester linkage. Our peptide molecules have three highly desirable functionalities: i) a peptide sequence allowing hydrogel formation; ii) a phosphate group (green square) endowing solubility, which upon removal acts as an enzymatic (phosphatase) trigger for hydrogelation; and iii) a single primary amino group (—NH_2_) allowing drugs to be covalently attached with precision (red circle) via an ester linkage prone to hydrolysis in the physiological environment. Peptides studied include L‐*α* NapFFKY(p)‐OH and D enantiomer form (NapffkY(p)‐OH) with and without glycine (blue dashed line) attached at carboxylic acid peptide terminus. The complete library of structures studied is outlined in Figure S2, Supporting Information.

This paper aims to overcome some of the limitations of existing formulations and to present a novel alternative platform for drug delivery by covalent attachment of drugs to ultrashort peptide motifs. Our approach creates a fully soluble formulation with the ability to form hydrogels in response to phosphatase enzymes present in the skin space (**Scheme** **1**). This enables formation of an in situ forming drug delivery system that exists as a soluble product upon injectable administration, forming a hydrogel implant within the subcutaneous or intramuscular injection site. The unique chemical and functional versatility of peptide molecules have allowed them to be explored for use as drug delivery carriers for poorly soluble drugs. One way to increase the solubility of a drug is to attach it directly to a water‐soluble polymer.^[^ ^15^ ^]^ Peptide‐based hydrogels are more amenable to manipulation at the molecular scale relative to synthetic polymers, offering the ability to tune features such as drug release, viscoelastic and mechanical properties.^[^ ^16^ ^]^ Our hydrogel forming peptides possess a single drug conjugatable chemical functional group, enabling precise drug attachment via a labile ester linkage, and improving drug solubility. In practice this should enable greater drug homogeneity and loading and allow tunable drug release rates from the peptide‐based hydrogel, for example, stimuli–responsive hydrogel formation and drug release by hydrolysis under physiological conditions.^[^ ^17^ ^]^ Additionally, attachment via labile ester linkage enables drug to be released in an unmodified form (see **Figure** **1**). Unlike alternative stimuli–responsive in situ systems—which unhelpfully rely upon organic solvent exchange, photo‐initiation, thermo‐responsive polymers, salt screening or pH‐induced gelation—our system makes use of endogenous enzymes as a safe, rapid, and convenient mechanism for triggering implant formation.^[^ ^18^ ^]^

Peptide hydrogels are an emerging class of materials for use within a variety of applications including: optoelectronics,^[^ ^20^ ^]^ 3D printing,^[^ ^21^ ^]^ 3D cell culture,^[^ ^22^ ^]^ antimicrobial,^[^ ^23^ ^]^ and cancer therapy.^[^ ^24^ ^]^ The Xu group has previously studied the local delivery of HIV/AIDS antiretrovirals using a range of ultrashort phosphorylated non‐steroidal anti‐inflammatory (NSAID) and (naphthalene‐2‐ly)‐acetyl‐diphenylalanine‐lysine‐tyrosine‐OH based peptides for administration as vaginal microbicides with prostate acid phosphatase, derived from sperm, as the local enzyme trigger for hydrogel formation.^[^ ^17a^ ^]^ The use of such stimuli–responsive self‐assembling peptide systems as long‐acting in situ sustained release implants for subcutaneous or intramuscular administration, to our knowledge, has not been previously investigated.

This work presents an important proof‐of‐concept study with our ultimate goal being future clinical translation after conducting comprehensive animal/human safety and efficacy studies. We envisage our formulation to be administered as a soluble injection of low viscosity, enabling use of narrow bore needles and overcoming stability issues (e.g., precipitation) observed with current suspension‐based formulations. The platform is amenable to modification at the molecular scale and the attachment of various antiretrovirals would enable the development of a multi‐drug formulation as a convenient and effective long‐acting formulation. Our formulation has the potential to be administered in a solubilized form due to the presence of a hydrophilic phosphate group (green) attached to the peptide motif (Figure 1). Endogenous phosphatases within the hypodermis or intramuscular space remove this phosphate grouping, reducing the molecule's solubility, and resulting in hydrogel formation. Following administration, we aim for the hydrogel to form rapidly in response to phosphatase enzymes with fast sol‐to‐gel kinetics to limit initial burst release. We hypothesize drug release to be partly controlled by hydrolysis of this ester bond, thereby reducing burst release of a drug. Sustained release of unmodified drug from the hydrogel will proceed within the physiological environment, providing long‐term therapeutic cover (e.g., for at least 28 days) and removing the need for daily repeated drug doses. L‐*α* peptides form the building blocks of tissue, therefore their use as drug releasing implants is promising but limited by rapid breakdown in vivo (within hours). Our aim is to improve stability by including D‐peptide amino acid enantiomers within the peptide design. This should retain the beneficial properties of L‐*α* peptides (e.g., easy drug attachment) but provide superior resistance to peptide‐targeting protease enzymes.

## 2 Results and Discussion

The methods employed in this study are important in the development of our long‐acting injectable product for HIV/AIDS and serve as an important proof‐of‐concept. In practice, we would envisage our product to be a solid freeze‐dried powder formulation that will be transported to a clinic for administration to patients by a trained healthcare professional. This would be dissolved in sterile water for injection or a suitable buffer immediately prior to subcutaneous or intramuscular injection. This would reduce the effects of temperature fluctuations upon transport and/or storage, which may be especially problematic due to lack of suitable temperature‐controlled infrastructure in areas of the developing world, for example, sub‐Saharan Africa. While products such as this are often expensive to manufacture and incur additional costs when administered by trained healthcare professionals, it is important to consider the cost of treatment holistically. Effective adherence to treatment regimens will likely reduce overall healthcare costs in the longer term.

### 2.1 Peptide Synthesis, Drug Conjugation, Purification, Identification, and Formulation

All l‐*α* and d‐peptides and their corresponding zidovudine (AZT) conjugates were successfully synthesized, purified to within pharmacopoeial limits (≥95%) and identified using ^1^H NMR and mass spectrometry.^[^ ^25^ ^]^ Zidovudine was selected as the model antiretroviral for this proof‐of‐concept study due to its widespread use in HIV/AIDS drug delivery research and its long‐established clinical profile. In clinical practice a more potent antiretroviral may be preferred to achieve clinically relevant concentrations for ≥28 days. Zidovudine is a nucleoside reverse transcriptase inhibitor (NRTI) that halts proviral DNA synthesis through the inhibition of reverse transcriptase. It was the first approved therapy, and its properties are therefore well documented within the literature. There is an abundance of comparator data, for example, readily available IC_50_ values and several other formulations have been tested in animal models making it a good initial candidate for preliminary study with our system.^[^ ^26^ ^]^ Zidovudine is a low molecular weight drug. This is beneficial in ensuring that once attached covalently via a peptide‐drug linker, it should not impede noncovalent interactions that enable these ultrashort peptides to self‐assemble into supramolecular hydrogels.^[^ ^27^ ^]^ Ultimately this is a proof‐of‐concept study and future studies will include more potent antiretroviral drugs.

### 2.2 Gelation Propensity and Mechanical Characterization

**Figure 2:**
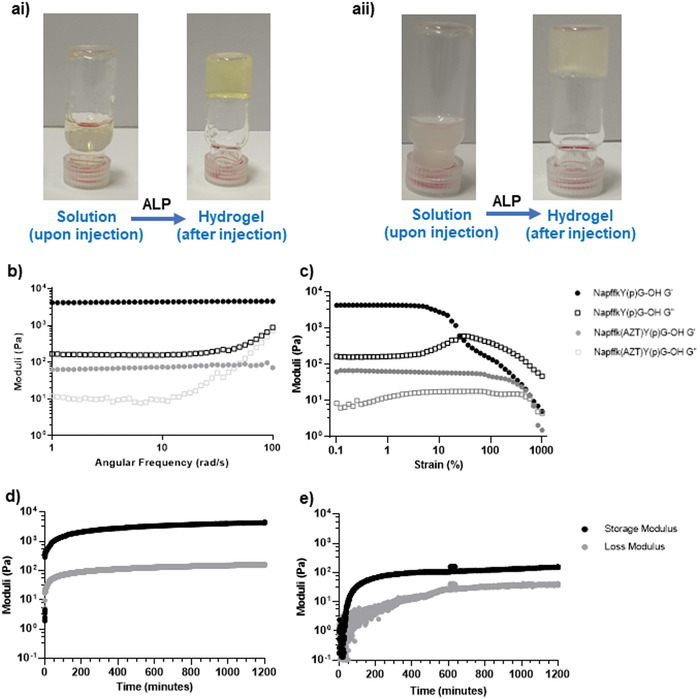
a) Gel inversion assay for the parent compound a‐i) 2 w/v% NapffkYG‐OH and the drug conjugate a‐ii) 2 w/v% Napffk(AZT)YG‐OH. Phosphorylated peptide exists as a solution prior to addition of 2 U alkaline phosphatase enzyme (ALP), which results in self‐assembly. b–e) A selection of important rheology data relating to NapffkYG‐OH and Napffk(AZT)YG‐OH. b) Frequency sweeps measured using an Anton Paar 302 rheometer with a vane and cup geometry on 2 w/v% formulated peptide gels. c) Strain sweeps measured using an Anton Paar 302 rheometer with a vane and cup geometry on 2 w/v% formulated peptide gels. In (b) and (c) the parent compound (NapffkYG‐OH) is presented in black and the zidovudine conjugate (Napffk(AZT)YG‐OH) is presented in gray. Filled circles represent G′, open squares represent G″. (d) and (e) time sweeps measured using an Anton Paar 302 rheometer with a sand blasted parallel plate PP50/S system (37 °C, 0.5 mm measuring gap) on d) 2 w/v% NapffkYG‐OH and e) 2 w/v% Napffk(AZT)YG‐OH. In (d) and (e) black lines represent G′, gray lines represent G″. For full rheological analysis see S10–S12, Supporting Information.

Our technology harnesses the use of endogenous phosphatase enzymes to achieve gelation. The normal serum range of alkaline phosphatase ranges between 20–140 U L^−1^.^[^ ^28^ ^]^ Various concentrations of alkaline phosphatase have been presented throughout the literature to afford enzyme responsive gelation of peptide precursors containing phosphate groups. Yang et al. reported the addition of 1 µL of a 10–30 U µL^−1^ solution of alkaline phosphatase to afford gelation of Fmoc‐Y(p) at a concentration of 1.9 w/v%.^[^ ^29^ ^]^ Li and colleagues reported the addition of alkaline phosphatase at a precursor concentration of 1 U mL^−1^.^[^ ^17a^ ^]^ Ling et al. reported the addition of 0.1 mL of an alkaline phosphatase solution of 38 U mL^−1^ to achieve gelation of the precursor NapFFK(taxol)C(doxorubicin)Y(p) at a concentration of 1.0 w/v%.^[^ ^17b^ ^]^ Wang and colleagues reported the successful coassembly of Nap‐GFFY(p)‐OMe and Nap‐GffY(p)‐OMe and ovalbumin by alkaline phosphatase catalysis in vitro at a concentration of 2 U/100 µL of precursor.^[^ ^30^ ^]^ The group went on to demonstrate successful gelation in vivo in mice despite the high concentration of alkaline phosphatase used to elicit gelation in initial in vitro experimentation. A concentration of 1000 U mL^−1^ with 2 µL added to each gelator (equivalent to 2 U) was chosen for experimentation since it is within the range of concentrations of alkaline phosphatase previously reported to afford gelation, as discussed above. A small volume is necessary to avoid significantly changing the concentration of the peptide precursor. Successful gelation was observed in in vivo experiments (see Figure S27, Supporting Information). Administration as a solubilized formulation, followed quickly by in situ gelation and implant formation is a preferred approach to reduce burst release of “free solubilized” drug and enable the use of narrow higher gauge needles (e.g., 28 G). These are associated with reduced patient discomfort and therefore higher acceptability.^[^ ^31^ ^]^ The development of a solubilized aqueous formulation is also important within the context of HIV/AIDS as it reduces the potential for aggregation, as observed in suspension‐based systems, when hydrophobic drug combinations are incorporated within one product.^[^ ^32^ ^]^ Multiple drugs must be administered clinically to reduce the development of antiviral resistance. The incorporation of multiple drugs within our peptide forms a key part of our ongoing research. At the highest concentrations employed (2 w/v%) some of the hydrogels appear opaque (**Figure** **2**a and Figure S7, Supporting Information). This is common for low molecular weight peptide gelators and is likely due to nanofibers overlapping to form large domains within the hydrogel network, causing scattering of light.^[^ ^33^ ^]^ Propensity to gelate indicates successful removal of the phosphate grouping upon the addition of alkaline phosphatase, thereby reducing the solubility and enabling gelation to occur. The successful removal of the phosphate grouping over time was confirmed via ^31^P NMR (Figure S7, Supporting Information). The ability of each peptide and peptide‐drug conjugate to gelate was initially screened using a vial inversion assay to assess critical gelation concentration (w/v%). These are outlined in Table S1, Supporting Information. The addition of glycine as a spacer to both l‐*α* and d peptide variants reduced the critical gelation concentration from 1.5 w/v% to 0.5 w/v%. Glycine has been employed previously as a simple hydrophilic head group to improve the strength of assembled peptide hydrogels. Therefore, its inclusion into the overall ultrashort peptide motif warrants study to improve the propensity for the formulation to gelate.^[^ ^34^ ^]^ The inclusion of drug (zidovudine) or a switch from L‐*α* to D enantiomer appeared to have no impact on the “propensity to gelate” based on comparisons of critical gelation concentrations (Table S1, Supporting Information).

Oscillatory rheology is a powerful analytical tool to understand the impact of primary structure modifications on hydrogel formation and mechanical properties.^[^ ^35^ ^]^ As shown in Figure 2b–e and Figures S13–S15, Supporting Information, our 2 w/v% hydrogels are soft in nature (G′ of ≈1000–10 000 Pa), typical of low molecular weight peptide gelators and consistent within the range observed previously by the Xu group for zidovudine gels.^[^ ^17a^ ^]^ Covalent drug attachment lowers gel viscosity in all peptides (l‐*α*, d, and presence/absence of a glycine spacer) as there is an observable decrease in G′ obtained from frequency sweeps from ≈1000 Pa range for parent peptide compound to ≈100 Pa for zidovudine conjugates studied at 2 w/v% (Figure 2b and Figure S13, Supporting Information). The conjugation of a drug to a parent peptide can interfere with intra‐ and intermolecular bonding, affecting the hydrogel's properties. For example, Chen et al. reported differences in the molecular packing of a tetrapeptide hydrogelator conjugated to the anti‐inflammatory drug ketoprofen.^[^ ^36^ ^]^ They documented that the two hydrophobic phenyl groups, conferred by ketoprofen in the predominantly hydrophilic plane of the peptide sequence, can disrupt molecular packing. This reduces lateral associations between peptides and results in a narrower filament‐like appearance compared to the parent valine‐aspartic acid (VEVE) repeating peptide sequence. No difference is observed in G′ when comparing frequency sweeps between L‐*α* and D‐enantiomer peptide gels or in the presence and absence of a glycine spacer.

Interestingly gel strength for all peptides, measured by breakage strain/flow point (strain sweeps, Figure 2c and Figure S11, Supporting Information), was improved by the attachment of zidovudine. For example, the parent peptide NapffkY‐OH breaks under a strain of ≈30% but Napffk(AZT)Y‐OH requires a strain of ≈400% to break (Figure S15c, Supporting Information). Napffk(AZT)YG‐OH demonstrated the highest gel strength, resistant to breakdown to a strain of 836% while its parent peptide broke under a strain of 30.4% (Figure 2c). The high breakage strain of zidovudine‐attached peptides suggests that their fibrous hydrogel network may be composed of different types of entanglements. For the parent peptide alone (no zidovudine attached), gel stiffness is not affected by switching between L‐*α* and D‐enantiomers with all G′ values ≈10^3^ Pa. A decrease in gel stiffness is, however, observed upon conjugation of zidovudine. Time sweeps were performed to determine the time for gelation in response to alkaline phosphatase enzyme. This is particularly important for in situ forming drug releasing implants as rapid gelation should limit the accessibility of water to the peptide‐drug ester linkage upon administration, therefore limiting the degree of burst release by providing an additional gel network/diffusional barrier.^[^ ^37^ ^]^

The most rapidly forming hydrogels were variants of NapffkY‐OH. A rheological time sweep measured the gelation time to occur after 10 s (Figure S16e and Table S3, Supporting Information), that is, the point at which the G′ and G″ values cross indicating more solid‐like behavior.^[^ ^38^ ^]^ The G′ value becomes one order of magnitude greater than the G″ value after 20 s, stabilizing in the kilopascal range after ≈70 s. These values indicate that it takes time for the gel strength to increase owing to the gradual removal of phosphate groups on the peptide structure. The addition of zidovudine, forming Napffk(AZT)Y‐OH hydrogels, extends gelation time to 50 s, with G′ > 2 × G″ at 5.83 min. This trend of increased gelation time after drug conjugation is observed for all other peptide gelators as demonstrated in Figure 2b–e (full data in Figure S16, Supporting Information). The removal of the phosphate grouping requires longer than 28 min for all peptide variants other than NapffkY(p)‐OH and Napffk(AZT)Y(p)‐OH. This indicates that complete removal of all phosphate groupings is not required to achieve gelation but rather the gel strength increases over time with continued removal of phosphate groupings.

Rheological assessment demonstrates three general trends: L‐*α* sequences and their D‐form counterparts display similar stiffness (similar G′ values), the covalent conjugation of zidovudine decreases the gel stiffness in all cases and the inclusion of zidovudine results in an increase in the mechanical strength of the gel but with extended gelation times.

### 2.3 Microscopy and Spectroscopy

**Figure 3:**
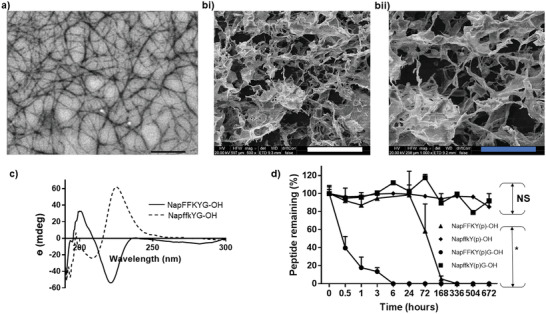
a) TEM image showing fibrous architecture of 2 w/v% Napffk(AZT)YG‐OH hydrogel at 20 000× magnification, black scale bar = 500 nm. b) SEM images (HV = 20 kV) showing fibrous architecture of 2 w/v% Napffk(AZT)YG‐OH hydrogels at i) 500× magnification and ii) 1000× magnification, white scale bar = 200 µm, blue scale bar = 100 µm. c) CD spectra for 2 w/v% NapFFKYG‐OH (solid line) and NapffkYG‐OH (dashed line) hydrogels. d) The biostability of the hydrogelators NapFFKY(p)‐OH (black triangle), NapffkY(p)‐OH (black diamond), NapFFKY(p)G‐OH (black circle) and NapffkY(p)G‐OH (black square) after incubation with the broad‐spectrum protease, proteinase K, over 28 days (672 h = 28 days). Values represent mean ± SD (*n* = 3). Key: NS: no significant (*p* > 0.05), *p ≤ 0.05 difference between the proteinase K treated peptide and the negative, non‐treated, peptide only control.

Microscopic investigation via TEM and SEM revealed a 3D random entanglement of fibers, as shown in **Figure** **3**a,b, and Figures S8–S11, Supporting Information, consistent with other low molecular weight peptide gelators studied by our group.^[^ ^23^, ^39^ ^]^ The purpose of CD spectroscopy was to confirm the chirality of the sequences, to identify differences between the L‐*α* and D‐forms, and also to monitor the self‐assembled system to provide an estimation of likely secondary structure. Experiments were performed at gelation concentrations (2 w/v%) in order to minimize light scattering and other artefacts.^[^ ^40^ ^]^ Peptides were also tested at 0.5 w/v% to determine any concentration dependent differences in secondary structure (Figure 3c and Figure S12, Supporting Information). A short path length cuvette (0.01 mm) was employed to negate the need for dilution of samples to avoid detector saturation in CD spectroscopy which may alter concentration dependent assembly.^[^ ^41^ ^]^ L‐*α* form hydrogels displayed two key peaks, a positive signal at ≈195–200 nm due to the *π* → π* transition and a negative signal at ≈220 nm owing to the *n* → π* transition. These results are consistent with *β*‐sheet formation. In the case of D‐form hydrogels these signals were also observed but the peaks were inverted, consistent with a change in the conformation as a result of chirality. Since the principle of CD spectroscopy relies on the rotation of plane polarized light and optical isomers rotate plane polarized light in opposite directions, it is consistent that the spectra obtained for L‐*α* and D‐isomers should be inverted, that is, they should rotate light to the same degree in opposite directions as observed by Scolnik and colleagues.^[^ ^42^ ^]^ The slight shift, that is, the spectra are not completely inverted, is likely due to the presence of the L‐*α* form of tyrosine in both sequences. The primary structure of each of the peptide sequences synthesized conforms to the requirements for *β*‐sheet formation, namely, polarity conferred by side chain groups (lysine), capacity to participate in hydrogen bonding and the presence of non‐polar, hydrophobic side chains (phenylalanine, 2‐naphthanoyl).^[^ ^43^ ^]^ The presence of glycine within the sequence enabled spectra to be obtained at 0.5 w/v%, consistent with the critical gelation concentration obtained for these sequences, with higher concentrations displaying increased *β*‐sheet density. When zidovudine is conjugated to each of the sequences (Figure S13b,d, Supporting Information) a similar pattern is observed, however the overall ellipticity is reduced which suggests a weaker *β*‐sheet network.

### 2.4 Small Angle Neutron Scattering (SANS)

Peptides form gels that are proven to effectively scatter neutrons and the data obtained can be used to determine the underlying structure of fibrous hydrogel networks.^[^ ^20^, ^44^ ^]^ Figure S17, Supporting Information, demonstrates that 2 w/v% peptide hydrogels (dotted line) for L‐*α*, D‐enantiomer, and zidovudine attached variants in a closely fit model data for the flexible cylinder elliptical model with the Power Law applied (straight line). The fiber radius of each is: 1.495, 2.038, 1.939 and 1.959 nm, respectively, for non‐zidovudine attached peptides (NapFFKY‐OH, NapFFKYG‐OH, NapffkY‐OH, and NapffkYG‐OH) and 1.998, 2.279, 2.261 and 2.152 nm for zidovudine conjugated variants (NapFFK(AZT)Y‐OH, NapFFK(AZT)YG‐OH, Napffk(AZT)Y‐OH and Napffk(AZT)YG‐OH), respectively. These observations are consistent with previous studies on dipeptide NapFF peptide hydrogels formed by a glucono‐*δ*‐lactone (GdL) pH triggered approach.^[^ ^45^ ^]^ SANS data also showed that the composition of the gel fibers is similar at low Q. Therefore, the differences in gel stiffness when the drug is attached are likely due to entanglement of fibers, rather than the composition of fibers themselves or their secondary structures. The lengths of these fibers are also very large (Table S4, Supporting Information), which is also a common property of entangled gel fibers. The presence of entangled gel fibers also suggests that there is a large component of gel stiffness/strength that can be controlled by external conditions, for example the gelation process. This may allow a change in gelation or formulation parameters to optimize material specifications, most notably gel strength and therefore drug release kinetics, for long‐acting drug delivery. It has been previously demonstrated that varying the peptide gel formulation method, for example enzyme, pH, temperature or salt (calcium) triggering, can be effective in altering the structure of fibrous hydrogel networks and their mechanical properties.^[^ ^46^ ^]^

### 2.5 Biostability

Proteinase K exhibits broad‐spectrum activity against aliphatic and aromatic peptide‐based substrates, providing an in vitro indication of peptide biostability.^[^ ^47^ ^]^ It is well established that L‐*α* peptides, including hydrogel forms, degrade within hours/days of exposure to proteases and are a major limitation for their clinical use.^[^ ^48^ ^]^ The data (Figure 3d) show that our d‐peptides possess superior stability relative to L‐*α* forms. After 672 h (28 days), 91.9% of NapffkY(p)G‐OH and 85.4% of NapffkY(p)‐OH remained, while their L‐*α* form counterparts were entirely degraded. This highlights the limitations of the use of L‐*α* peptides alone as long‐acting drug delivery implants since they are prone to rapid proteolytic degradation. It is important to ensure stability for the duration of drug release and the administered dosage interval, that is, 28 days. Our data indicated this could be achieved by focusing on D‐enantiomer peptide‐mimetics. L‐*α* peptide modification could tailor peptide‐mimetics toward the desired degradation profile at the end of the respective dosage interval when the drug reservoir has been exhausted and the next dose is to be administered. Concerns have been raised as to the fate of d‐peptides in vivo and whether they will accumulate due to enhanced biostability. However, evidence suggests that d‐peptides do in fact degrade in vivo but at significantly reduced rates.^[^ ^49^ ^]^ Contrary to previous thought, enzymes that target d‐peptides are present in mammals,^[^ ^50^ ^]^ for example, D‐amino‐acid oxidase which targets several neutral (e.g., phenylalanine) and basic (e.g., lysine) D‐amino acids within our D‐peptide. Our study suggests that sequences are unlikely to be degraded entirely after the desired 28 day interval but we predict they should degrade within months of administration, after the drug reservoir has emptied, with injection site rotation necessary. Tailoring of degradation profiles to drug release/dosage intervals and clinical pharmacokinetic profiles will be important considerations in future studies.

### 2.6 Cell Cytotoxicity and Biocompatibility

**Figure 4:**
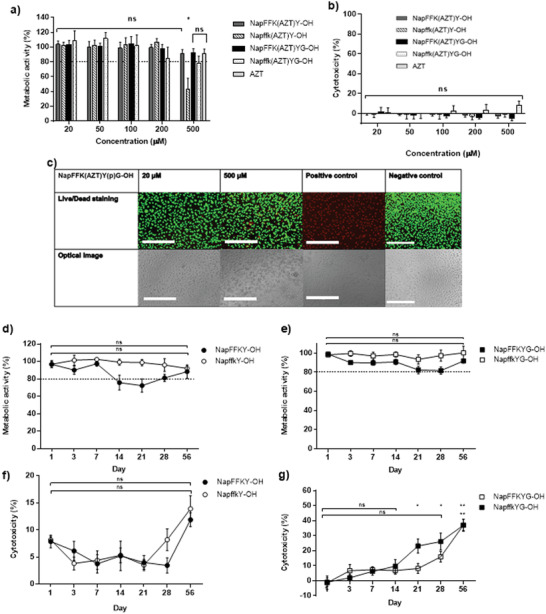
Cell cytotoxicity of fully solubilized zidovudine conjugated peptides using a) MTS viability assay (24 h), b) LDH toxicity assay (6 h), and c) Live/Dead staining (NapFFK(AZT)Y(p)G‐OH: 24 h, scale bar: 400 µm). Means ± SD provided for nine replicates in (a) and (b). Key: NapFFK(AZT)Y(p)‐OH: dark gray, Napffk(AZT)Y(p)‐OH: dashed, NapFFK(AZT)Y(p)G‐OH: black, Napffk(AZT)Y(p)G‐OH: dotted, 500 µm zidovudine drug only (light gray). ns: no significant (*p* > 0.05), *p ≤ 0.05, **p < 0.01, ***p < 0.001, ****p < 0.0001 difference between the peptides and the negative control (media only). Cell cytotoxicity of 2 w/v% peptide hydrogels using d,e) 56 day indirect toxicity using MTS viability assay and f,g) 56 day indirect toxicity using LDH toxicity assay. Means ± SD provided for three replicates. Key: NapFFKY‐OH: black circles, NapffkY‐OH: unfilled circles, NapFFKYG‐OH: black squares: NapffkYG‐OH: unfilled squares. ns: no significant (*p* ≥ 0.05), *: *p* < 0.05, **: *p* < 0.01, ***: *p* < 0.001, ****: *p* < 0.0001 difference between peptide treated cells and the negative control (media only).

A combination of MTS viability, LDH, Live/Dead staining, and hemolysis assays provided insight into the biocompatibility and cell cytotoxic profile of our peptide and peptide‐drug platforms. Short‐term studies (up to 72 h), utilizing fully solubilized formulations and MTS, demonstrated no significant cytotoxicity across all peptides tested below 500 µm (24 h: **Figure** **4**a, and Figures S17 and S18, Supporting Information). These were all within the 70–80% cell viability requirement laid down by ISO standards for drug and biomaterial testing.^[^ ^51^ ^]^ A reduction in metabolic activity was observed with increasing treatment time and was significant at 500 µm concentrations for all zidovudine conjugated peptides and the zidovudine control at 72 h (Figure S19, Supporting Information). Interestingly this was not observed for non‐zidovudine containing peptides, suggesting a possible link between the presence of zidovudine and toxicity after 72 h at 500 µm. This closely correlates with previous studies for NapFF peptide motifs, and the concentration range reflects the possible diffusion of solubilized peptide from the hydrogel implant.^[^ ^17^, ^52^ ^]^ Only 500 µm zidovudine‐conjugated D‐enantiomer Napffk(AZT)Y(p)‐OH demonstrated a significant reduction in cell metabolic activity (40–51%) at each timepoint using MTS (Figure 4a and Figure S18, Supporting Information). The reason for this observation is unclear, given that neither NapffkY(p)‐OH nor NapffkY(p)G‐OH demonstrate similar toxicity. There are several reports regarding differences in cytotoxicity between peptide enantiomers. However, these are primarily observed in vivo, where it is assumed that the D‐form's resistance to proteolytic degradation provides elevated toxicity risk due to increased bioavailability and retention.^[^ ^53^ ^]^ Yuan et al. found the D‐form of a hydrogelator composed of a nucleobase, amino acid and saccharide, to be less cytotoxic than the L‐enantiomer at a concentration of 500 µm.^[^ ^54^ ^]^ Conversely, Hyland et al. found that the D‐form of oligopeptides resulted in reduced cell proliferation and reduced cell viability when compared to the L‐*α* form.^[^ ^55^ ^]^ Luo and colleagues demonstrated no significant difference between the D‐ and L‐*α* forms of a self‐assembling peptide, EAK16, when used as a scaffold for 3D cell culture.^[^ ^56^ ^]^ It is therefore important to assess chirality associated biocompatibility and cytotoxicity differences on a case‐by‐case basis, and further analysis, particularly in vivo, is required to assess the overall toxicity of Napffk(AZT)Y(p)‐OH. LDH cytotoxicity (Figure 4b and Figure S19, Supporting Information) and Live/Dead staining (Figure 4c and Figures S20–S22, Supporting Information) did not demonstrate significant toxicity for any peptide/peptide‐drugs at the 6 and 24 h timepoints studied. Live/Dead imaging provides qualitative data regarding the health of the cells and the number of live and dead cells present was quantified (Figures S21 and S22, Supporting Information). In all cases, the fluorescence images demonstrate a predominance of live cells compared to dead cells after 24 h of treatment. The number of live cells remained above 80% at the highest concentration (500 µm), which is in line with the upper threshold for biocompatibility according to the ISO standards.^[^ ^51^ ^]^

Hemolysis is often studied as a screening tool for antimicrobial peptide toxicity and was utilized here to study the cell membrane toxicity of solubilized peptides (Figure S24, Supporting Information).^[^ ^57^ ^]^ The percentage hemolysis was observed to rise with increasing peptide concentration. However, hemolysis remained non‐significant up to concentrations of 500 µm. However, there are concerns with the reliability of hemolysis assays as a primary indicator of toxicity and it has been shown to be affected by the osmotic strength of samples.^[^ ^58^ ^]^ Bucki and colleagues demonstrated that the antimicrobial peptide LL‐37 did not damage erythrocytes in isotonic solutions but could be rendered hemolytic under hypo‐osmotic conditions.^[^ ^59^ ^]^ The main limitation of such an assay is therefore osmotic variations in the test media resulting in over‐estimation of toxicity.^[^ ^39b^ ^]^ However, it remains a useful tool when results are interpreted alongside MTS viability, LDH cytotoxicity, and Live/Dead assays.

Indirect toxicity studies were performed using 2 w/v% hydrogel forms of each of the peptides using both MTS (Figure 4d,e) and LDH (Figure 4f,g) to provide an indication of longer‐term toxicity of peptide implants. Peptides retained their hydrogel structure for the assay duration (Figure S25, Supporting Information). For all peptides, the MTS metabolic activity remained above 70% throughout the 56 day study (Figure 4d,e). However, data for the NapFFKYG‐OH hydrogel within the LDH assay (Figure 4f,g) demonstrated significant cell toxicity after 21 days (23.2%) rising to 37% after 56 days. NapffkYG‐OH showed a similar level of toxicity (37.1%) after 56 days but its toxicity was non‐significant at earlier timepoints. The metabolic activity remained higher in indirect studies at concentrations of 2 w/v%, where the cells were exposed to mainly peptide extracts, than in direct studies where peptides were present at concentrations of up to 500 µm in dissolved form. In vitro data should be considered primarily as an indicator for potential in vivo toxicity and cannot be conclusively used to determine clinical suitability. Indeed, the data presented is based on a single cell monolayer, which is considered much more susceptible to toxic effects than that of a multi‐layered tissue or organ system. Human studies would be necessary to provide the ability to study, pharmacokinetics, biodistribution, and bioaccumulation pathways.^[^ ^60^ ^]^ Further in vivo studies, initially in small mammals, are necessary to evaluate toxicity and biocompatibility (see Section 2.8).

### 2.7 In Vitro Drug Release

**Figure 5:**
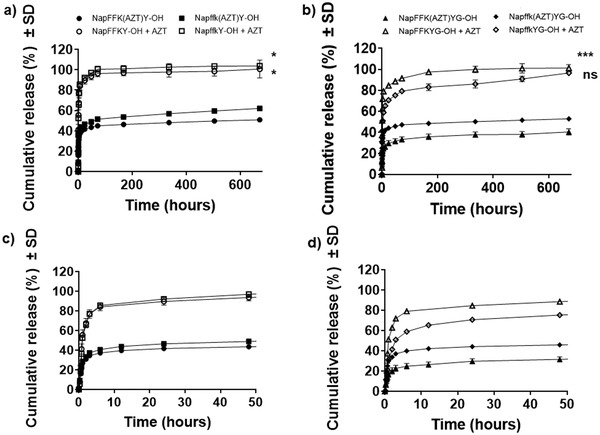
The cumulative release of zidovudine (AZT) physically encapsulated and chemically conjugated to D and L‐*α* peptide sequences in PBS over 28 days. Values represent the mean ± SD (*n* = 3). Key: black filled shape: drug conjugated to peptide, clear unfilled shape: drug physically encapsulated in peptide hydrogel. a) D and L‐*α* non‐glycine containing peptides, circle: NapFFK(AZT)Y‐OH, square: Napffk(AZT)Y‐OH, b) D and L‐*α* peptides with glycine spacer, Key: triangle: NapFFK(AZT)YG‐OH, diamond: Napffk(AZT)YG‐OH. ns: no significant (*p* ≥ 0.05), *: *p* < 0.05, **: *p* < 0.01, ***: *p* < 0.001, ****: *p* < 0.0001 difference between drug release from zidovudine conjugated peptide hydrogels and corresponding physically encapsulated controls. (c) and (d) data related to the first 48 h of drug release.

Each of the release profiles displayed in **Figure** **5** follow the typical trend described for in situ forming implants, consisting of initial burst release and a plateau with slower diffusion through the hydrogel matrix and hydrolysis of the peptide‐zidovudine covalent linker.^[^ ^61^ ^]^ A high degree of burst release was observed for physically encapsulated zidovudine with greater than 79% of the loaded drug released within the first 72 h. While release was observed over the entire 28 days, most of the drug had been released within the first three days. In clinical practice, such a result would raise concerns of initial dose toxicity, followed by potential sub‐therapeutic dosing and a difficulty in achieving therapeutically relevant concentrations throughout the ≈28 day dosing period. This is a well‐documented phenomenon for physically encapsulated systems.^[^ ^62^ ^]^ In certain therapeutic scenarios, this may be beneficial, for example, reaching a high dose in a short time period for pain relief, but often physical encapsulation is only suitable for localized, acute delivery applications. In the case of chemical conjugation to each peptide, zidovudine is conjugated to the lysine residue via ester linkage with release from the hydrogel matrix as a result of simple hydrolysis and subsequent diffusion into the surrounding fluid, in this case the release media. Upon hydrolysis of the drug‐ester linkage, zidovudine is released in a chemically unmodified form which is pivotal to the success of such technology since modification of the drug may alter the therapeutic action, render it ineffective, or result in the formation of a toxic metabolite thereby invalidating the strategy. Figure 5 shows that there is still an initial burst release from chemically conjugated systems; however, this is trend tends to be significantly lower than that observed for physically encapsulated zidovudine. The exception was NapffkYG‐OH, where the burst release from physically encapsulated zidovudine was 79.3% at 72 h. This was reduced by over 30% to 47.3% via chemical conjugation of zidovudine to the same gelator. Surprisingly for NapffkYG‐OH, drug release (physical encapsulation versus chemical conjugation) was shown to be non‐significant over the full 28 day profile. All other peptides demonstrated significant differences in zidovudine release.

The initial burst release may be attributed to the release of zidovudine close to or at the surface of the hydrogel matrix. Similarly, Nagai et al. found that self‐assembling hydrogels composed of RADA16 suffered from initial loss of incorporated dyes at the hydrogel–solvent interface due to increased porosity and suggested that this could be reduced by increasing the peptide fiber concentration to reduce the diffusivity of incorporated components.^[^ ^63^ ^]^ Interestingly, the release rate of zidovudine from the D‐variant in each case was higher than that of the corresponding L‐*α* variant but was non‐significant. It is also important to consider the simplicity of this in vitro release system since the lack of the presence of proteolytic enzymes is likely to have an effect on the release rate. As demonstrated, the D‐variants exhibit increased resistance to proteinase K and it is likely that the release rates for the L‐*α* variant may be much higher in an in vivo setting owing to enzymatic susceptibility. Table S5, Supporting Information, outlines the quantitative concentration and percentage cumulative release of zidovudine at 72, 168 and 672 h and 3, 7 and 28 days, respectively. It may be necessary to modify the sequences to tailor the release profile to the exact requirements. Peptides and peptide‐mimetics offer a vast choice of different chemical functional groups for drug attachment and the linker used to conjugate the drug to the peptide sequence can influence the release profile achieved.

The ideal drug delivery formulation would offer zero‐order kinetics in which the release of an active agent is only a function of time and the process takes place at a constant rate independent of the active agent concentration throughout the entire release period.^[^ ^64^ ^]^ KinetDS 3.0 software was used to model drug release, with the highest *R* ^2^ value for each release profile obtained using the Weibull model (Table S6, Supporting Information). The Weibull model is an empirical and generalized form of the exponential function and has been shown previously to be the most applicable model for drug release from nanoparticles, rather than hydrogels. For each sequence the *R* ^2^ values obtained were similar for the Ritger–Peppas model of drug release and this was deemed to be the most suitable model.^[^ ^65^ ^]^ Modelling of the zidovudine release profiles was therefore performed using the simple Power Law equation, which is essentially a semi‐empirical model establishing the exponential relationship between release and time (Table S7, Supporting Information). In situ forming peptide hydrogels typically adopt irregular geometries at the implant site, which are difficult to predict prior to injection and depend on the injection site and the volume of space. This increases model complexity, causing difficulty when trying to accurately represent the real system using mathematical modelling and may result in non‐uniform drug distribution within gels, which further increases the abstract nature of a mathematical construct.^[^ ^66^ ^]^ The *n* values obtained for each release profile varied from 0.88–0.96. All calculated *n* values were greater than 0.5 but less than 1 corresponding to non‐Fickian or so‐called anomalous transport which is thought to occur as a result of both Fickian diffusion and macromolecular chain relaxations.^[^ ^67^ ^]^ The release of zidovudine from these hydrogel systems therefore likely occurs as a result of both erosion of the hydrogel and diffusion through the matrix resulting in release from the hydrogel via anomalous transport.^[^ ^68^ ^]^

### 2.8 In Vivo Plasma Drug Concentration Studies

**Figure 6:**
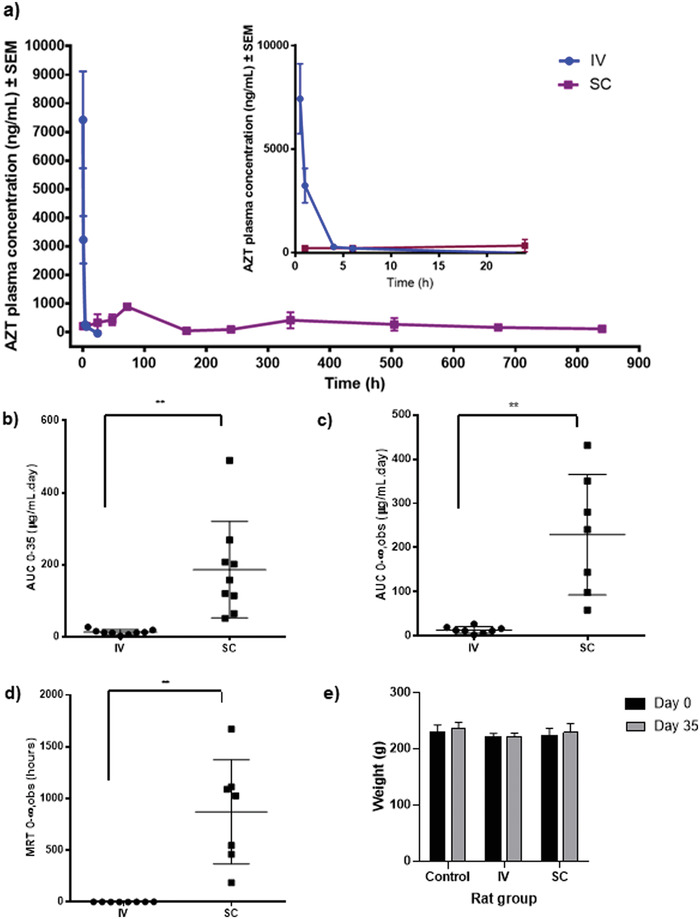
a) In vivo zidovudine (AZT) drug plasma concentrations obtained across 35 days in Sprague Dawley rats (*n* = 6) after intravenous administration of AZT only (no peptide) and subcutaneous administration of D‐peptide containing glycine variant Napffk(AZT)Y(p)G‐OH. Statistical analysis and representation of b) AUC _0‐35_, c) AUC _0‐∞_,_obs_, and d) MRT _0‐∞_,_obs_ in rats from both cohorts. Each point on the graph represents one rat (total *n* = 12). ((b)**p = 0.0046, (c) **p = 0.0058, (d) **p = 0.0038)). e) Weight of Sprague Dawley rats (*n* = 6) at the beginning (day 0) and end (day 35) of the experiment in the control, intravenous, and subcutaneous administration groups.

The in vivo study was conducted to compare zidovudine drug administered intravenously to our peptide formulation Napffk(AZT)YG‐OH administered via subcutaneous injection. Healthy female Sprague Dawley rats were chosen as a model for evaluating the systemic delivery of long‐acting formulations, with females selected specifically in anticipation of future studies that will include the use of combined contraceptives.^[^ ^69^ ^]^ Napffk(AZT)YG‐OH was selected for in vivo study due to its biostability as a D‐enantiomer (Figure 3d), sufficient gel strength (Figure 2c) and low toxicity (Figure 4a,b). **Figure** **6**a shows the plasma concentration of zidovudine over time and pharmacokinetic parameters are presented in Table S8, Supporting Information. Figure 6a shows that the plasma levels of zidovudine increased until 72 h and were then maintained within its IC_50_ range of 0.03–0.13 µg mL^−1^ over 35 days.^[^ ^70^ ^]^ The plasma concentration of intravenously administered zidovudine control was unsurprisingly initially high at 7.5 µg mL^−1^, 3.5 times higher than chemically conjugated zidovudine, but gradually decreased to undetectable levels after 6 h (Figure 6a). Conjugation of zidovudine to the peptide motif increased its half‐life from 1.7 to 674 h (Table S8, Supporting Information). The pharmacokinetic parameters calculated using PKSolver 2.0 are presented in Figure 6b–d. The AUC_o‐∞_ of zidovudine in Napffk(AZT)YG‐OH increased by 17 times (Figure 6c) and *C*_max_ was decreased by 9 times. The mean residence time (MRT) of zidovudine delivered via Napffk(AZT)YG‐OH was calculated to be 1089 h (Figure 6d), much higher than that of the intravenous control group (1.5 h). Rat weight and behavior in both treatment groups were monitored over the duration of the study and compared with a negative control cohort (healthy rats that did not receive drug treatment). Both intravenous and subcutaneous administrations were well tolerated. No deaths or serious adverse effects were observed and there was no apparent sign of irritation or infection at the injection site in either experimental group. Additionally, no significant differences in weight gain were observed between the control and experimental cohorts (Figure 6e), indicating that treatments were well tolerated and did not appear toxic. These in vivo results demonstrate that zidovudine, delivered subcutaneously by chemically conjugated Napffk(AZT)YG‐OH, has significant potential as an effective future drug delivery strategy in tackling HIV/AIDS infection. Our system improves the pharmacokinetic parameters of zidovudine compared to intravenous delivery. Future studies will be necessary to establish the rate of in vivo biodegradation of the hydrogel and correlate this with drug release to determine the correct dosing frequency for such a formulation. Seo et al. observed the biodegradability patterns of subcutaneous injectable hyaluronic based hydrogels by incorporating indocyanine dye into the precursor mixture prior to injection and near‐infrared fluorescence imaging was used at various time points and compared to free injection of the dye.^[^ ^71^ ^]^ One potential concern for repeated injection of non‐native d‐peptides is that the hydrogel itself may act as a vaccine adjuvant, resulting in the production of antibodies against the drug cargo. Wang and colleagues reported the successful coassembly of L/D Nap‐GFFY(p)‐OMe and ovalbumin and investigated the use of this peptide hydrogel as a potential vaccine adjuvant.^[^ ^30^, ^72^ ^]^ They proved that when either the L or D peptide hydrogel was assembled with the protein ovalbumin, it can strongly induce antibody production and the secretion of relevant cytokines in an in vivo model. However, the L/D Nap‐GFFY(p)‐OMe alone did not induce an immune response. This will warrant investigation in future studies as while our results to date are promising the potential to illicit an unwanted immune response must be considered on a case‐by‐case basis.

## 3 Conclusions

This work is a proof‐of‐concept, that a HIV/AIDS long‐acting drug delivery implant can be developed using an enzyme‐responsive peptide hydrogel system. This platform has significant potential to address both HIV/AIDS treatment and prevention. Proof‐of‐efficacy studies in HIV models will form an important part of future development. Current therapies fail to address the global burden of HIV/AIDS transmission due to poor patient compliance with complicated treatment regimens and the limitations of existing formulations. A major barrier to the clinical translation of new investigative treatments is that they often focus on a single agent, while multi‐drug regimens are required to successfully treat HIV/AIDS. This platform is amenable to the incorporation of different antiretroviral drugs targeting different stages in the replication cycle and is therefore promising in the development of an effective and convenient multi‐drug treatment strategy. This formulation is also a discrete therapy. This may be particularly advantageous for females if a partner is against treatment, for example due to cultural issues.^[^ ^73^ ^]^ Our advanced technology has the potential to be exploited for other controlled release drug delivery applications, especially for chronic diseases where patient adherence to medication is low (e.g., tuberculosis, schizophrenia, malaria, drug dependence). These results will inform the design of stimuli–responsive systems for the controlled delivery of drugs throughout the body (e.g., ocular, spinal, bone, medical devices) and wider science disciplines that utilize hydrogel platforms (e.g., 3D printing, cell culture).

## 4 Experimental Section

### Peptide Synthesis, Drug Conjugation, Purification, Identification, and Formulation

L‐*α* (NapFFKY(p)‐OH and NapFFKY(p)G‐OH) and D‐enantiomer (NapffkY(p)‐OH and NapffkY(p)G‐OH) peptide variants were synthesized using standard Fmoc solid‐phase peptide synthesis protocols as fully outlined within the Supporting Information.^[^ ^23^ ^]^ Wang resin was utilized to create carboxylic acid terminated sequences. Fmoc‐O‐phospho‐L‐tyrosine was directly anchored onto the Wang resin solid support using the dichlorobenzoyl chloride method outlined by Sieber.^[^ ^74^ ^]^ This acted as the phosphorylated enzymatic trigger for hydrogelation. L or D lysine was introduced to the peptide sequence to provide a side chain *ε*‐amino group that forms an ester cleavable linker to the hydroxyl grouping of the model HIV/AIDS antiretroviral zidovudine using a method previously outlined by the Xu group.^[^ ^17a^ ^]^ The addition and subsequent activation of succinic anhydride to zidovudine created a functional group that allows directed attachment (Figure S1, Supporting Information), via ester linkage, to the *ε*‐amino side group of the peptide. The structures of all synthesized sequences are shown in Figure S2, Supporting Information. The product was purified using a Gilson semi‐preparative HPLC (Gilson, Bedfordshire, UK) fitted with an XSelect CSH Prep C_18_ column (Waters, Herts, UK) and a mobile phase of Milli‐Q ultrapure water with 0.1% v/v trifluoroacetic acid (TFA) and acetonitrile (ACN), as fully outlined in the Supporting Information. Peptides were lyophilized using an Edwards freeze drier fitted with a RV8 pump (Davidson and Hardy, Belfast, Co. Antrim, UK) and products were identified using ^1^H‐NMR (Bruker Ultrashield Plus 400 MHz, Bruker, Coventry, UK) (Figure S5, Supporting Information). Electrospray Time of Flight Mass Spectrometry (ESI‐MS) (Waters LCT Premier, Waters, Hertfordshire, UK), with inbuilt Mass Lynx software to predict the theoretical mass, was performed and analyzed by Analytical Services and Environmental Projects (ASEP) in the School of Chemistry, Queen's University Belfast (Figure S3 and Table S1, Supporting Information). ^31^P‐NMR was also performed to monitor inclusion of the phosphate grouping into the peptide sequence (Figure S6, Supporting Information). HPLC chromatograms are shown in Figure S4, Supporting Information.

### Hydrogel Formulation, Gelation Propensity, and Mechanical Characterization

The gelation of peptides and peptide‐antiretroviral conjugates was tested over a range of concentrations (0.1–2 w/v%) in pH 7.4 phosphate buffered saline (PBS). The ability of alkaline phosphatase enzyme to trigger gelation was studied by adding 2 µL of 2 U (1000 U/mL) solution to the dissolved peptide‐PBS mixtures. Hydrogel formulation steps are outlined fully in Table S2, Supporting Information. A vial inversion assay was used as a screening tool to assess gel propensity, whereby peptide‐PBS‐alkaline phosphatase mixtures were inverted within glass HPLC vials. Gel formation and the mechanical strength of gels were definitively characterized via oscillatory rheology using an Anton Paar MCR302 rheometer (Anton Paar, St Albans, UK). A vane and cup geometry was used to perform frequency and strain sweeps. Gels were formulated as 2 mL volumes in 7 mL Sterilin vials as previously described.^[^ ^39b^ ^]^ Frequency sweeps were performed at 37 °C from 1–100 rad/s at a strain of 0.5%. Amplitude sweeps were performed at 37 °C, 2.5 rad/s and a strain of 0.01–1000%. Flow point or breakage strain, where G″ ≥ G′ within the strain sweeps, was used as a measurement of gel strength. Time sweeps were performed using a sand blasted parallel plate PP50/S system at 37 °C with a 0.5 mm measuring gap over a period of 24 h. Loss and storage moduli, critical strain, viscosity, and oscillatory time sweeps were performed to relate rheological changes with time and potential for rapid in situ hydrogel implant formation upon exposure to 2 U phosphatase enzyme.

### Microscopy and Spectroscopy

Hydrogel nanoscale architecture was studied using scanning electron microscopy (JEOL JSM 6500 F SEM [JEOL, Freising, Germany]). 80 µL of 2 w/v% formulated peptide hydrogels were pipetted onto an SEM sample mount, flash frozen in liquid nitrogen, and then lyophilized overnight. SEMs were imaged at 3 kV and each sample pre‐coated with an 8 nm layer of gold.^[^ ^75^ ^]^ TEM imaging was performed on a Joel JEM 1400 Plus Transmission Electron Microscope. Samples were also prepared according to hydrogel formulation steps fully outlined in Table S3, Supporting Information, and 3 µL of each was placed on a copper grid (sufficient to cover the grid surface). The grid was then left to air dry and immediately examined. Circular dichroism was performed at 0.5 w/v% (solubilized) and at 2 w/v% (hydrogels) using a Jasco J815 Spectropolarimeter (Jasco, Easton, Maryland, USA) and a 3 µL sub‐micro quartz cuvette 0.01 mm path length (Starna Scientific Ltd, Essex, UK) (1 s integrations, 1 accumulation, step size 1 nm, band width 1 nm, wavelengths 180–300 nm, 25 °C).^[^ ^23^ ^]^ To ensure reliability, the high‐tension voltage (HT) value was monitored and kept below 600 V. Measurements were repeated three times and the mean values were plotted. Control samples (PBS) did not display any signal.

### Small Angle Neutron Scattering (SANS)

SANS was used to determine the morphology of the structures underpinning the hydrated peptide gel network. Both drug and non‐drug conjugated forms of peptides L‐*α* (NapFFKY‐OH and NapFFKYG‐OH) and D‐enantiomer (NapffkY‐OH and NapffkYG‐OH) were studied. Peptide hydrogels (2 w/v%) were formulated as described (Table S2, Supporting Information) in 2 mm path length UV spectrophotometer grade quartz cuvettes (Hellma) but using deuterated water (D_2_O) rather than PBS. SANS measurements were performed using the D11 instrument at the Institut Laue – Langevin, Grenoble, France. Scattering from the primary assembled structures (most commonly fibers) and the network were collected over a wide Q range (Q = 4*π*sin(*θ*/2)/*λ*) of 0.001 to 0.5 Å^−1^ and three sample‐detector distances (1.4, 8, and 39 m).^[^ ^44^ ^]^ Peptide samples were placed in a temperature‐controlled sample rack during measurements. The datasets obtained were reduced to 1D scattering curves of intensity versus Q using the facility provided software. Scattering from D_2_O controls, empty cuvette, and electronic background were subtracted from the data and the full detector images were normalized. SasView software version 5.0.4 was used to fit instrument‐independent data to several models with and without a power law applied, including a cylinder, an elliptical cylinder, a flexible cylinder, a flexible elliptical cylinder and hollow cylinders. Utilizing chi‐squared, the best fitted model for peptide hydrogels was the flexible cylinder elliptical model with Power Law applied. The chi‐squared value was still relatively high in this case and some flexibility was needed. A summary of the fitting parameters is shown in Table S4, Supporting Information. The data and the fits are shown in Figure S17, Supporting Information.

### Biostability

Peptides were tested for stability at multiple time points over 28 days by incubation with the broad‐spectrum protease proteinase K. Peptides were tested in solution form at concentrations below their critical gelation concentrations (0.02 w/v%) to reduce any resistance to proteolysis offered by the hydrogel matrix itself.^[^ ^76^ ^]^ 1 mL aliquots of peptide dissolved in PBS buffer were added to Eppendorf tubes. Proteinase K was added at a concentration of 3 U/mL and incubated at 37 °C. Then 100 µL samples were removed at pre‐determined time points (*t* = 0, 0.5, 1, 3, 6, 24, 72, 168, 336, 504, and 672 h) and added to 50 µL of concentrated acetic acid to inactivate proteinase K enzyme. Then 100 µL samples were then analyzed via RP‐HPLC. The amount of peptide remaining at each time point was calculated based on the area of the peak corresponding to the peptide and presented as a percentage of the area of this peak at *t* = 0 (Equation (1)).

(1) $$\begin{array}{c} \%\;\mathrm{Peptide}\;\mathrm{remaining}\;=\;\frac{\left[\mathrm{Area}\;\mathrm{of}\;\mathrm{peptide}\;\mathrm{peak}\;\mathrm{at}\;\mathrm{test}\;\mathrm{timepoint}\right]}{[\mathrm{Area}\;\mathrm{of}\;\mathrm{peptide}\;\mathrm{peak}\;\mathrm{at}\;t=0]}\;\times \;100 \end{array}$$

### Cell Cytotoxicity and Biocompatibility

Cell toxicity was assessed above and below critical gelation concentrations utilizing the International Standard (ISO) murine fibroblast subcutaneous connective tissue cell line NCTC 929 (ATCC CCL 1).^[^ ^51^ ^]^

Preparation of tissue cell cytotoxicity assays: Cells were cultured in Minimum Essential Medium (MEM) containing phenol red, Earle's Salts, and L‐glutamine supplemented with 10% fetal bovine serum and 1 mm sodium pyruvate (Invitrogen, Paisley, UK) and grown at 37 °C/5% CO_2_. Cells were subcultured at 80–90% confluency via removal of media, washing with sterile PBS, and detachment of cell monolayers with 0.05% trypsin/0.53 mm EDTA.4Na solution (Invitrogen, Paisley, UK). Cells were cultured until at least the third passage, seeded at 1 × 10^4^ cells per well in sterile 96‐well microtiter plates for 6 h, 24 h, and 56 day treatment times and seeded at 5 × 10^3^ cells per well for 72 h treatment times. After 24 h of incubation, the media was removed and the cells were exposed to 100 µL of peptide‐drugs across a range of concentrations (soluble peptide: 20–500 µm, hydrogel forming range: 0.5–2 w/v%). Control wells included PBS or media (100% viability, negative control) and 70% ethanol treated cells (100% kill, positive control).

Following exposure to peptide after 6, 24 and 72 h, cell viability was assessed using a MTS based CellTiter 96 AQueous One Solution Cell Proliferation Assay (Promega, Southampton, UK). Absorption was measured at 490 nm using a Tecan Sunrise plate reader (Tecan UK Ltd, Reading, UK). Cell viability was calculated using Equation (2) below and reported as the mean of nine replicates.

(2) $$\begin{array}{ccc} \%\;\mathrm{Metabolic}\;\mathrm{activity} & = & \frac{\left[\left({\mathrm{Abs}}_{490\mathrm{nm}}\mathrm{Experimental}\;\mathrm{well}\;-\;\mathrm{Ab}s_{490\mathrm{nm}}\mathrm{Media}\right)\right]}{\left[\left({\mathrm{Abs}}_{490\mathrm{nm}}\mathrm{Negative}\;\mathrm{control}\;-\;\mathrm{Ab}s_{490\mathrm{nm}}\mathrm{Media}\right)\right]}\\ & & \times \;100 \end{array}$$

A Cytotoxicity Detection Kit_PLUS_ assay (Sigma‐Aldrich, Dorset, UK) was used to quantify lactate dehydrogenase (LDH) release as previously outlined.^[^ ^39a^ ^]^ LDH acted as a marker for cell cytotoxicity and was rapidly released from the cell upon disruption of the cell membrane. After 6 h of incubation with peptide, 100 µL of test reagent was added to each well and incubated for 5 min after which 50 µL of the stop reagent was added and the absorbance was measured at 490 nm. A 100% toxicity high LDH release control was created by addition of 5 µL of lysis solution to non‐peptide treated wells 15 min before the addition of test reagent. Three additional experimental controls were required alongside background controls: i) the peptide‐drug at the highest concentration (500 µm) control in media; ii) LDH standard in media; and iii) high peptide‐drug concentration (500 µm) in media with an equal volume of LDH standard. No significant addition or reduction was observed in the absorbance for each of these three controls. Cell viability was calculated using Equation (3) below and reported as the mean of nine replicates.

(3) $$\begin{array}{ccc} & & \%\;\mathrm{Cytotoxicity}\\ & & \;=\frac{\left[\left({\mathrm{Abs}}_{490\mathrm{nm}}\mathrm{Experimental}\;\mathrm{well}\;-\;\mathrm{Ab}s_{490\mathrm{nm}}\mathrm{Low}\;\mathrm{LDH}\;\mathrm{release}\right)\right]\;}{\left[\left({\mathrm{Abs}}_{490\mathrm{nm}\;}\mathrm{High}\;\mathrm{LDH}\;\mathrm{release}\;-\;\mathrm{Ab}s_{490\mathrm{nm}}\mathrm{Low}\;\mathrm{LDH}\;\mathrm{release}\right)\right]}\\ & & \;\times \;100 \end{array}$$

A Live/Dead Viability/Cytotoxicity fluorescent assay (Thermo Fisher Scientific, Waltham, MA, USA) was used to quantify the viability of NCTC 929 cells with fluorescence microscopy (EVOS FL microscope, Thermo Fisher Scientific, Waltham, MA, USA). Following a 24 h incubation with peptides and peptide‐drug conjugates (20–500 µm), NCTC 929 cells were exposed to a mixture of 4 µm ethidium homodimer‐1 and 2 µm calcein AM in PBS for 20 min. Each experiment was performed in triplicate at each concentration tested and the number of live and dead cells in each image was calculated using ImageJ 1.52a software (National Institutes of Health, Maryland, USA). To do this, images were taken using green fluorescent protein and Texas Red filters individually. The background was subtracted and the threshold was adjusted to remove any remaining background pixels, leaving only the cells. The image was then converted to a mask and watershed applied to separate any touching cells and then counted using the automated ImageJ analyze particles plugin. In the case of dead cells, the size in pixels included 50‐infinity pixels and for live cells 120‐infinity pixels with 0.00–1.00 circularity were employed in each case.

Biocompatibility at the highest gel concentration of 2.0 w/v% was examined by measuring cell metabolic activity (MTS) and cytotoxicity (LDH release) in the presence of media containing leached degradation products from the peptide gels over a period of 56 days.^[^ ^77^ ^]^ Following the formation of gels as outlined in Table S2, Supporting Information, 2 mL of media was added on top of each gel (1 mL of 2 w/v% gel) within a 10 mL glass vial and incubated at 37 °C and 5% CO_2_. For each time point in the 56 day study, NCTC 929 cells at 80–90% confluency were seeded into the wells of a 96 well plate at a concentration of 1 × 10^4^ cells per well and incubated for a period of 24 h. One set of wells was left untreated and acted as the negative control, that is, metabolic activity of cells with no peptide. In the case of the LDH indirect toxicity assay, the media was removed from the cells before adding 50 µL of fresh media and 50 µL of the supernatant from the 2.0 w/v% gels at specific time points: 1, 3, 7, 14, 21, 28, and 56 days and incubated for 6 h before adding the test reagent as described above. A high LDH release control and a low LDH release control were created as before. Each experiment was performed in triplicate. The percentage cytotoxicity was calculated using Equation (2) above. For the MTS indirect toxicity assay, 100 µL of the supernatant from the 2 w/v% gels was removed at specific time points: 1, 3, 7, 14, 21, 28 and 56 days, added to the cells and incubated for 6 h before replacing the media with fresh media and the MTS reagent as described above. Each experiment was performed in triplicate. The metabolic activity was calculated using Equation (1) above.

The effect of each peptide on equine erythrocytes was examined using a method previously outlined by Shin et al.^[^ ^57^ ^]^ 100 µL of erythrocytes, diluted to 4% v/v in PBS, were incubated in the wells of a 96 well plate with 100 µL of each of the peptide‐drug conjugates (20–500 µm) after dissolving in PBS for 1 h at 37 °C. Control wells included 1% v/v Triton X‐100 (positive control) and PBS (negative control). After 1 h, each well was pipetted into an Eppendorf tube and centrifuged at 1000 *g* and aliquots of the supernatant were added to a fresh 96 well plate. The plate was read at 405 nm using a Tecan Sunrise plate reader (Tecan UK Ltd, Reading, UK) and hemoglobin release was determined using Equation (4) below. Each experiment was performed in triplicate.

(4) $$\begin{array}{c} \%\;\mathrm{Hemolysis}=\frac{\left[\left({\mathrm{Abs}}_{405\mathrm{nm}\;}\mathrm{experimental}\;\mathrm{well}\;-\;\mathrm{Ab}s_{405\mathrm{nm}}\mathrm{PBS}\right)\right]}{[(\mathrm{Ab}s_{405\mathrm{nm}}\mathrm{Triton}\;X\;-\;\mathrm{Ab}s_{405\mathrm{nm}}\mathrm{PBS})]}\times 100 \end{array}$$

### In Vitro Drug Release

The release of drug from peptide hydrogels was evaluated in PBS (pH 7.4, 37 °C) under sink conditions at multiple time points over 28 days (1, 2, 4, 8, 24, 48, and 72 h, followed by daily). Drug release was examined as: i) physical mixture/encapsulation between each peptide hydrogel and drug, and ii) after chemical conjugation of zidovudine to the peptide sequences. For (i) a weighed quantity of lyophilized gelator was mixed with drug to a final concentration of 20% and the resulting mixture was used to form a hydrogel at a concentration of 2 w/v% as outlined in Table S2, Supporting Information. Physical encapsulation was therefore achieved concurrently with hydrogelation. For (ii) the hydrogel was formulated as per Table S2, Supporting Information. Each hydrogel was formulated in triplicate, with 100 µL added to each vial prior to the addition of ALP. Upon gelation, 1 mL of temperature equilibrated (37 °C) PBS was added to the top of the gel. Vials were incubated in a thermostatic shaking water bath at 37 °C and 50 rpm with 200 µL of supernatant removed at pre‐determined time points and replaced with 200 µL fresh, temperature‐equilibrated PBS to maintain sink conditions. The concentration of drugs released was determined by analytical HPLC in line with British Pharmacopoeial methods (C_18_ gel column, UV spectrophotometry detection wavelength 270 nm).^[^ ^25^ ^]^

### In Vivo Plasma Drug Concentration Studies

A 35 day zidovudine plasma concentration study was conducted in Sprague Dawley rats. This was extended from 28 days to determine the feasibility of a longer dosage interval. Approval for the animal studies was obtained from the Queen's University Belfast Ethics Committee of the Biological Services Unit (application: HM_2022_08) under a UK Home Office Project License (PPL2903). Female Sprague Dawley rats (total *n* = 18) were procured from Envigo, aged 8–10 weeks, with a mean weight of 220.72 ± 11.31 g. They were acclimatized to the animal house conditions for a week prior to the experiment. Animals were separated into two experimental cohorts, each comprising 6 rats with a third cohort left as an untreated, healthy control. The sample size was calculated using the La Mortes Power Calculation as outlined in the Supporting Information. The first cohort received zidovudine by intravenous bolus administration to the tail vein at a dose of 8.46 mg kg^−1^ as per literature.^[^ ^70^ ^]^ The intravenous injection was freshly prepared aseptically by dissolving the required mass of zidovudine in sterile water for injection BP in a laminar flow cabinet. The second cohort received 600 µL of 2 w/v% Napffk(AZT)Y(p)G‐OH peptide solution, containing 8.46 mg kg^−1^ of zidovudine via subcutaneous administration. A Napffk(AZT)Y(p)G‐OH injectable solution was formulated by sterilizing lyophilized peptide under UV before dissolving in sterile water for injection and adjusting the pH to 7.4 using 0.1 m NaOH. Blood samples (≈0.2 mL) were collected into 1.5 mL pre‐heparinized microtubes over 48 h for the intravenous cohort and for 35 days in the subcutaneous cohort by tail vein bleeds. Blood plasma was separated by centrifugation at 13 400 rpm for 10 min and the resultant supernatants were collected and stored at −80 °C until further analysis. The zidovudine concentration in blood plasma samples was analyzed by RP‐HPLC as outlined in the Supporting Information. The weight of each rat in all three cohorts was measured at day 0 and day 35 to give an indication of any toxic effects induced by treatments. Pharmacokinetic parameters were calculated using PKSolver 2.0, namely, the half‐life (*t*_1/2_), the time to peak drug concentration (*T*_max_), the maximum plasma concentration (*C*_max_), the area under the plasma concentration–time curve from time zero to the last quantifiable concentration at day 35 (AUC_0−35_), the observed area under the plasma concentration–time curve extrapolated from time zero to infinity (AUC _0‐∞,obs_) and the mean residence time (MRT) of the drug extrapolated from time zero to infinity.

### Statistical Analysis

Statistical analyzes were performed using Microsoft Excel 2016 and GraphPad Prism 9. Standard deviations were obtained at each concentration of peptide tested based on three replicates for biostability and nine replicates for quantitative cell cytotoxicity assays and mean values were obtained. Biostability was compared using a Kruskal–Wallis test with Dunn's post‐hoc to identify individual differences compared to non‐treated peptide‐only (100%) control. Cell cytotoxicity (MTS, LDH, hemolysis) was also compared using a Kruskal–Wallis test with Dunn's post‐hoc, with percentage cell viability compared to the media only (MTS, LDH) and PBS (hemolysis assay) controls. Similarly, a Kruskal–Wallis test was employed to compare physically encapsulated and covalently attached zidovudine release within in vitro drug release assays with a Dunn's post‐hoc test used to identify individual differences in the data (within the same or across different timepoints). A Kruskal–Wallis test was employed when data was shown not to be normally distributed using the Kolmogorov and Smirnov method. In all cases, a probability of *p* ≤ 0.05 denoted significance. KinetDS 3.0 software (SourceForge Media, La Jolla, CA, USA) was utilized to model in vitro drug release kinetics and identify the mathematical model that best fits to release data. In vivo pharmacokinetic parameters were computed by a non‐compartmental model applying the program PKSolver 2.0.^[^ ^78^ ^]^

## Conflict of Interest

The authors declare no conflict of interest.

## Supporting information

Supporting Information

## Data Availability

The data that support the findings of this study are openly available in Enzyme triggered l‐*α*/d‐peptide hydrogels as a long‐acting injectable delivery platform for HIV/AIDS at https://doi.org/10.17034/67d0cd6f‐5bed‐4148‐b986‐f43975b0392c, reference number 1.
