# Supplementary material for: Enzyme‐Triggered l‐α/d‐Peptide Hydrogels as a Long‐Acting Injectable Platform for Systemic Delivery of HIV/AIDS Drugs
Source: Adv Healthc Mater. 2023 Mar 17;12(18):2203198. doi: 10.1002/adhm.202203198 (PMC11469249; doi:10.1002/adhm.202203198)
Supplement: Supplementary file 1 — Supporting Information [file ADHM-12-2203198-s001.pdf]

ADVANCED HEALTHCARE MATERIALS

Supporting Information

for *Adv. Healthcare Mater.*, DOI 10.1002/adhm.202203198

Enzyme-Triggered L-α/D-Peptide Hydrogels as a Long-Acting Injectable Platform for Systemic Delivery of HIV/AIDS Drugs

*Sophie M. Coulter, Sreekanth Pentlavalli, Lalitkumar K. Vora, Yuming An, Emily R. Cross, Ke Peng, Kate McAulay, Ralf Schweins, Ryan F. Donnelly, Helen O. McCarthy and Garry Laverty**

Supplementary Information

Enzyme-triggered L-α/D-peptide hydrogels as a long-acting injectable platform for systemic delivery of HIV/AIDS drugs

Sophie M. Coulter [a#], Sreekanth Pentlavalli [a#], Lalitkumar K. Vora [a], Yuming An [a], Emily R. Cross [a], Ke Peng [a], Kate McAulay [b, c], Ralf Schweins [d], Ryan F. Donnelly [a], Helen O. McCarthy [a], Garry Laverty [a,*].

[a] School of Pharmacy, Queen's University Belfast, Medical Biology Centre, 97 Lisburn Road, Belfast, N. Ireland, BT9 7BL.

[b] School of Chemistry, University of Glasgow, Joseph Black Building, Glasgow, Scotland, G12 8QQ.

[c] School of Computing, Engineering and Built Environment, Glasgow Caledonian University

[d] Large Scale Structures Group, Institut Laue – Langevin, 71 Avenue des Martyrs, CS 20156, 38042 Grenoble Cedex 9, France

[#] equal author contribution

Experimental procedures

Materials and apparatus

Synthesis: Sintered glass funnels and round bottomed flasks were purchased from VWR (VWR, Lutterworth, Leicestershire, UK). Resins, including Wang resin (100/200 mesh particle size, 1.1 mmol/g loading) and Fmoc-Gly-Wang resin (100/200 mesh particle size, 0.4 -0.8 mmol/g loading), and Fmoc protected amino acids including Fmoc-Tyr(PO_3H_2)-OH, Fmoc-Phe-OH, Fmoc-(D)Phe-OH, Fmoc-Lys(Boc)-OH, Fmoc-(D)Lys(Boc)-OH and Fmoc-Gly-OH were purchased from Sigma Aldrich (Sigma Aldrich, Gillingham, Dorset, UK). Dimethylformamide (DMF), anhydrous DMF, 1-hydroxybenzotriazole hydrate (HOBt), 2-(1H-benzotriazol-1-yl)-1,1,3,3-tetramethyluronium hexafluorophosphate (HBTU), diisopropyl ethylamine (DIPEA), methanol, anhydrous pyridine, tert-butylammonium fluoride (TBAF), hexane, triethylamine (TEA), imidazole, dichlorobenzoylchloride, N'N'-diisopropylcarbodiimide (DIC), piperidine, potassium cyanide, ninhydrin, phenol, 4-dimethyl-aminopyridine (DMAP), trifluoroacetic acid (TFA), dichloromethane (DCM), diethyl ether, acetone, sodium bicarbonate, anhydrous sodium sulfate, chloroform, hydrochloric acid, ethyl acetate and N-hydroxysuccinimide (NHS), were obtained from Fluorochem Ltd. (Hadfield, UK). Succinic anhydride, benzylamine, triisopropylsilane (TIPS) and thioanisole were purchased from Alfa Aesar (Alfa Aesar, Heysham, Lancashire, UK). 2-Naphthaleneacetic acid (Nap) and zidovudine (1-[(2R,4S,5S)-4-azido-5-(hydroxymethyl)oxolan-2-yl]-5-methylpyrimidine-2,4-dione) were obtained from TCI Chemicals (Tokyo Chemical Industry UK Ltd, Oxford, UK). Büchi rotavapor (Büchi UK Ltd., Chadderton, Oldham, UK).

Methods

Solid Phase Peptide Synthesis: First, 1g of preloaded Gly-Wang or Wang resin was suspended in DMF and agitated for 30 minutes with nitrogen flow through the resin bed. This was rinsed three times with 15 mL DMF.

Deprotection of the Fmoc protecting group: Deprotection was achieved by bubbling the resin in 20% piperidine in DMF for 30 minutes. The products of this deprotection reaction were then removed by rinsing three times with 15 mL DMF at each rinse.

Amino acid chain elongation: Four molar equivalents of Fmoc protected amino acid, 4 molar equivalents of HBTU (activator), 4 molar equivalents of HOBt and 8 molar equivalents of DIPEA (activator base) were dissolved in DMF and reacted for two hours by bubbling under nitrogen. This was followed by three DMF rinses. These steps were repeated in a series of deprotection and addition cycles until the desired peptide sequence was achieved. At each step a Kaiser test (potassium cyanide, ninhydrin and phenol) was performed as a qualitative test for the presence/absence of free amino groups.[1] Once the desired sequence was synthesized, the peptide was rinsed three times with DMF, five times with DCM and five times with methanol and dried overnight in a desiccator.

Cleavage from resin: For Wang resin conjugated peptides, a cleavage cocktail consisting of 95% TFA, 2.5% TIPS and 2.5% thioanisole was employed. The peptide was stirred gently using a magnetic flea, with the cleavage cocktail, in a round bottomed flask for two to three hours and washed three times with TFA. Solvents were removed via rotary evaporation using a Büchi rotavapor and precipitated using cold diethyl ether.

Anchoring Fmoc-amino acids to Wang resin: For glycine terminated sequences preloaded Wang resin was employed. In the case of Fmoc-O-phospho-L-tyrosine, the carboxylic acid terminal was directly anchored onto the Wang resin solid support using the dichlorobenzoyl chloride method documented in 1987 by Sieber.[2] This method was chosen since it reduces the likelihood of racemization and the formation of dipeptide side products, which are often encountered when using dicyclohexylcarbodiimide (DCC) and 4-dimethylaminopyridine (DMAP). Glycine is particularly at risk of dipeptide formation during loading to a hydroxy-functionalized resin if a symmetrical anhydride is used, so it is prudent to use the dichlorobenzoyl chloride method to avoid this.[3] The method is as follows. Wang resin was soaked in DMF for 30 minutes, 5 molar equivalents of Fmoc-protected amino acid, 8.25 molar equivalents of anhydrous pyridine and 5 molar equivalents of dichlorobenzoyl chloride were added to a sealed reaction vessel, and the mixture was agitated gently for 18 hours, with the aid of a magnetic flea, at room temperature under inert conditions. The product is then rinsed three times with DMF, and the series of deprotection and chain elongation steps can proceed in a sintered funnel, as normal.

Zidovudine conjugation: To covalently attach zidovudine to each of the L/D peptides, a series of steps were followed in a method adopted from Li.[4] The synthetic route for each conjugation is shown in Figure S1.

Zidovudine conjugation

Step 1. 3.5 molar equivalents of succinic anhydride and 1.6 molar equivalents of 4-dimethyl-aminopyridine (DMAP) were added to 1 molar equivalents of zidovudine in 10 mL of dry pyridine in a sealed reaction vessel and agitated gently for 12 hours with the aid of a magnetic flea at room temperature under inert conditions. After 12 hours, the product was extracted with chloroform (200 mL) and rinsed consecutively with 1.0 M hydrochloric acid (3 x 50 mL), water (50 mL) and brine (50 mL). The organic layer was dried using anhydrous sodium sulfate and concentrated using rotary evaporation. The residue was purified by column chromatography on 400 mesh silica gel (1:0 – 10:1 DCM/methanol).

Step 2. One molar equivalent of N-hydroxysuccinimide (NHS) and 1 molar equivalent of DIC were added to the product from step 1 in chloroform (10 mL) and the mixture was stirred at room temperature for 6 hours. Upon completion of the reaction, the mixture was filtered to remove N,N'-diisopropylurea (DIU) and the filtrate was concentrated using a Büchi rotavapor. The remaining residue was redissolved in 2 mL of acetone.

Step 3. Each of the peptide sequences to which zidovudine was to be conjugated was added to an aqueous solution (volume 3 mL, pH adjusted to ~7.6 with sodium bicarbonate ($NaHCO_3$)) and the product, dissolved in acetone, from step 2 was added dropwise. The resulting solution was stirred for 3 hours at room temperature. Upon completion of the reaction the product was freeze dried.

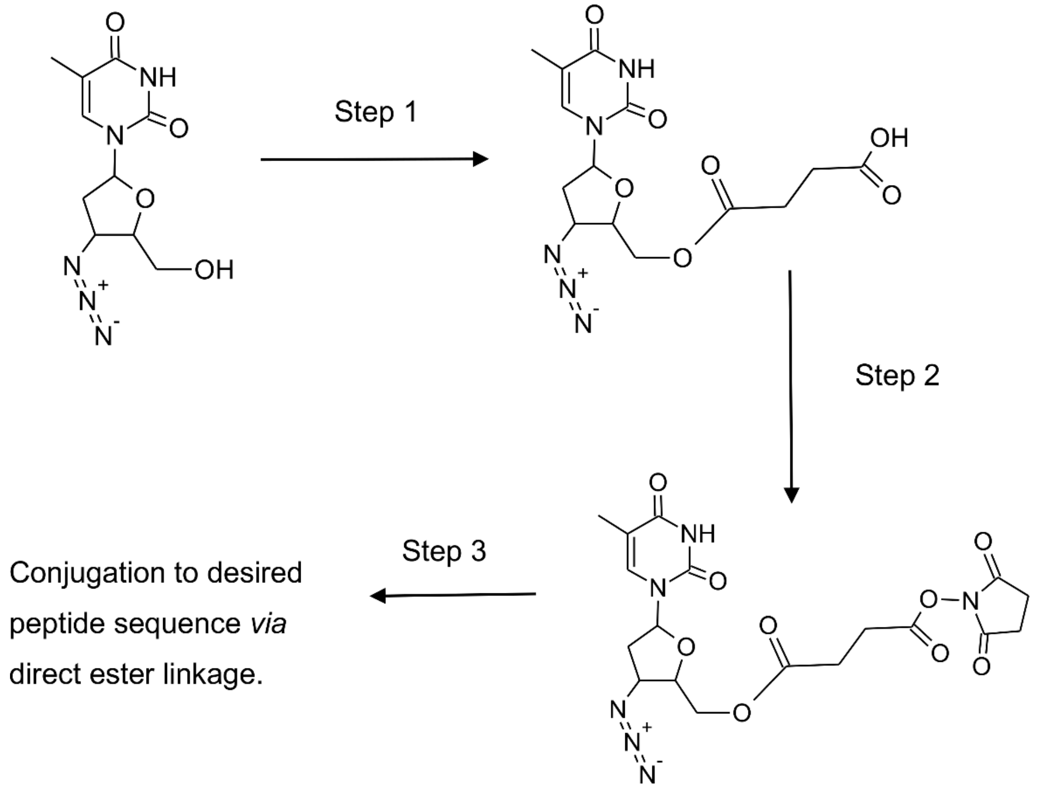

Figure S1. Synthetic scheme to show the preparation of zidovudine, to generate a functional group that allows direct attachment (step 3) to the lysine side chain to form an ester linkage with the desired peptide sequence. Step 1. Succinic anhydride, DMAP, anhydrous pyridine (12 hours), Step 2. NHS, DIC in chloroform (6 hours), upon completion filter to remove DIU, Step 3. $NaHCO_3$ (to pH 7.6), water, acetone and peptide (2 hours).

NMR: A total of 5-10 mg of sample was dissolved in DMSO-d_6 in a 5 mm NMR sample tube and ^{1}H-NMR and ^{31}P-NMR were performed across 256 scans using tetramethylsilane (TMS) as the internal standard at a frequency of 400 MHz.

Semipreparative HPLC: Peptide and peptide-drug conjugates were purified to ≥95% purity using semipreparative HPLC. Samples were dissolved in methanol to a final volume of 4 mL for each run and injected into a Gilson Preparative HPLC fitted with an XSelect® CSH™ Prep C_{18} column with a guard cartridge. Gradient: starting from 90% Milli-Q ultrapure water (0.1% v/v TFA) and 10% v/v acetonitrile (ACN) (0.1% v/v TFA) then ramping up to 35% v/v ACN at 7 minutes then ramping up to 60% v/v ACN at 18 minutes then ramping up to 100% v/v ACN at 22 minutes before ramping down to 10% ACN at 28 minutes. The column temperature was 25 °C and the flow

rate was 1.0 mL/min. Each of the fractions was collected and any solvent remaining was removed by rotary evaporation before freeze drying to remove water. Each fraction was then tested using ^{1}H-NMR and ESI-MS to determine the correct product before examination via analytical RP-HPLC.

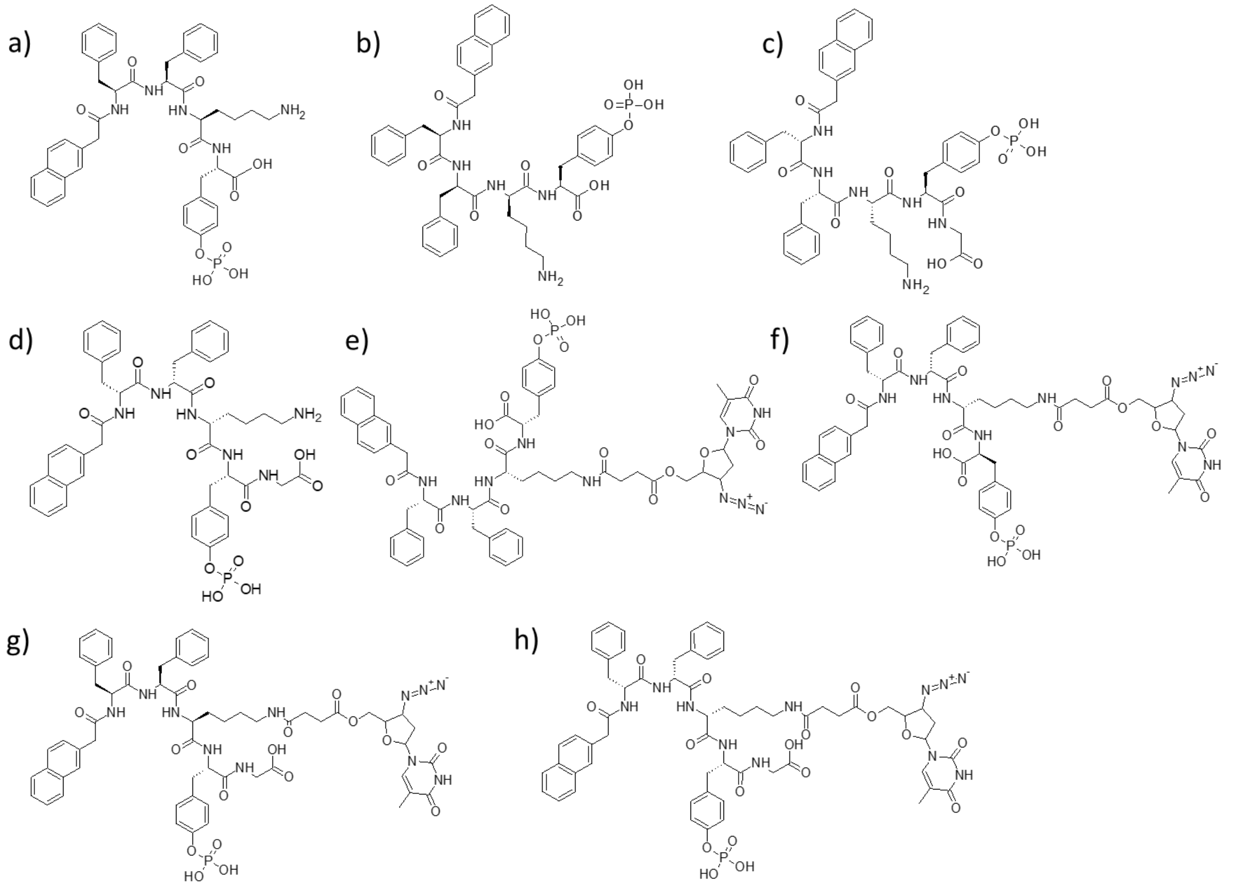

Figure S2. L- and D- enantiomer peptide sequences synthesized. Single letter amino acid nomenclature employed (upper case letter refers to L-α-amino acid and lower-case letter refers to D-α-amino acid). a) NapFFKY(p)-OH, b) NapffkY(p)-OH, c) NapFFKY(p)G-OH, d) NapffkY(p)G-OH, e) NapFFK(AZT)Y(p)-OH, f) Napffk(AZT)Y(p)-OH g) NapFFK(AZT)Y(p)G-OH, h) Napffk(AZT)Y(p)G-OH.

ESI-MS

a) i

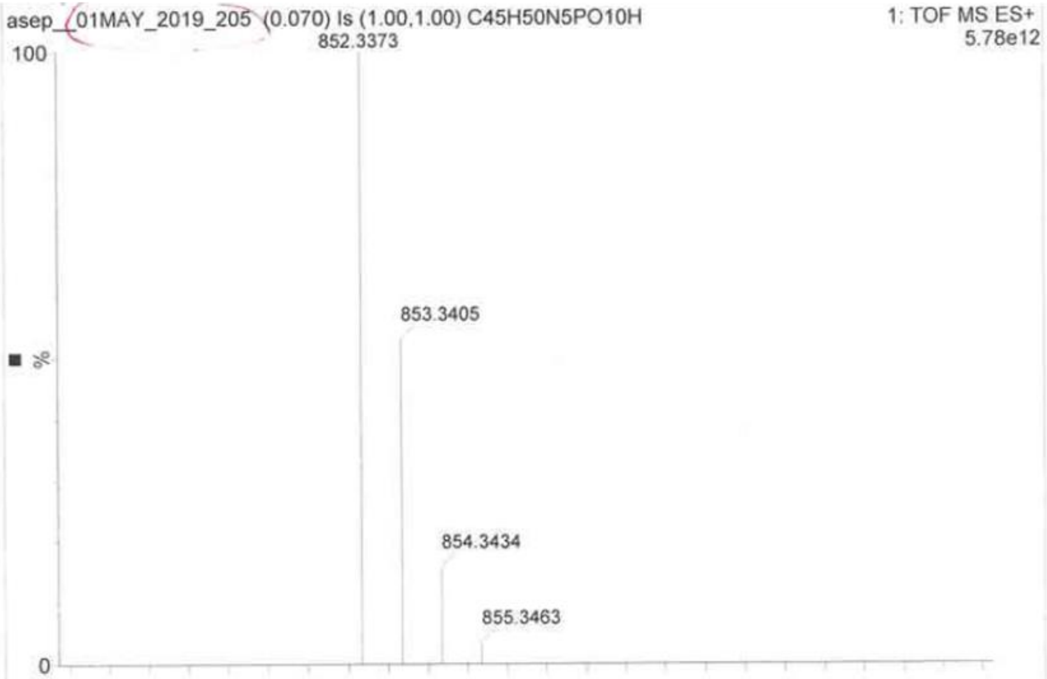
asep__01MAY_2019_205 (0.070) Is (1.00,1.00) C45H50N5PO10H
1: TOF MS ES+
5.78e12
100
852.3373
853.3405
%
854.3434
855.3463
0

ii

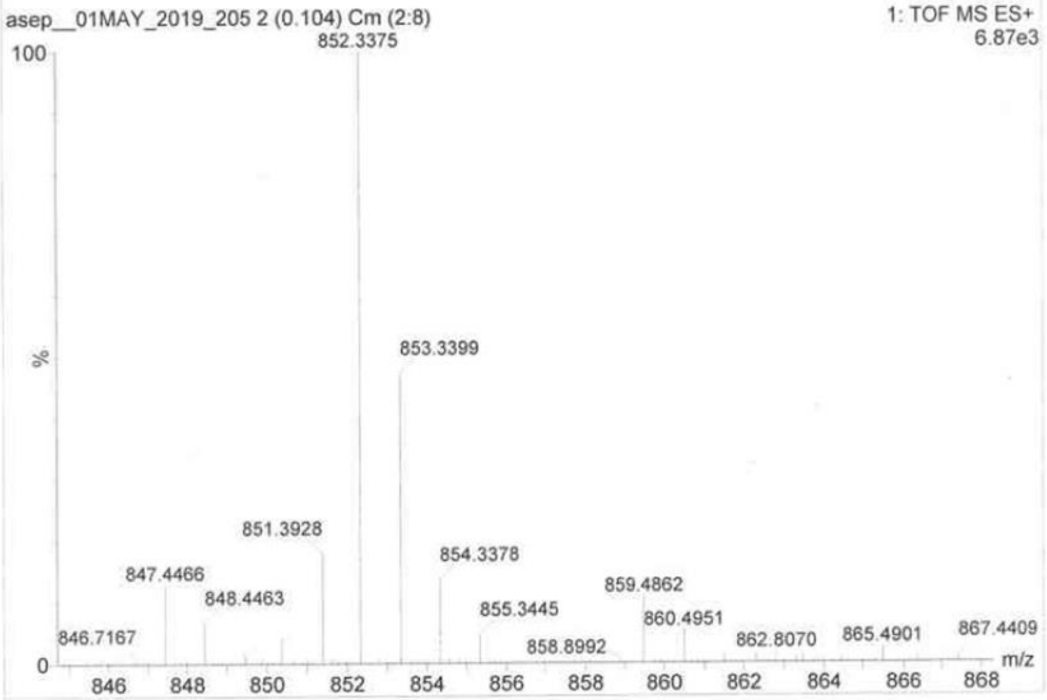
asep__01MAY_2019_205 2 (0.104) Cm (2:8)
1: TOF MS ES+
6.87e3
100
852.3375
853.3399
%
851.3928
847.4466
854.3378
848.4463
859.4862
855.3445
860.4951
846.7167
858.8992
862.8070
865.4901
867.4409
0
m/z
846
848
850
852
854
856
858
860
862
864
866
868

b) i

ii

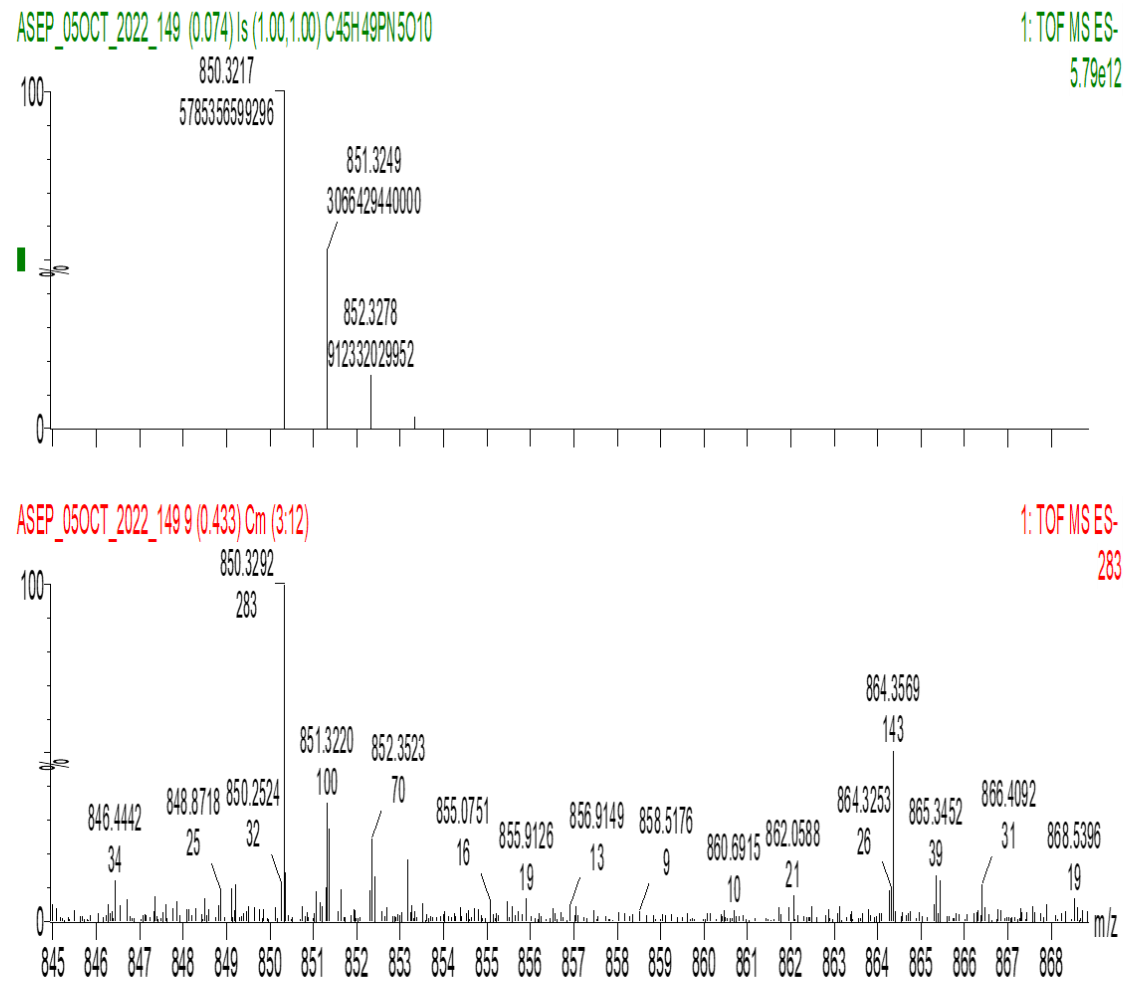
ASEP_05OCT_2022_149 (0.074) Is (1.00,1.00) C45H49PN5O10
1: TOF MS ES-
5.79e12
850.3217
5785356599296
851.3249
3066429440000
852.3278
912332029952
ASEP_05OCT_2022_149 9 (0.433) Cm (3:12)
1: TOF MS ES-
283
850.3292
283
851.3220
100
852.3523
70
846.4442
34
848.8718
25
850.2524
32
855.0751
16
855.9126
19
856.9149
13
858.5176
9
860.6915
10
862.0588
21
864.3253
26
864.3569
143
865.3452
39
866.4092
31
868.5396
19
m/z
%

c) i

ii

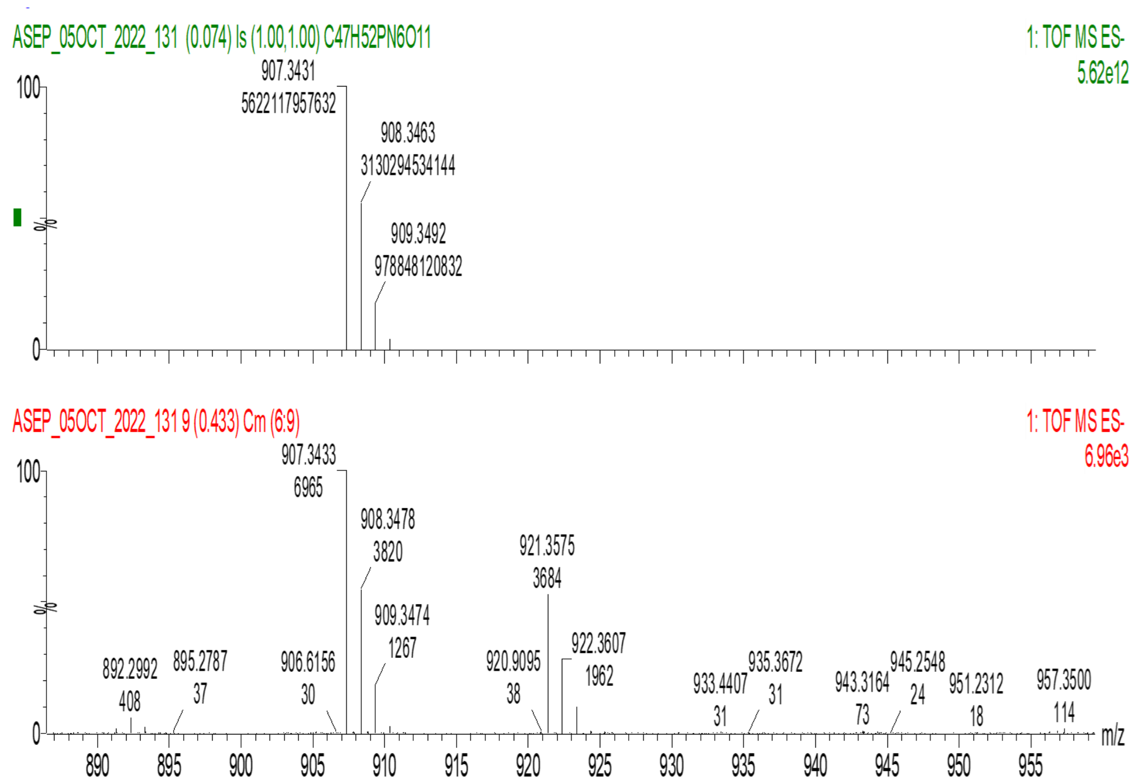
ASEP_05OCT_2022_131 (0.074) Is (1.00,1.00) C47H52PN6O11
1: TOF MS ES-
5.62e12
907.3431
5622117957632
908.3463
3130294534144
909.3492
978848120832
ASEP_05OCT_2022_131 9 (0.433) Cm (6:9)
1: TOF MS ES-
6.96e3
907.3433
6965
908.3478
3820
921.3575
3684
909.3474
1267
922.3607
1962
892.2992
408
895.2787
37
906.6156
30
920.9095
38
933.4407
31
935.3672
31
943.3164
73
945.2548
24
951.2312
18
957.3500
114
m/z
%

d) i

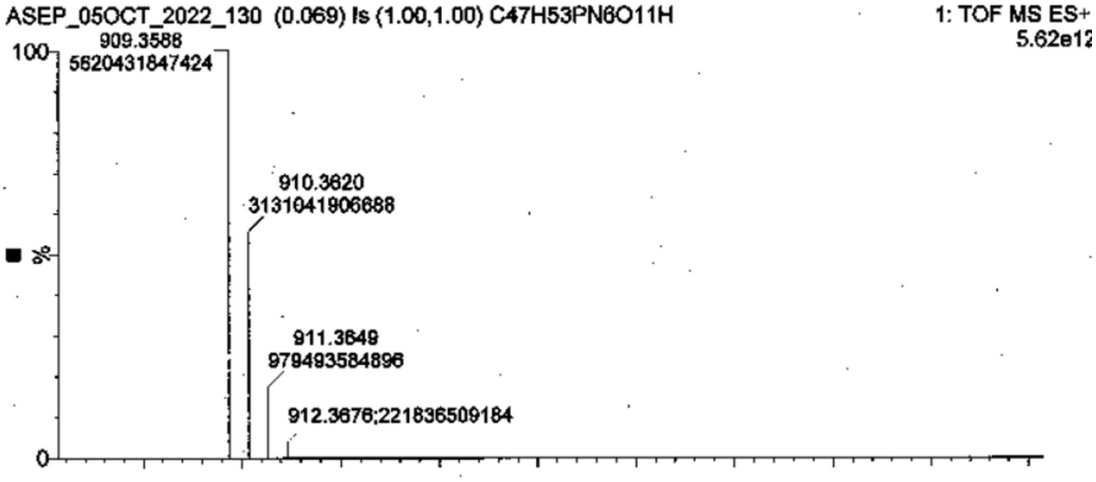
ASEP_05OCT_2022_130 (0.069) Is (1.00,1.00) C47H53PN6O11H
1: TOF MS ES+
5.62e12
100
909.3586
5620431847424
910.3620
3131041906688
911.3649
979493584896
912.3676;221836509184
%
0

ii

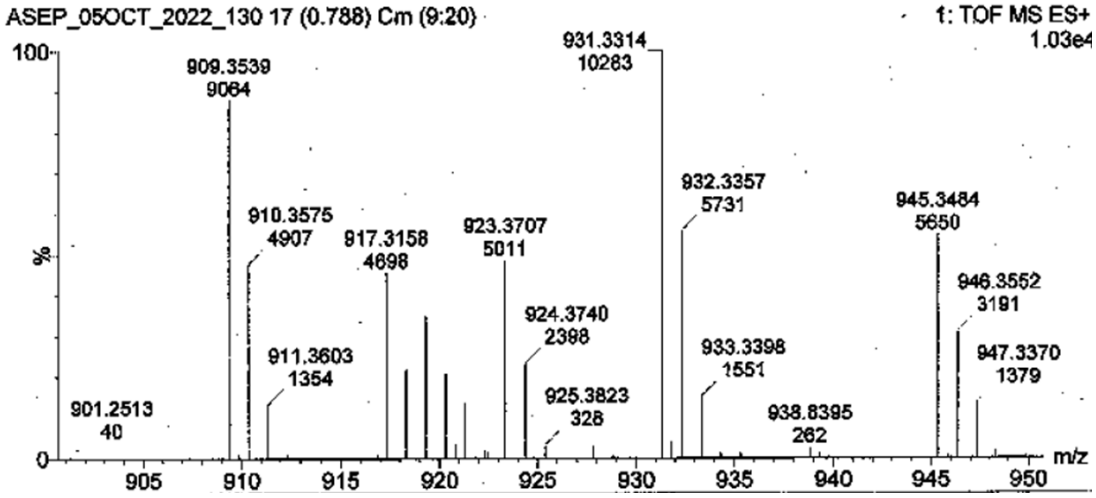
ASEP_05OCT_2022_130 17 (0.788) Cm (9:20)
1: TOF MS ES+
1.03e4
100
931.3314
10283
909.3539
9064
932.3357
5731
945.3484
5650
910.3575
4907
917.3158
4698
923.3707
5011
946.3552
3191
924.3740
2398
933.3398
1551
947.3370
1379
911.3603
1354
925.3823
328
901.2513
40
938.8395
262
%
0
905
910
915
920
925
930
935
940
945
950
m/z

e) **i**

ii

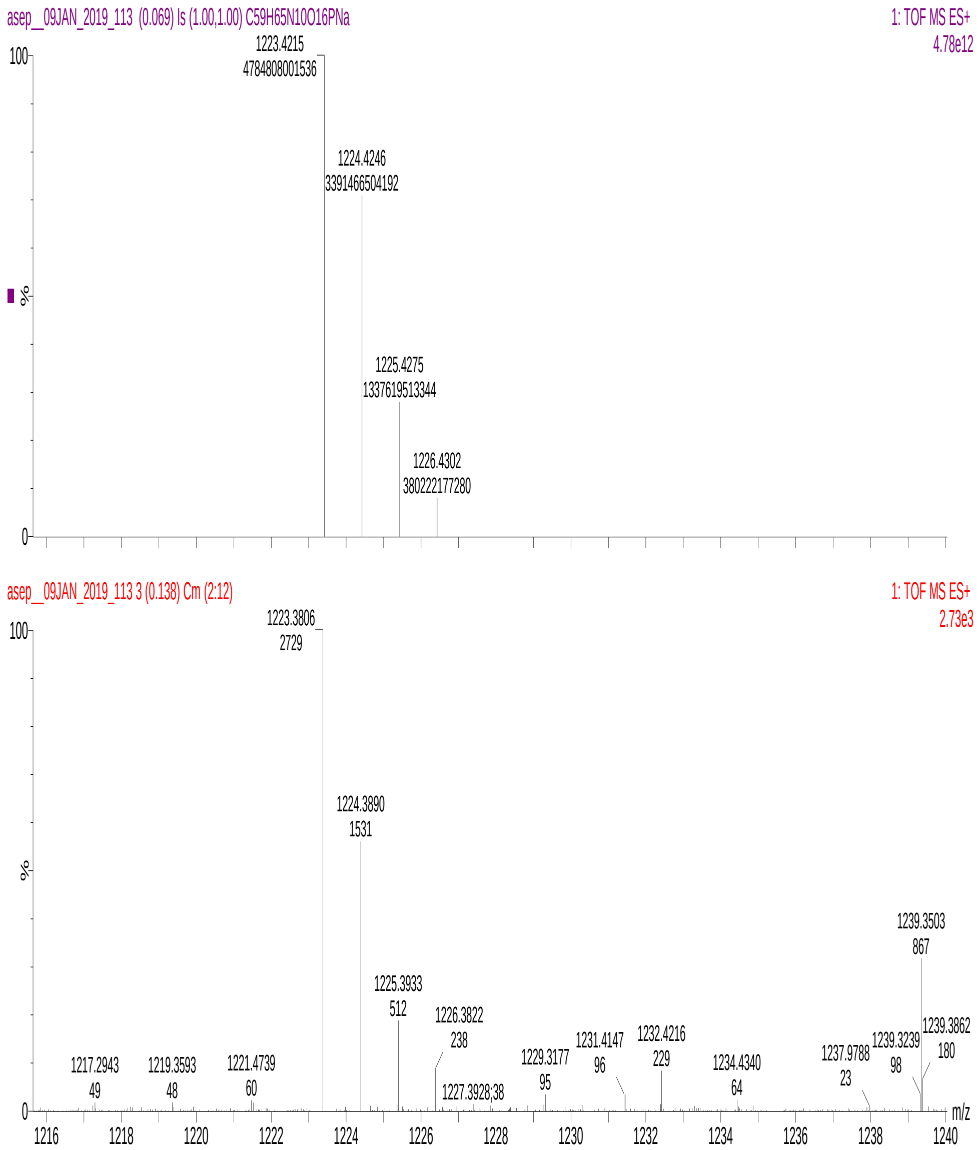

asep__09JAN_2019_113 (0.069) Is (1.00,1.00) C59H65N10O16PNa
1: TOF MS ES+
4.78e12
1223.4215
4784808001536
1224.4246
3391466504192
1225.4275
1337619513344
1226.4302
380222177280
100
%
0
asep__09JAN_2019_113 3 (0.138) Cm (2:12)
1: TOF MS ES+
2.73e3
1223.3806
2729
1224.3890
1531
1225.3933
512
1226.3822
238
1227.3928;38
1229.3177
95
1231.4147
96
1232.4216
229
1234.4340
64
1237.9788
23
1239.3239
98
1239.3503
867
1239.3862
180
1217.2943
49
1219.3593
48
1221.4739
60
m/z
1216
1218
1220
1222
1224
1226
1228
1230
1232
1234
1236
1238
1240

f) **i**

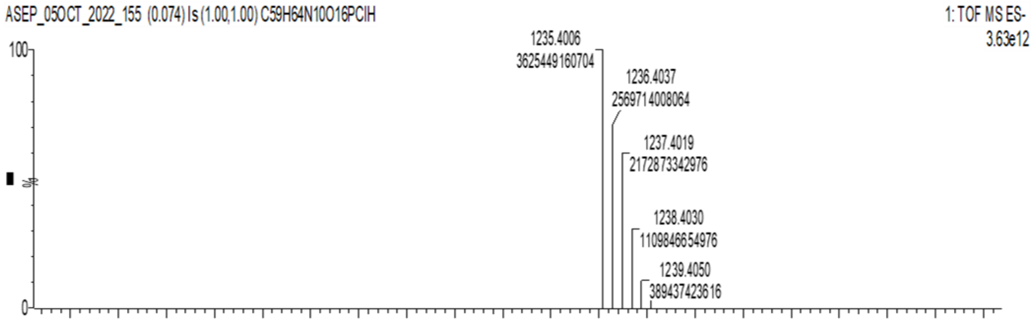
ASEP_05OCT_2022_155 (0.074) Is (1.00,1.00) C59H64N10O16PClH
1: TOF MS ES-
3.63e12
100
%
0
1235.4006
3625449160704
1236.4037
2569714008064
1237.4019
2172873342976
1238.4030
1109846654976
1239.4050
389437423616

g)

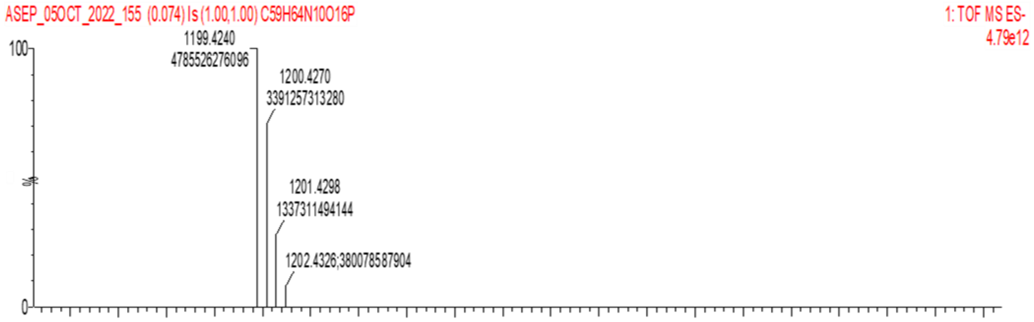
ASEP_05OCT_2022_155 (0.074) Is (1.00,1.00) C59H64N10O16P
1: TOF MS ES-
4.79e12
100
%
0
1199.4240
4785526276096
1200.4270
3391257313280
1201.4298
1337311494144
1202.4326;380078587904

ii

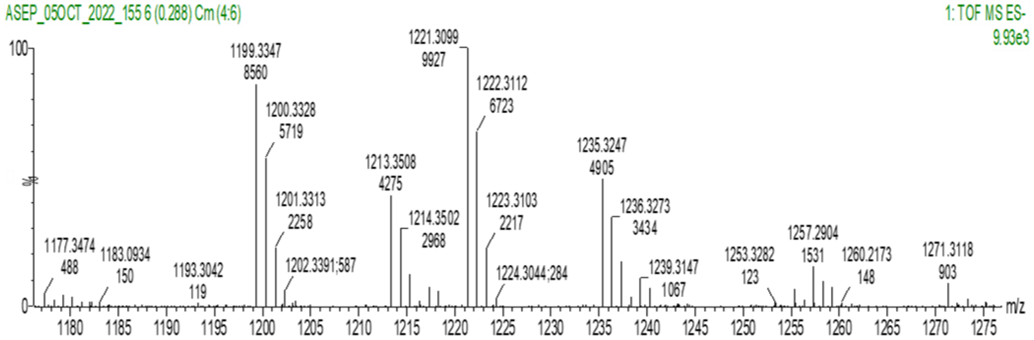
ASEP_05OCT_2022_155 6 (0.288) Cm (4:6)
1: TOF MS ES-
9.93e3
100
%
0
1177.3474
488
1183.0934
150
1193.3042
119
1199.3347
8560
1200.3328
5719
1201.3313
2258
1202.3391;587
1213.3508
4275
1214.3502
2968
1221.3099
9927
1222.3112
6723
1223.3103
2217
1224.3044;284
1235.3247
4905
1236.3273
3434
1239.3147
1067
1253.3282
123
1257.2904
1531
1260.2173
148
1271.3118
903
m/z
1180
1185
1190
1195
1200
1205
1210
1215
1220
1225
1230
1235
1240
1245
1250
1255
1260
1265
1270
1275

h) **i**

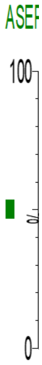
ASEF
100
%
0

ii

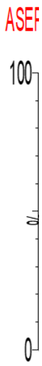
ASEF
100
%
0

Figure S3. Predicted theoretical ESI-MS determined using inbuilt Mass Lynx software by Waters LCT Premier (Waters, Hertfordshire, UK) (i) and observed (ii) ESI-MS traces for a) NapFFKY(p)-OH, b) NapffkY(p)-OH, c) NapFFKY(p)G-OH, d) NapffkY(p)G-OH, e) NapFFK(AZT)Y(p)-OH, f) Napffk(AZT)Y(p)-OH, g) NapFFK(AZT)Y(p)G-OH and h) Napffk(AZT)Y(p)G-OH.

a)

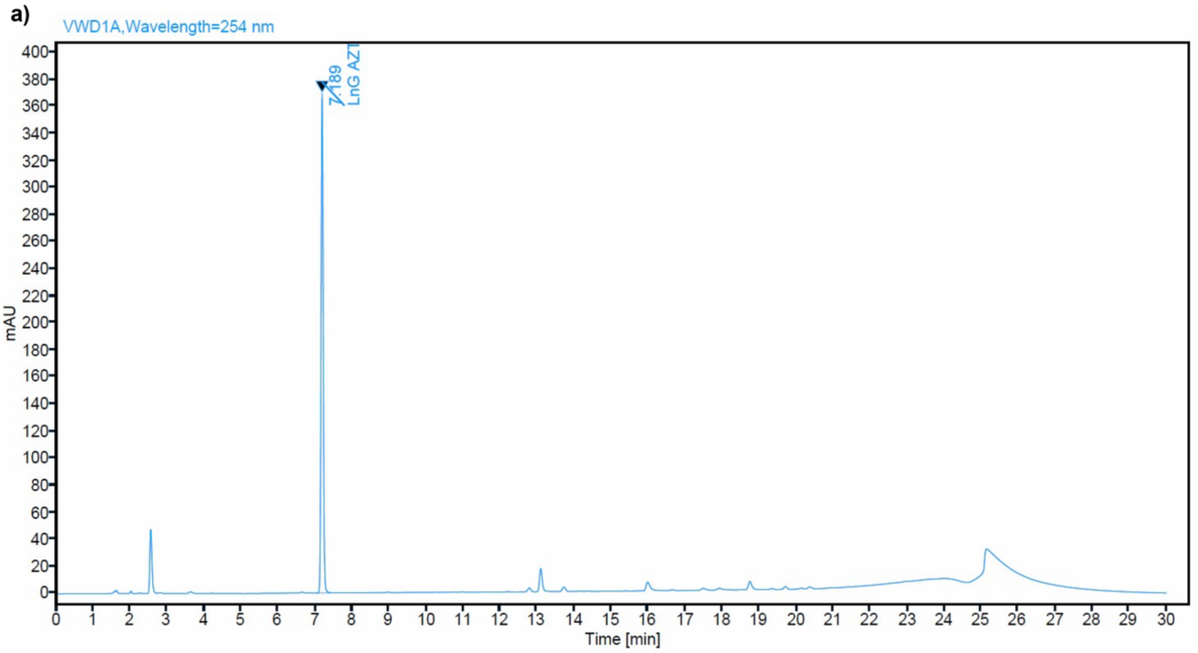

b)

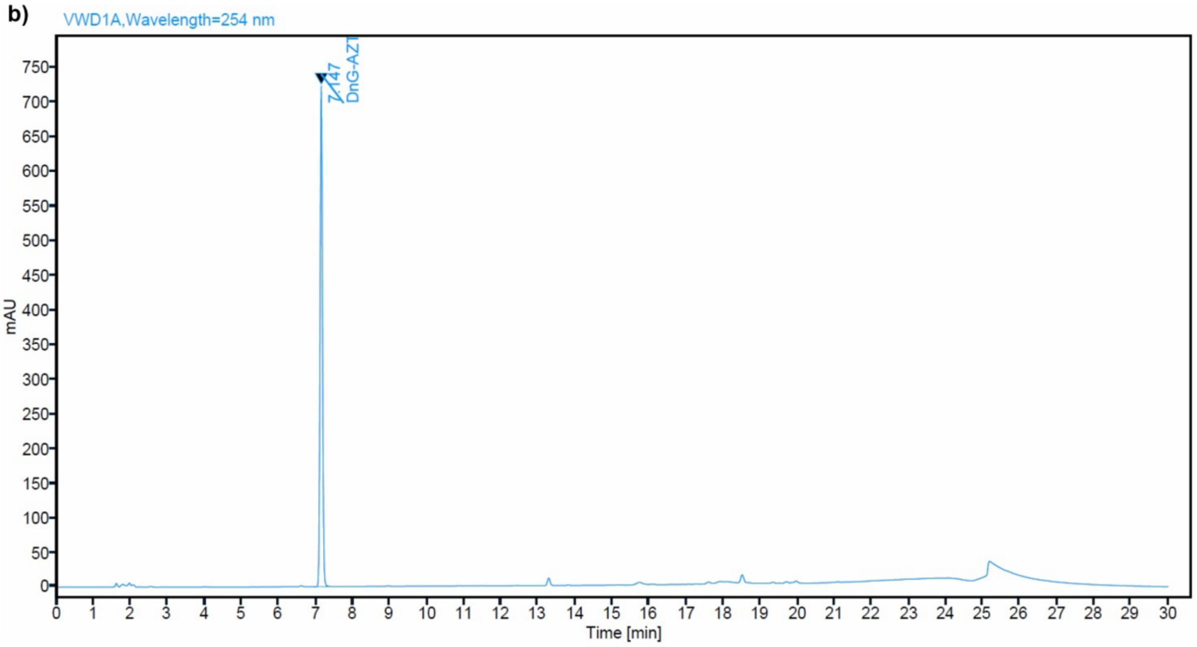

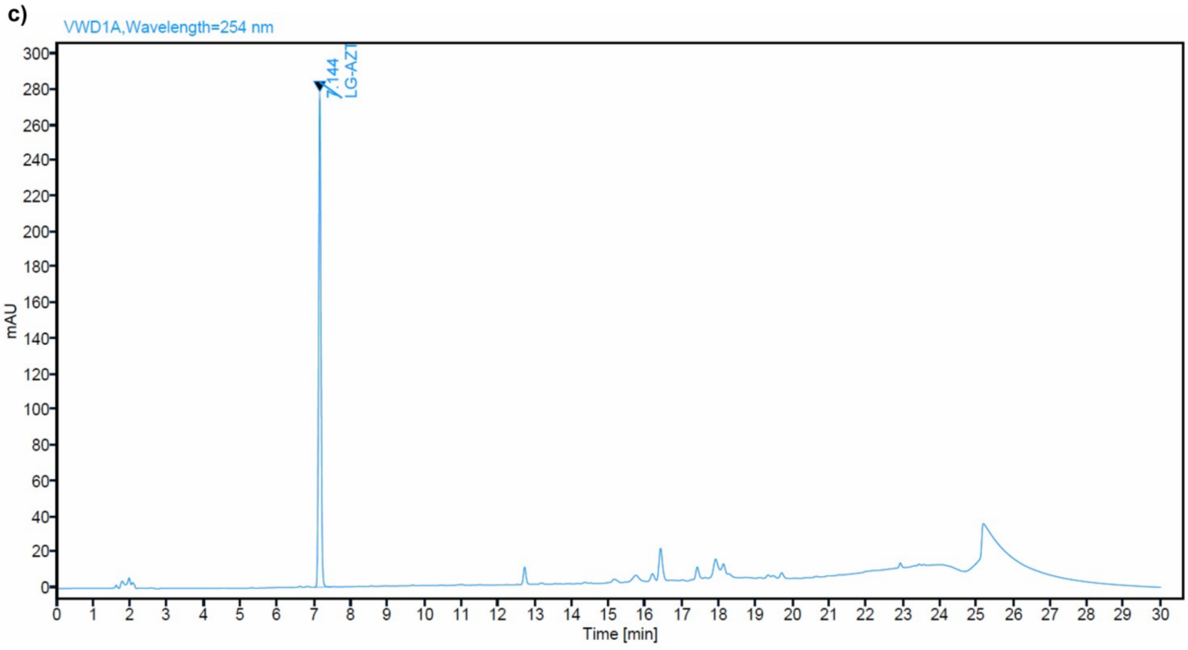

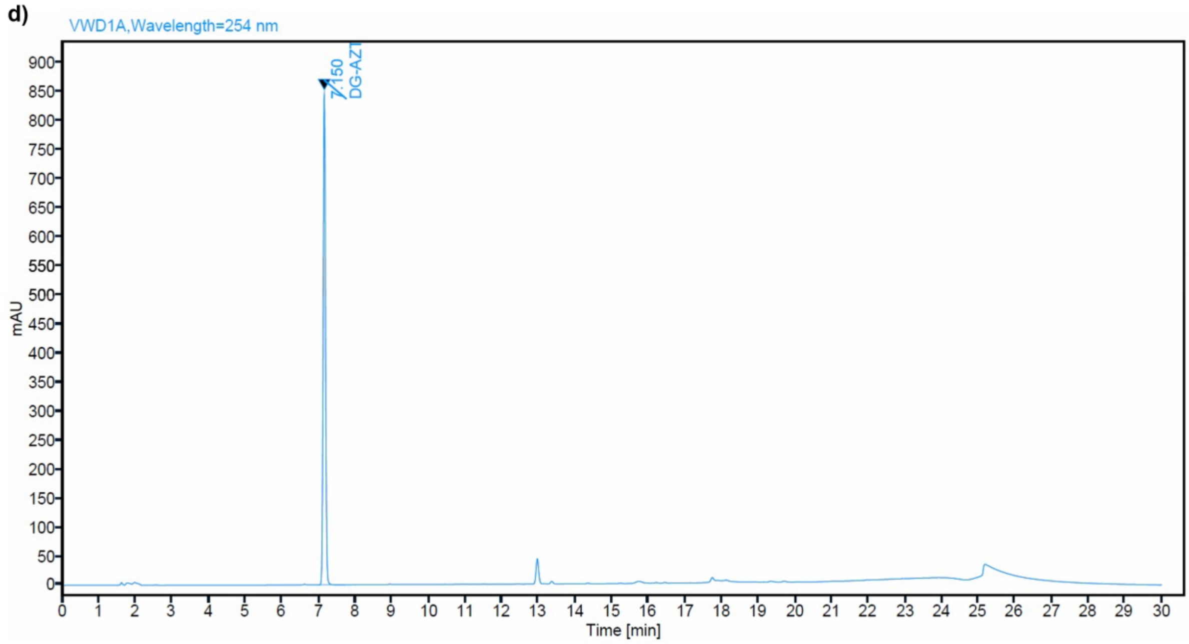

Figure S4. HPLC chromatograms for zidovudine conjugated peptides a) NapFFK(AZT)Y(p)-OH, b) Napffk(AZT)Y(p)-OH, c) NapFFK(AZT)Y(p)G-OH and d) Napffk(AZT)Y(p)G-OH.

Table S1. Peptides synthesized and studied and the molecular formula and weight of each. Electrospray mass spectrometry (ESI-MS) was utilized to confirm the synthesized peptide sequence. Critical gelation concentration (% w/v) elucidated by a vial inversion assay

Peptide and peptide-drug conjugate	Molecular formula	Molecular weight (Da)	ESI-MS Detected molecular weight (Da)	Critical gelation concentration (% w/v)
NapFFKY(p)-OH	$C_{45}H_{50}N_5O_{10}P$	851.88	852.34 (M+H$^+$)	1.5
NapffkY(p)-OH	$C_{45}H_{50}N_5O_{10}P$	851.88	850.33 (M-H$^+$).	1.5
NapFFKY(p)G-OH	$C_{47}H_{53}N_6O_{11}P$	908.93	907.34 (M-H$^+$)	0.5
NapffkY(p)G-OH	$C_{47}H_{53}N_6O_{11}P$	908.93	909.35 (M+H$^+$)	0.5
NapFFK(AZT)Y(p)-OH	$C_{59}H_{65}N_{10}O_{16}P$	1201.18	1223.38 (M+Na$^+$)	1.5
Napffk(AZT)Y(p)-OH	$C_{59}H_{65}N_{10}O_{16}P$	1201.18	1199.33 (M-H$^+$)	1.5
NapFFK(AZT)Y(p)G-OH	$C_{61}H_{68}N_{11}O_{17}P$	1258.23	1256.44 (M-H$^+$)	0.5
Napffk(AZT)Y(p)G-OH	$C_{61}H_{68}N_{11}O_{17}P$	1258.23	1256.45 (M-H$^+$)	0.5

Hydrogel formulation: The ability of peptides and peptide drug conjugates to form hydrogels in response to alkaline phosphatase was tested over a 0.1 – 2% w/v concentration range using the formulation steps outlined in Table S2.

Table S2. Stepwise formulation of a self-assembling enzyme-triggered gelator using 2% w/v peptide as an example (final volume 500 µL).

Formulation Step	Constituent	Quantity added
1	Peptide or peptide-drug	10 mg pre-weighed in HPLC vial
2	1.0 M NaOH	10 µL
3	PBS	200 µL[a)]
4	1.0 M NaOH	10 µL (according to pH, keep to 7.4)
5	PBS	200 µL[a)]
6	PBS	to final volume (500 µL)[a)]
7	Alkaline phosphatase	2 U (2 µL)[b)]

[a)] Sonicate (30 minutes) using a Branson 3510 sonic bath (Branson Ultrasonics Danbury, Connecticut, USA). Then the pH was monitored using a pH probe.

[b)] Overnight incubation at 37 °C.

^{1}H NMRs

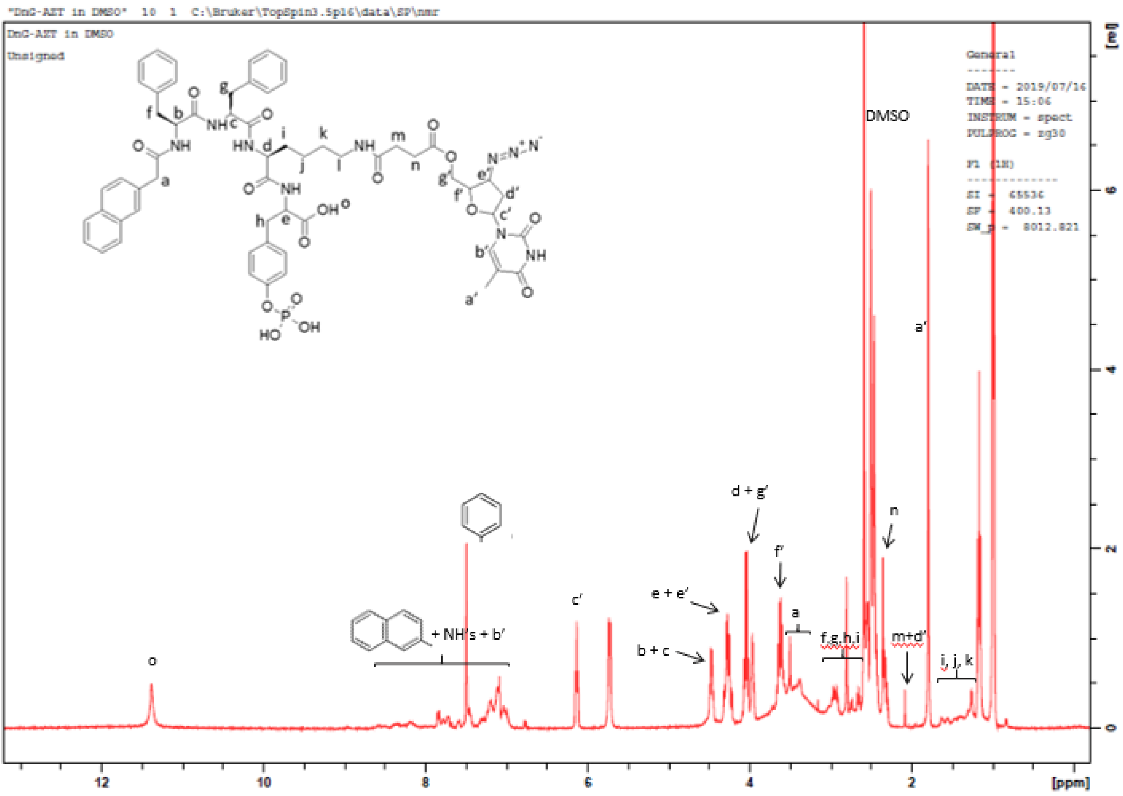

Figure S5. ^{1}H NMR trace assigned for Napffk(AZT)Y(p)-OH in DMSO-d_6

^{31}P NMRs

a)

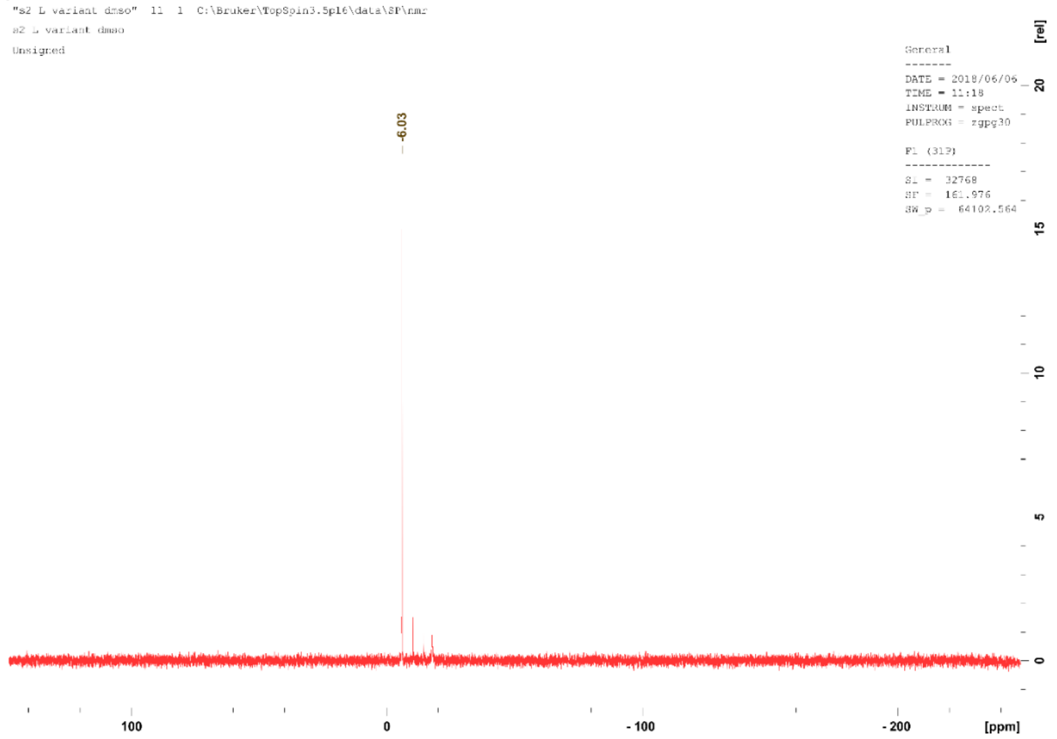

b)

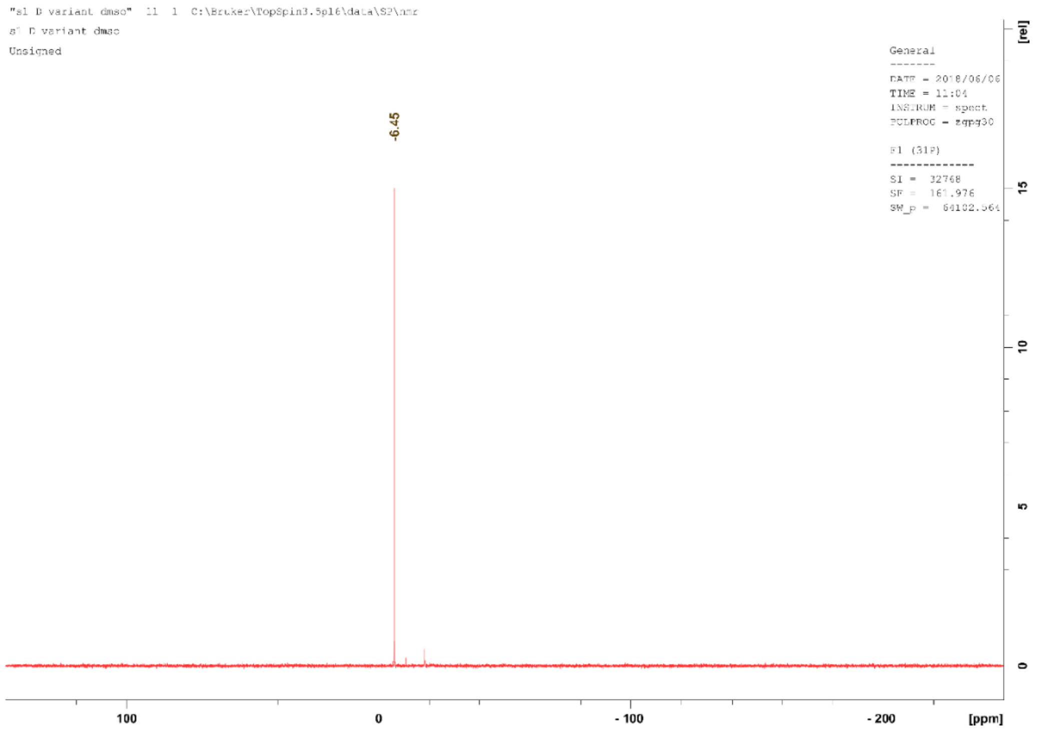

Figure S6. ^{31}P NMR trace for a) NapFFKY(p)G-OH and b) NapffkY(p)G-OH. Peaks at -6.03 and -6.45 demonstrate the presence and retention of phosphate grouping on the tyrosine motif, respectively.

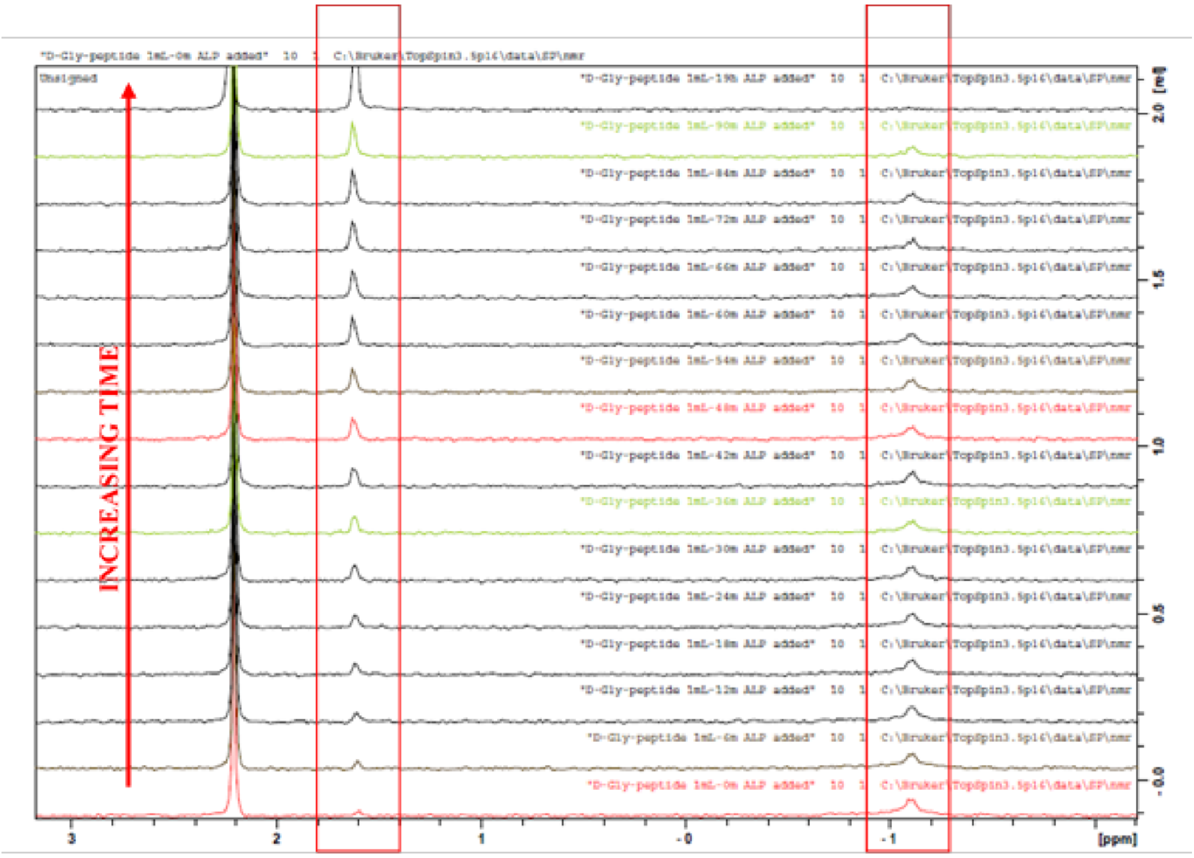

Figure S7. ^{31}P NMR trace for NapffkY(p)G-OH after the addition of 2 U of alkaline phosphatase measured every 6 minutes from t = 0 to t = 90 minutes and then again at t = 19 hours. The ^{31}P NMR trace confirms that the addition of alkaline phosphatase cleaved the phosphate group from the tyrosine residue to enable gelation to occur. It can be seen in box A (δ = -1.1 ppm) that the peak initially present after the addition of alkaline phosphatase decreases and has disappeared by the last trace indicating that this peak is the phosphate group on the tyrosine residue which is cleaved over time. The chemical shift is due to the rich electron density provided by the benzene ring and therefore shielding of the external magnetic field causing the peak to appear further upfield (to the right) in the spectrum. Box B (δ = 1.6 ppm) represents the free phosphate groups generated as a result of cleavage with the change in chemical shift as a result of a reduction in electron density due to cleavage from the sequence.

Vial inversion assays

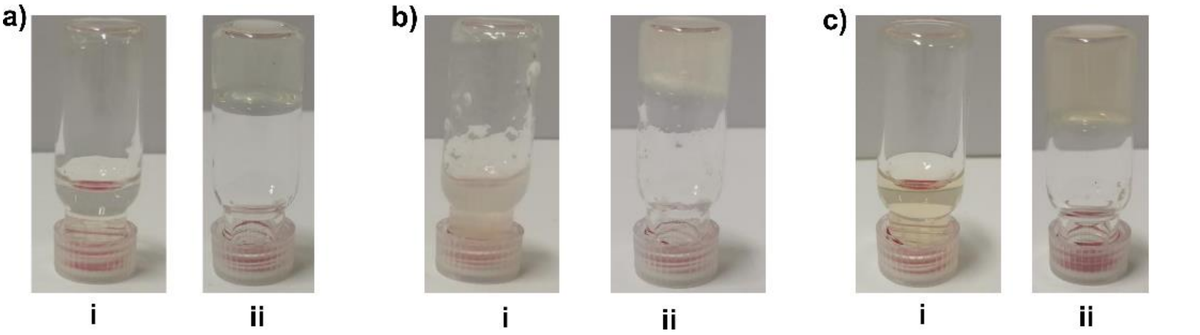

Figure S8a. Vial inversion assay for 2% w/v a) NapFFKY-OH, b) NapffkY-OH and c) NapFFKYG-OH. i) prior to 2 U alkaline phosphatase addition, ii) after 2 U alkaline phosphatase addition and formulation as outlined in Table S2.

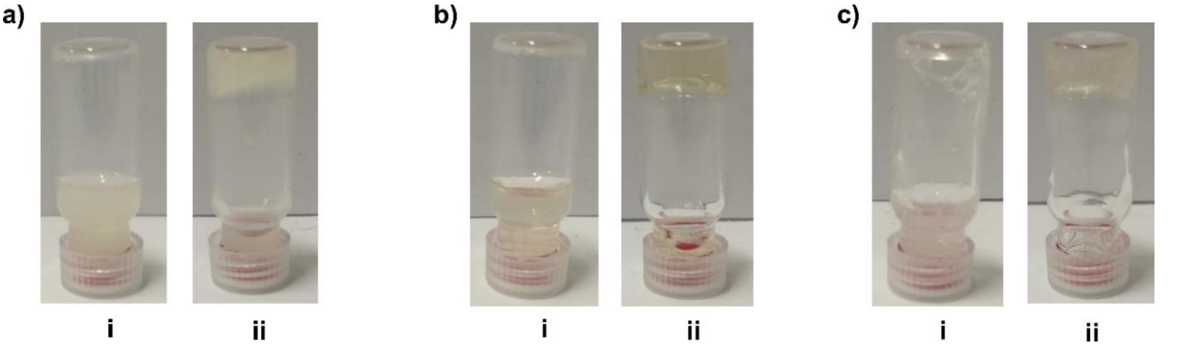

Figure S8b. Vial inversion assay for 2% w/v a) NapFFK(AZT)Y-OH, b) Napffk(AZT)Y-OH and c) NapFFK(AZT)YG-OH . i) prior to 2 U alkaline phosphatase addition, ii) after 2 U alkaline phosphatase addition and formulation as outlined in Table S2.

SEM

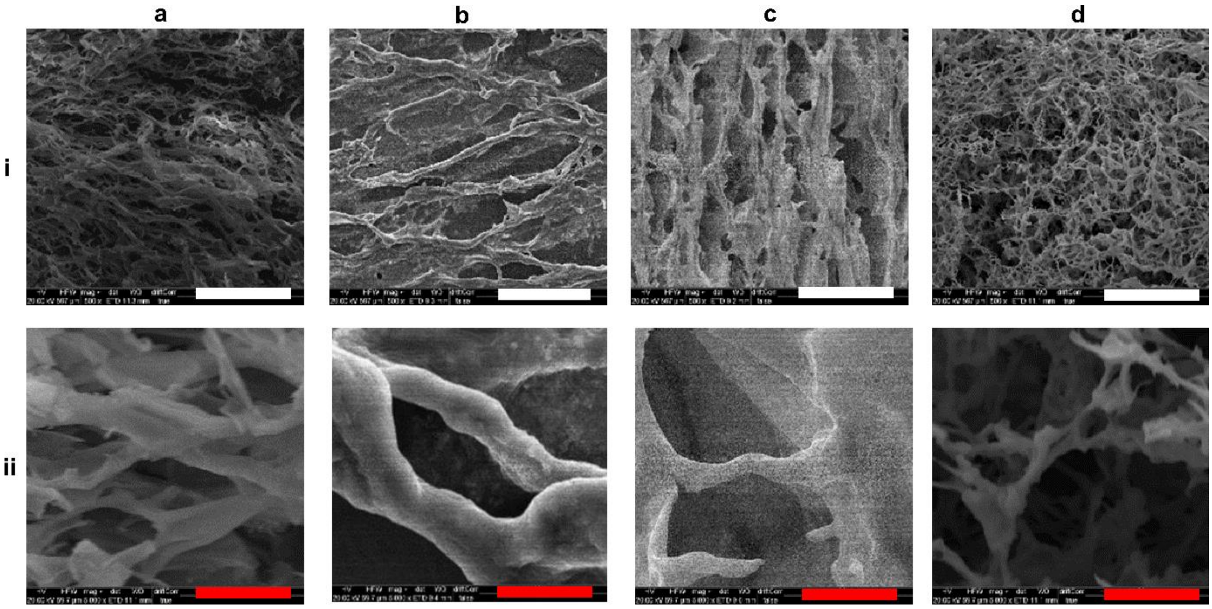

Figure S9. SEM images of 2.0% w/v gels: a) NapFFKYG-OH, b) NapffkYG-OH, c) NapFFKY-OH and d) NapffkY-OH at magnifications of (i) x500 (white scale bar = 200 μm) and (ii) x5000 (red scale bar = 20 μm).

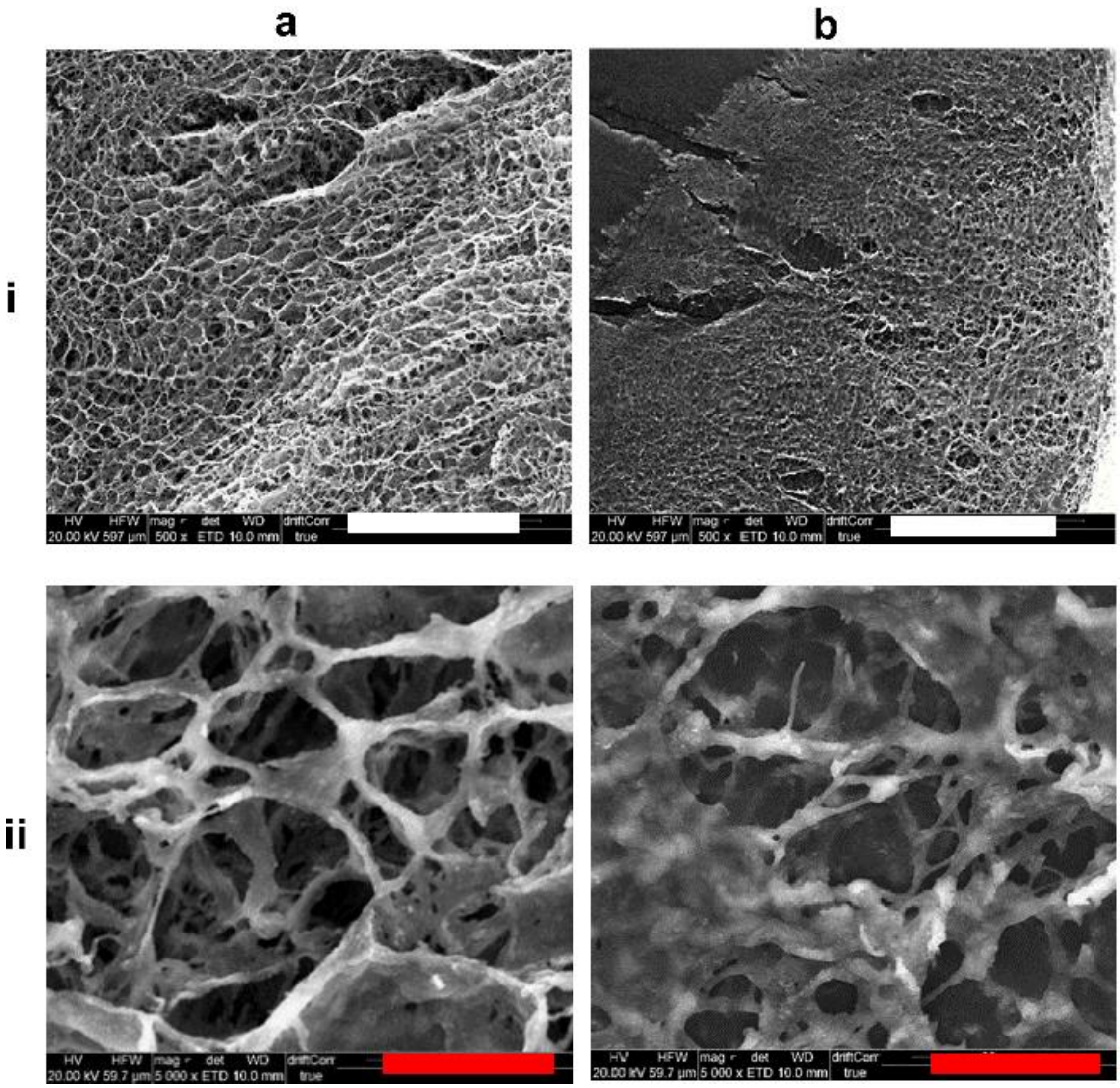

Figure S10. SEM images of 2.0% w/v drug conjugated gels: (a) NapFFK(AZT)Y-OH and (b) Napffk(AZT)YG-OH at magnifications of (i) x500 (white scale bar = 200 µm) and (ii) x5000 (red scale bar = 20 µm).

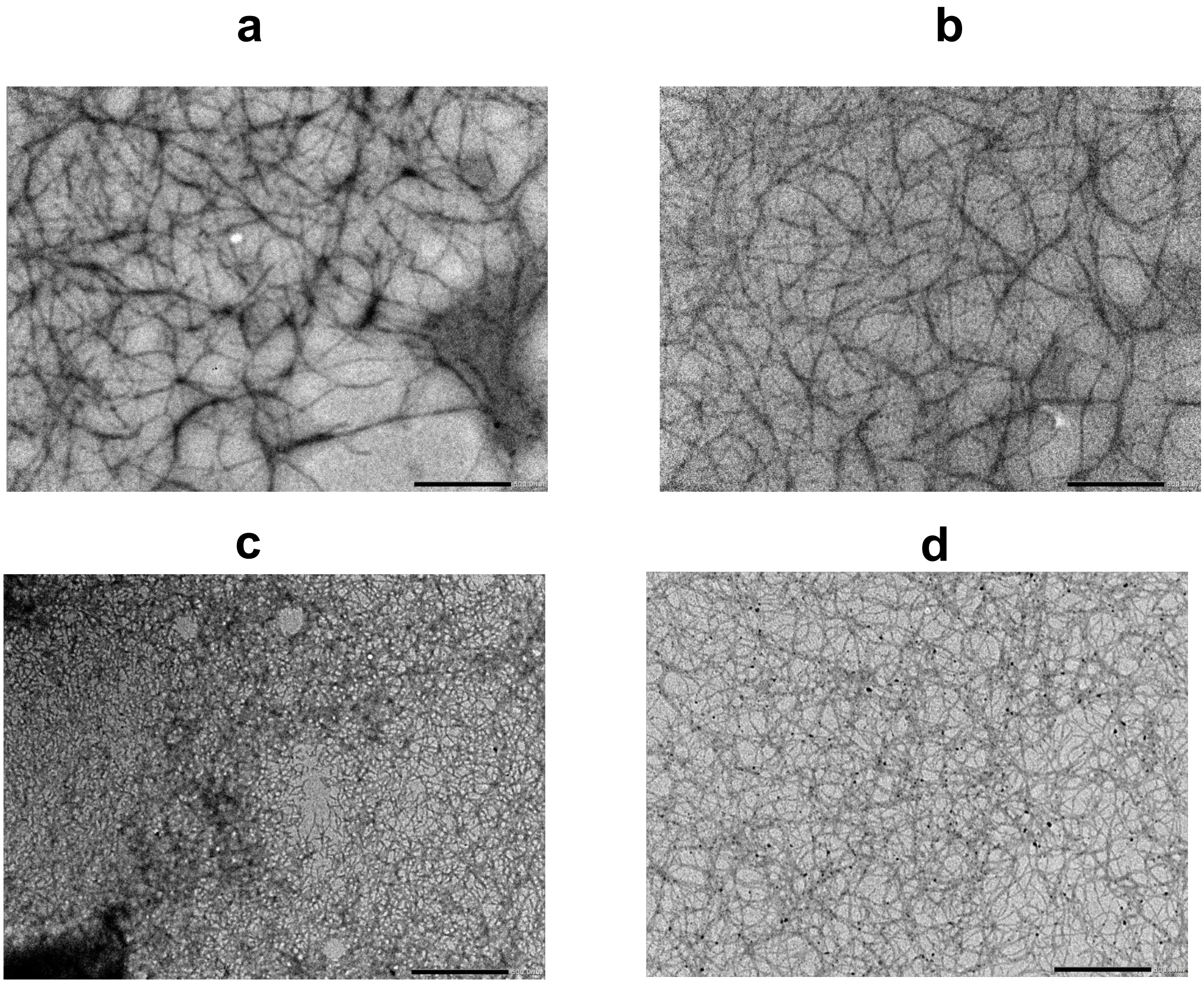

Figure S11. TEM images of 2.0% w/v gels formulated from: a) NapFFKYG-OH, b) NapffkYG-OH, c) NapFFKY-OH and d) NapffkY-OH at a magnification of x20,000 (black scale bar = 500 nm).

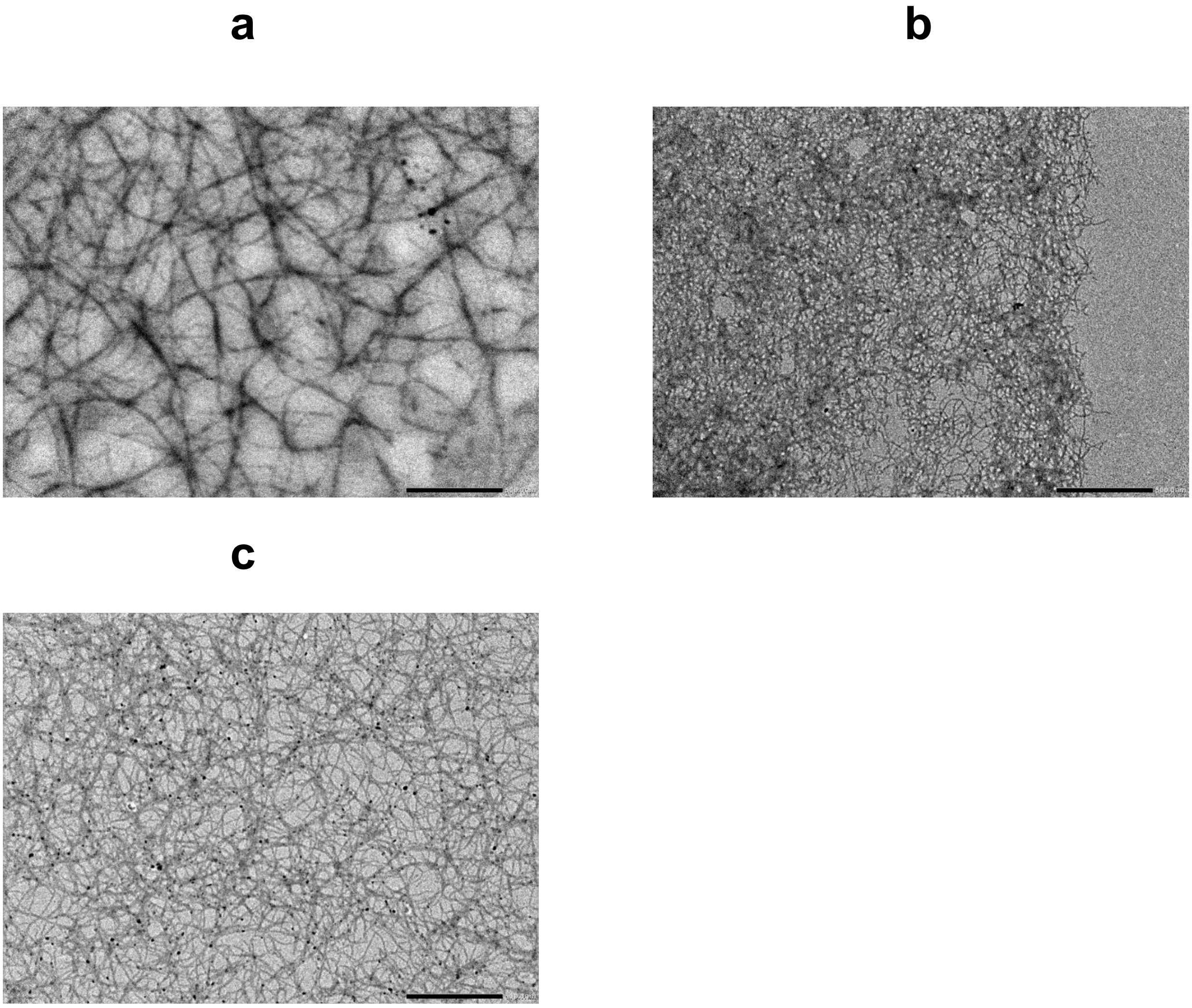

Figure S12. TEM images of 2.0% w/v gels formulated from: a) NapFFK(AZT)YG-OH, b) NapFFK(AZT)Y-OH and c) Napffk(AZT)Y-OH at a magnification of x20,000 (black scale bar = 500 nm).

CD Spectroscopy

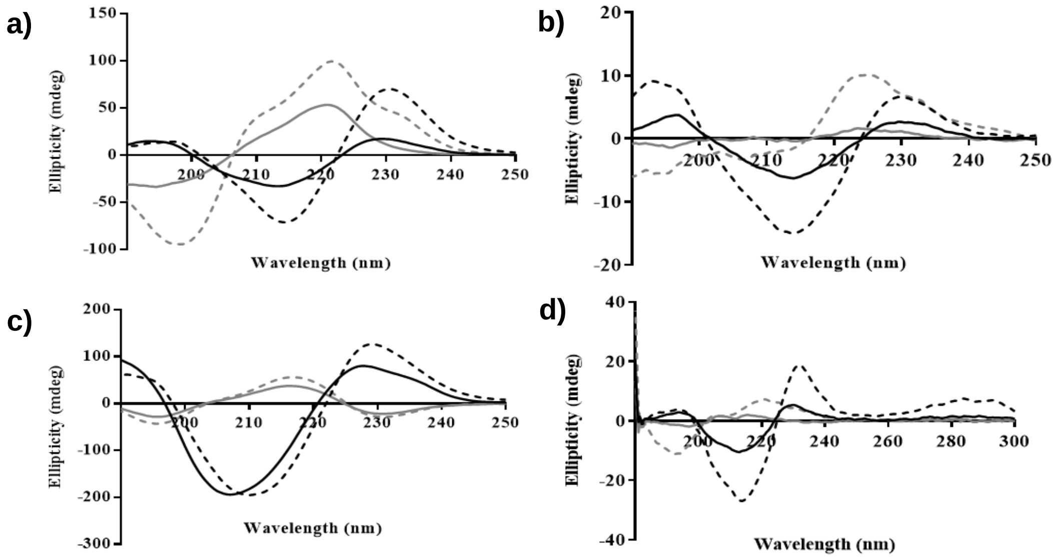

Figure S13. CD spectra obtained for a) NapFFKYG-OH (black solid and dashed lines, HT values 265 – 434 V) and for NapffkYG-OH (gray solid and dashed lines, HT values 267 – 440 V), b) NapFFK(AZT)YG-OH (black solid and dashed lines, HT values 262 – 324 V) and for Napffk(AZT)YG-OH (gray solid and dashed lines, HT values 266 – 307 V), c) NapFFKY-OH (black solid and dashed lines, HT values 272 – 566 V) and for NapffkY-OH (gray solid and dashed lines, HT values 264 – 412 V) and d) NapFFK(AZT)Y-OH (black solid and dashed lines, HT values 254 – 552 V) and for Napffk(AZT)Y-OH (gray solid and dashed lines, HT values 255 – 603 V) at concentrations of 0.5 and 2.0% w/v. Solid lines represent 0.5% w/v concentrations and dashed lines represent 2.0% w/v concentrations.

Rheology

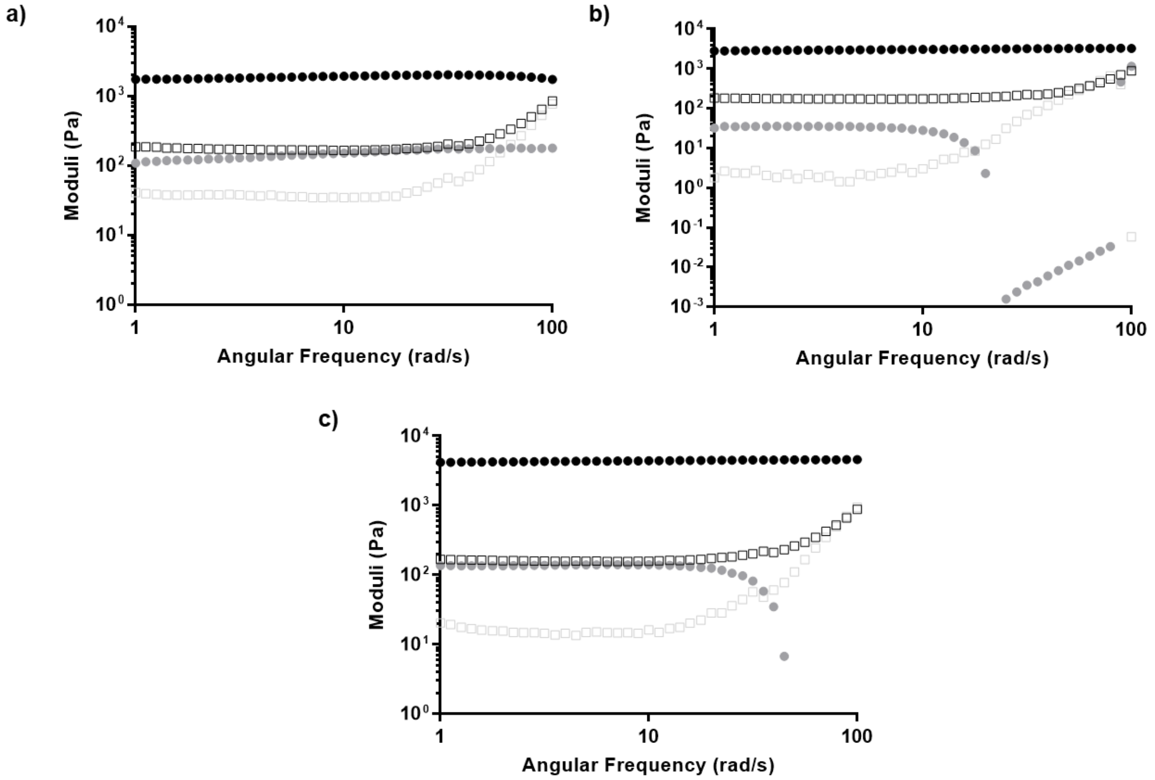

Figure S14. Frequency sweeps measured using an Anton Paar 302 rheometer with a vane and cup geometry on 2% w/v formulated peptide gels. a) NapFFKYG-OH and NapFFK(AZT)YG-OH, b) NapFFKY-OH and NapFFK(AZT)Y-OH and c) NapffkY-OH and Napffk(AZT)Y-OH. In all cases the parent compound is presented in black and the zidovudine conjugate is presented in gray. Filled circles represent G′, open squares represent G″.

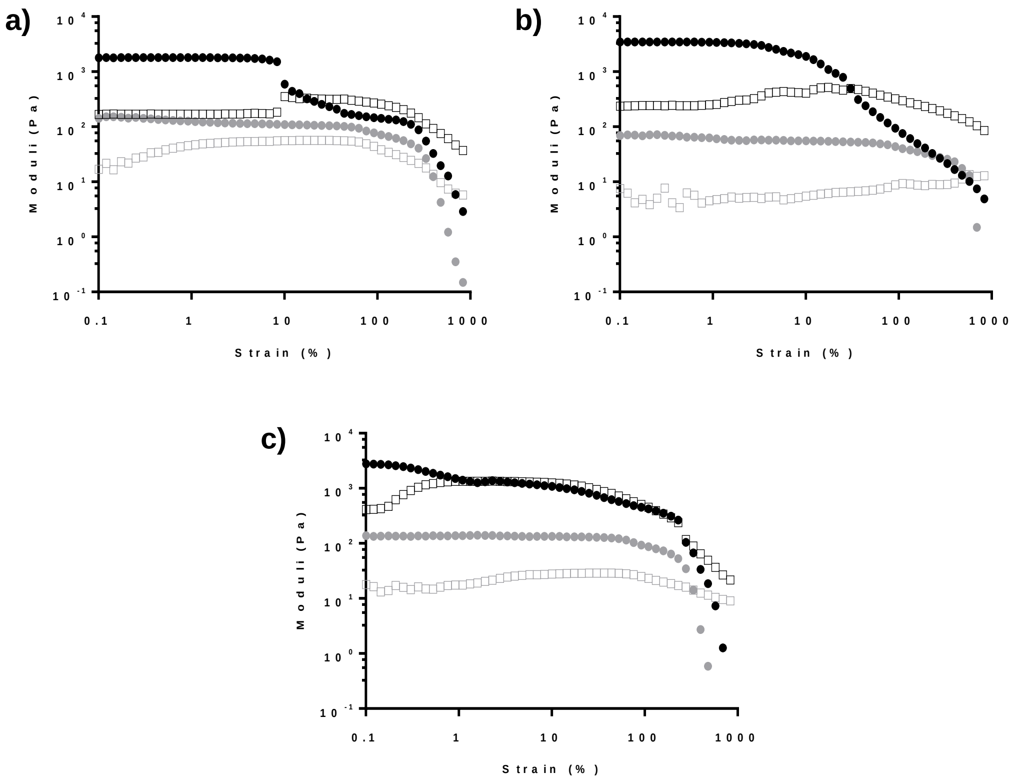

Figure S15. Strain sweeps measured using an Anton Paar 302 rheometer with a vane and cup geometry on 2% w/v formulated peptide gels. a) NapFFKYG-OH and NapFFK(AZT)YG-OH, b) NapFFKY-OH and NapFFK(AZT)Y-OH and c) NapffkY-OH and Napffk(AZT)Y-OH. In all cases the parent compound is presented in black and the zidovudine conjugate is presented in gray. Filled circles represent G′, open squares represent G″.

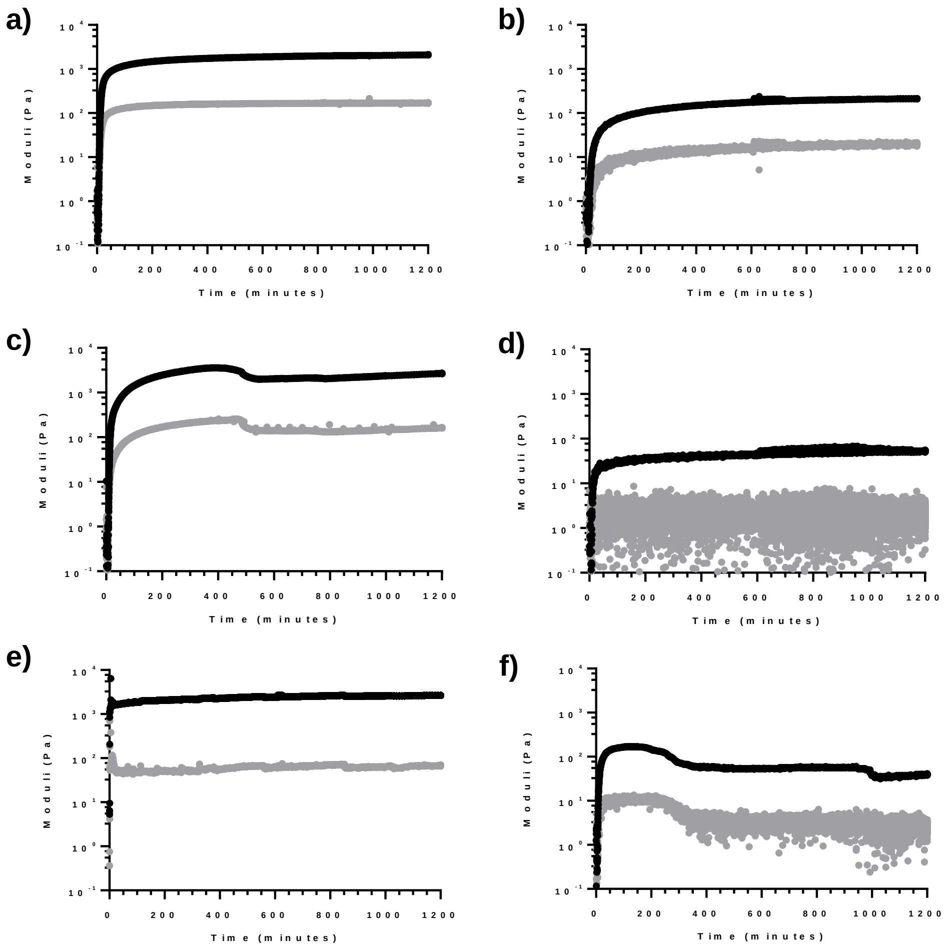

Figure S16. Time sweeps measured using an Anton Paar 302 rheometer with a sand blasted parallel plate PP50/S system (37 °C, 0.5 mm measuring gap) on 2% w/v formulated peptide gels. a) NapFFKYG-OH, b) NapFFK(AZT)YG-OH, c) NapFFKY-OH, d) NapFFK(AZT)Y-OH, e) NapffkY-OH and f) Napffk(AZT)Y-OH. Black lines represent G′, gray lines represent G″.

Table S3. Gelation times for peptide hydrogels upon addition of alkaline phosphatase enzyme derived from data above in Figure S12 and Figure 3 c-d.

Peptide	G′ and G″ cross	G′ > 2x G″	Time for stable G′
a) NapFFKYG-OH	5.92 min	7.75 min	65.7 min (10^3)
b) NapFFK(AZT)YG-OH	12.8 min	13.5 min	185 min (10^2)
c) NapFFKY-OH	8.58 min	9.67 min	68.3 min (10^3)
d) NapFFK(AZT)Y-OH	8.5 min	9 min	88.3 min ($10^{1.5}$)
e) NapffkYG-OH	0.75 min (45 s)	0.917 min (55 s)	46.4 min (10^3)
f) Napffk(AZT)YG-OH	27 min	30.5 min	90.3 min(10^2)
g) NapffkY-OH	0.167 min (10 s)	0.333 min (20 s)	1.17 min (70 s) (10^3)
h) Napffk(AZT)Y-OH	0.833 min (50 s)	5.83 min	28 min (10^2)

SANS

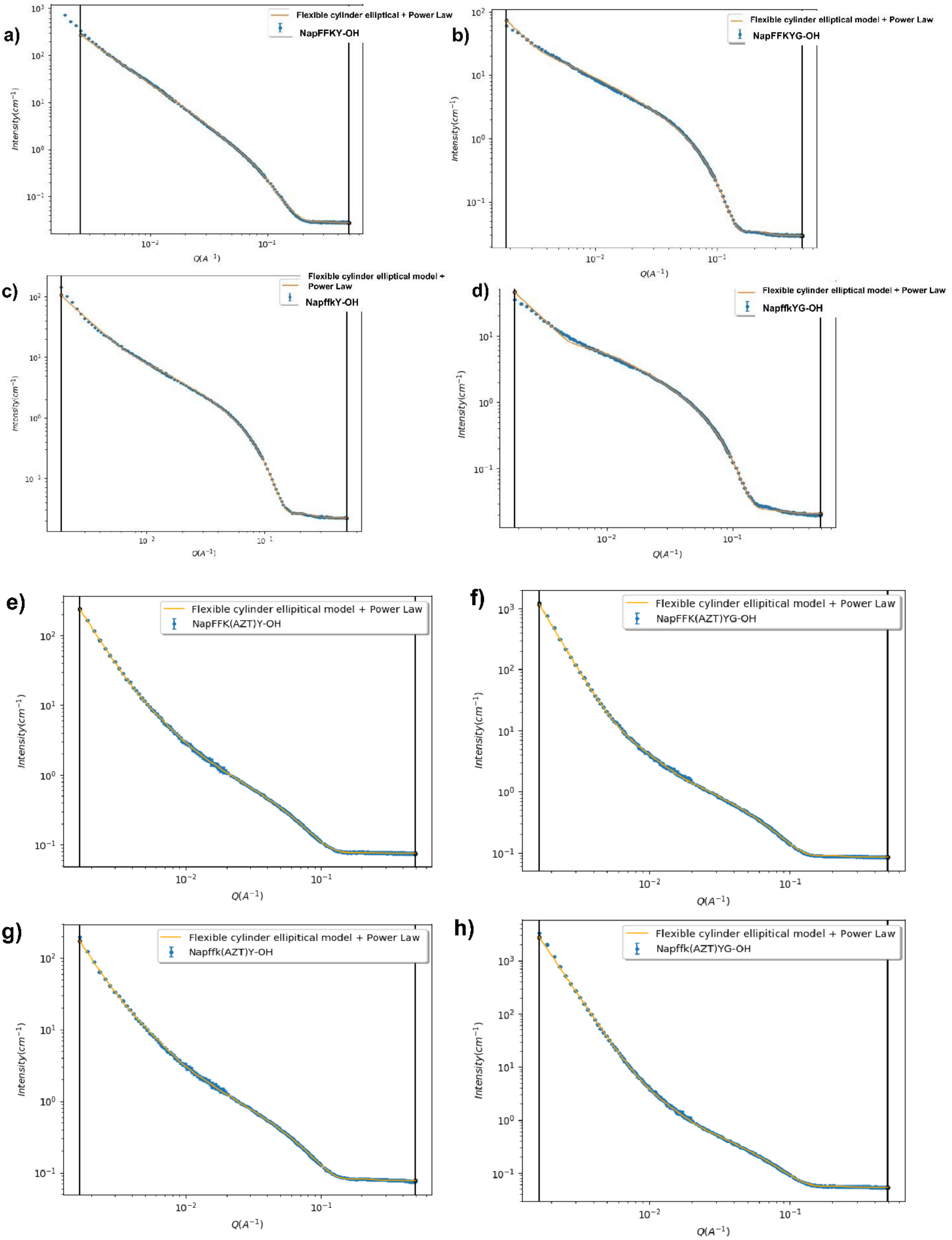

Figure S17. SANS data for 2% w/v peptide hydrogels a) NapFFKY-OH, b) NapFFKYG-OH, c) NapffkY-OH, d) NapffkYG-OH, e) NapFFK(AZT)Y-OH, f)

NapFFK(AZT)YG-OH, g) Napffk(AZT)Y-OH and h) Napffk(AZT)YG-OH (dotted line). The solid line relates to the model data for the flexible cylinder elliptical model. Scattering data collected over a wide Q range [Q = 4πsin(θ/2)/λ] of 0.001 to 0.5 $Å^{-1}$ and three sample-detector distances (1.4 m, 8 m, 39 m).

Table S4. Summary of the SANS fitting parameters.

**Parameter \Peptide hydrogel (2% w/v)**	**NapFFKY-OH**	**NapFFKYG-OH**	**NapffkY-OH**	**NapffkYG-OH**	**NapFFK(AZT)Y-OH**	**NapFFK(AZT)YG-OH**	**Napffk(AZT)Y-OH**	**Napffk(AZT)YG-OH**
Scale	0.0046856E-5 +/- 1.2111	0.0059716 E-5 +/- 1.5383	0.0037313E-5 +/- 1.1722	0.0054887E-5 +/- 1.5409	7.6759E-07 +/- 16.451	2.89E-08 +/- 0.59263	3.60E-08 +/- 0.24386	2.02E-07 +/- 6.8836
Background (cm^{-1})	0.027338 +/- 3.0788e-5	0.029379 +/- 2.9467e-5	0.020866 +/- 2.896e-5	0.02208 +/- 2.911e-5	0.075657 +/- 2.75E-05	0.083826 +/- 2.88E-05	0.076342+/- 2.78E-05	0.05353 +/- 2.24E-05
Length (Å)	75680 +/- 11099	2.9899e+15 +/- 3.3481e+15	7.0584e+43 +/- 6.6517e+43	1.8917e+5 +/- 33290	752.55 +/- 50.063	2863.7 +/- 1.00E+08	2368.6 +/- 1.00E+08	1466.2 +/- 1.00E+08
Khun length (Å)	75.838 +/- 0.38383	1001.5 +/- 9.9707	670.27 +/- 3.9572	560.66 +/- 3.0707	494.45 +/- 20.793	3.39E-21 +/- 2.03E+06	8.27E-25 +/- 2.82E+06	3.63E-17 +/- 8.90E-08
Radius (Å)	14.954 +/- 0.020758	20.378 +/- 0.028828	19.393 +/- 0.035733	19.585 +/- 0.030648	19.977 +/- 0.11472	22.787 +/- 0.022169	22.607 +/- 0.02427	21.52 +/- 0.02728
Axis ratio	2.1361 +/- 0.0068177	1.454 +/- 0.0041078	1.5634 +/- 0.0055837	1.4657 +/- 0.004574	1.4721 +/- 0.016232	9.8719 +/- 0.11499	17.872 +/- 0.37564	7.8643 +/- 0.083226
SLD ($x10^{-6}$ $Å^{-2}$)	2.143	2.143	2.143	2.143	2.143	2.143	2.143	2.143
SLD solvent ($x10^{-6}$ $Å^{-2}$)	6.39	6.39	6.39	6.39	6.39	6.39	6.39	6.39
$χ^2$	18.758	19.805	13.397	12.027	2.8005	6.309	7.4149	5.2904

Cell cytotoxicity/biocompatibility

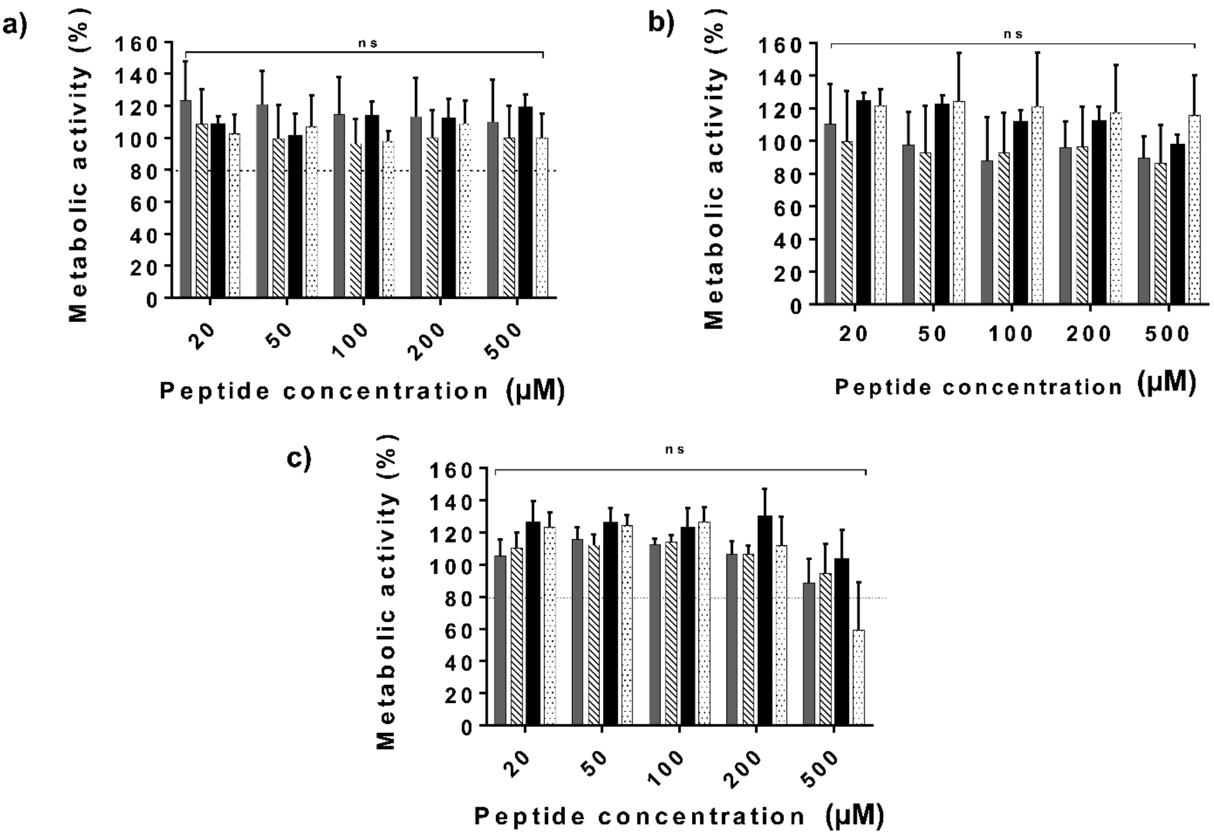

Figure S18. Cell cytotoxicity/metabolic activity of fully solubilized parent peptides i.e. not conjugated to AZT (µM range) using an MTS viability assay after a) 6 b) 24 and c) 72 hours. Means ± SD provided for nine replicates. Key: NapFFKY(p)-OH: gray, NapffkY(p)-OH: dashed, NapFFKY(p)G-OH: black, NapffkY(p)G-OH: dotted. NS: no significant ($p > 0.05$), *$p \leq 0.05$, **$p < 0.01$, ***$p < 0.001$, ****$p < 0.0001$ difference between the peptides and the negative control (media only).

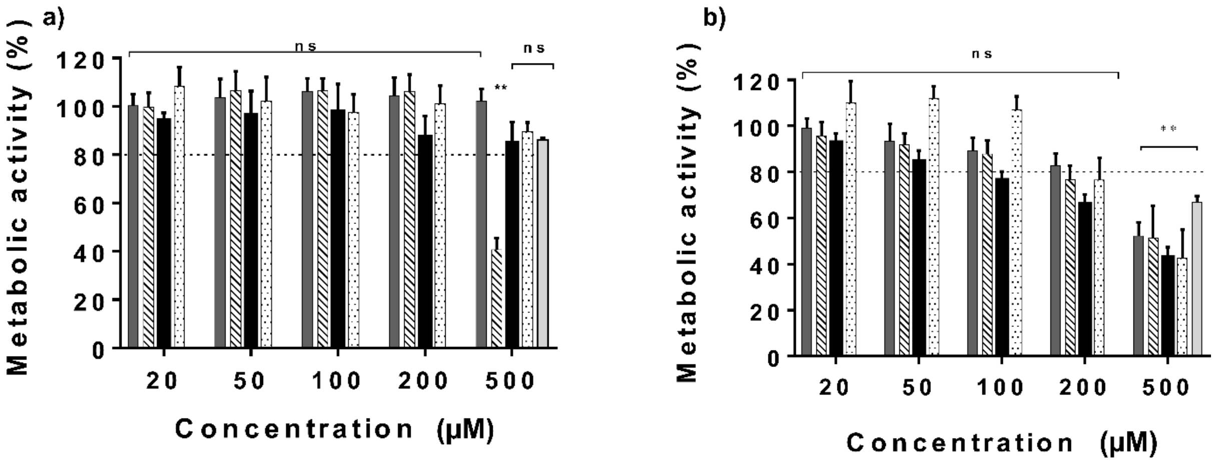

Figure S19. Cell cytotoxicity/metabolic activity of fully solubilized zidovudine conjugated peptides (μM range) using an MTS viability assay after a) 6 and b) 72 hours. Means ± SD provided for nine replicates. Key: NapFFK(AZT)Y(p)-OH: dark gray, Napffk(AZT)Y(p)-OH: dashed, NapFFK(AZT)Y(p)G-OH: black, Napffk(AZT)Y(p)G-OH: dotted, 500 μM zidovudine drug only (light gray). NS: no significant ($p > 0.05$), *$p \leq 0.05$, **$p < 0.01$, ***$p < 0.001$, ****$p < 0.0001$ difference between the peptides and the negative control (media only).

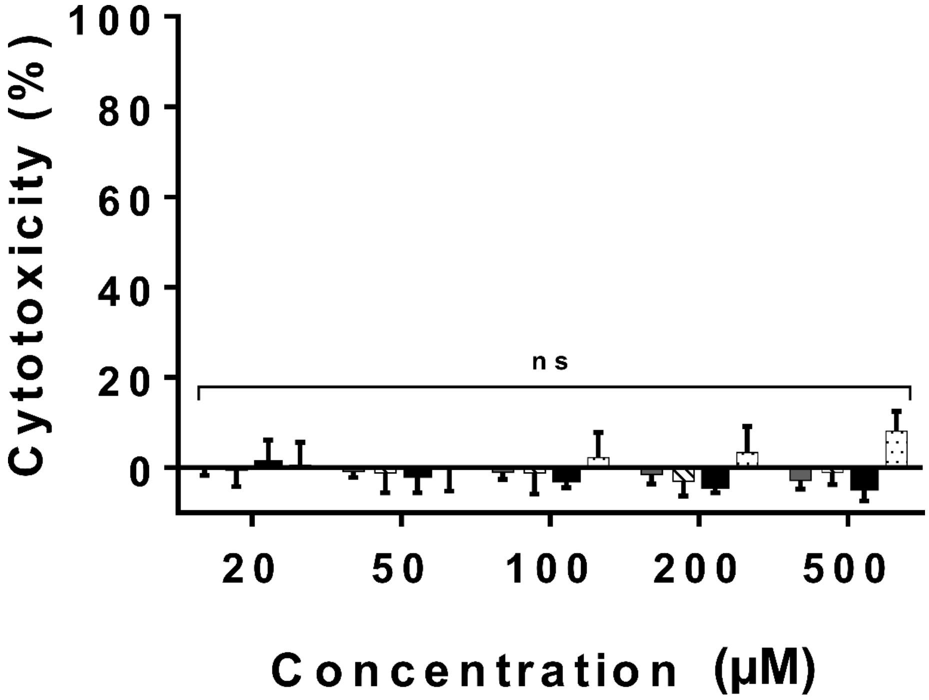

Figure S20. Cell cytotoxicity of fully solubilized peptides (μM range) using an LDH cytotoxicity assay after 6 hours. Means ± SD provided for nine replicates. Key: NapFFKY(p)-OH: gray, NapffkY(p)-OH: dashed, NapFFKY(p)G-OH: black, NapffkY(p)G-OH: dotted. NS: no significant ($p > 0.05$), *$p \leq 0.05$, **$p < 0.01$, ***p <

0.001, ****p < 0.0001 difference between the peptides and the negative control (media only).

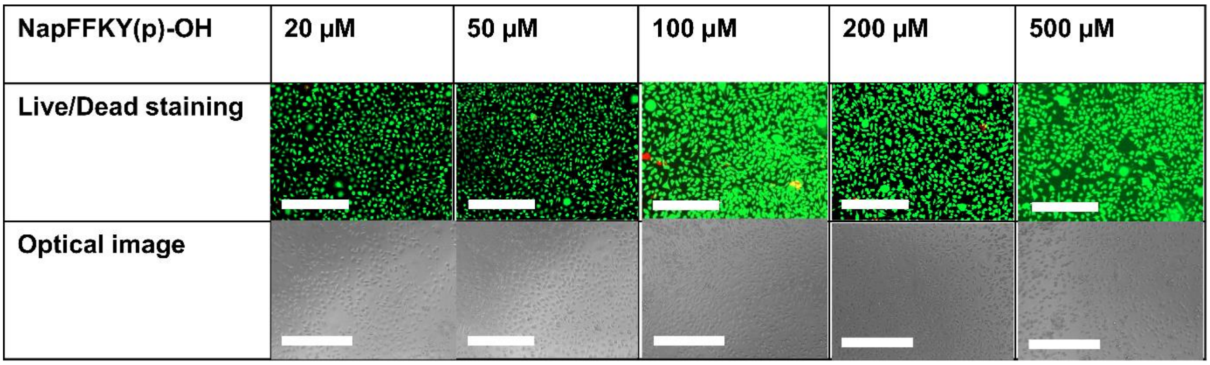

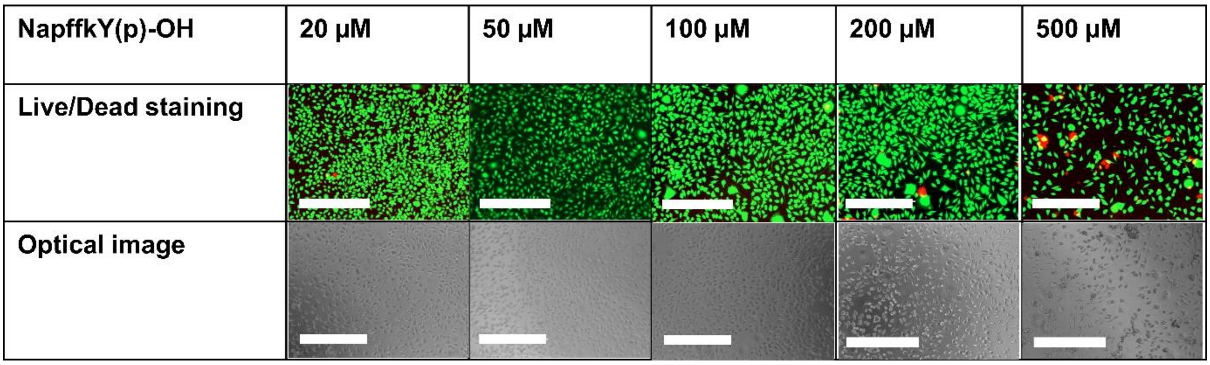

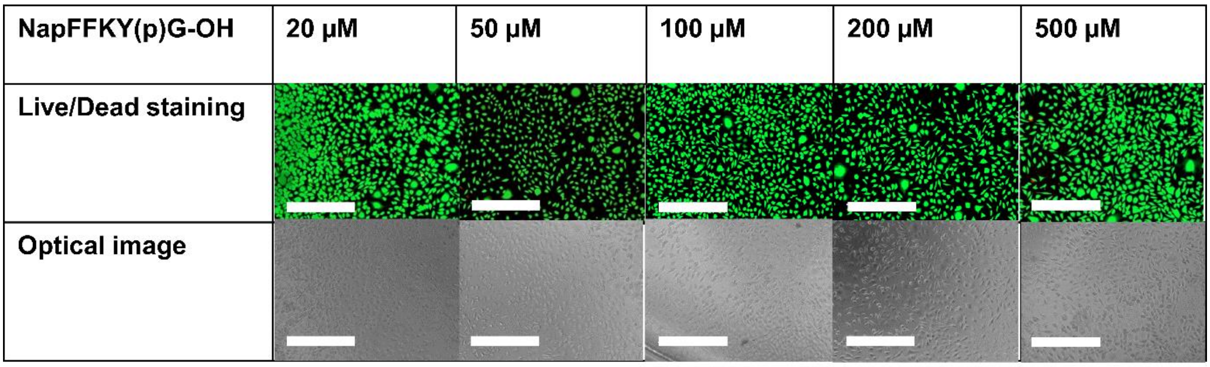

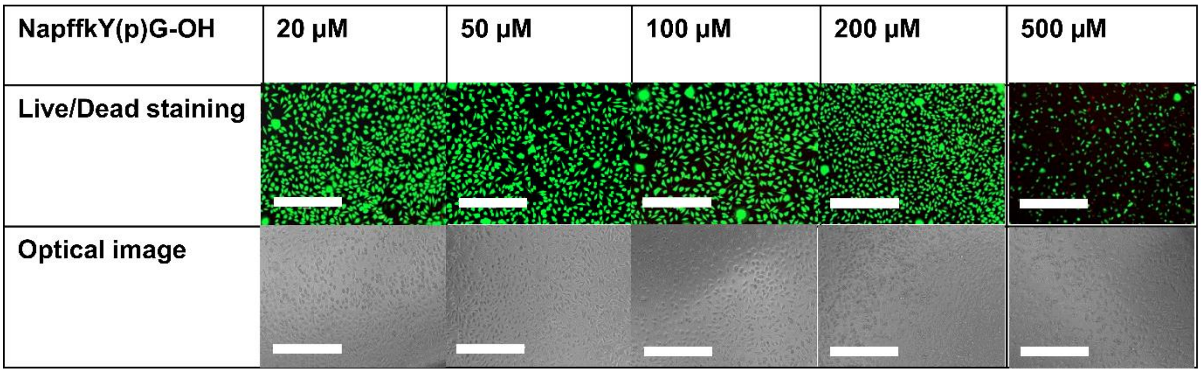

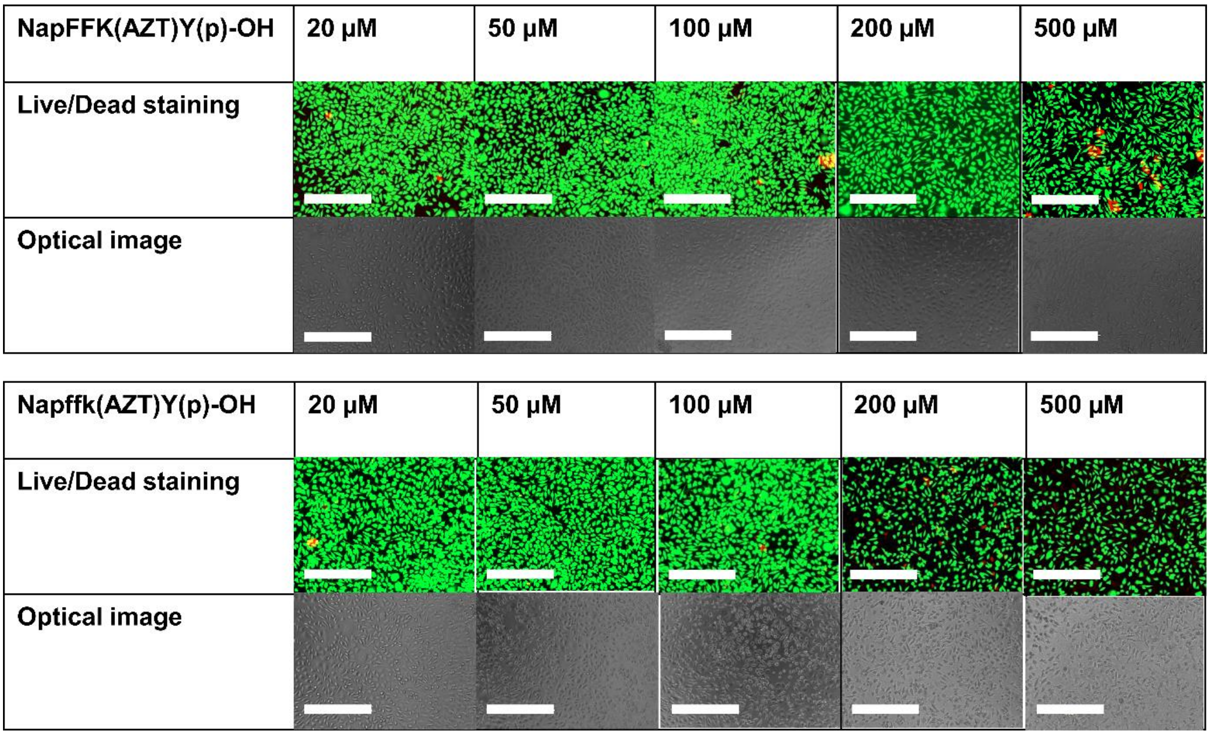

Figure S21. Cell cytotoxicity of peptides using Live/Dead® staining for 24 hours treatment with respective peptides in solubilized form (scale bar: 400 µm).

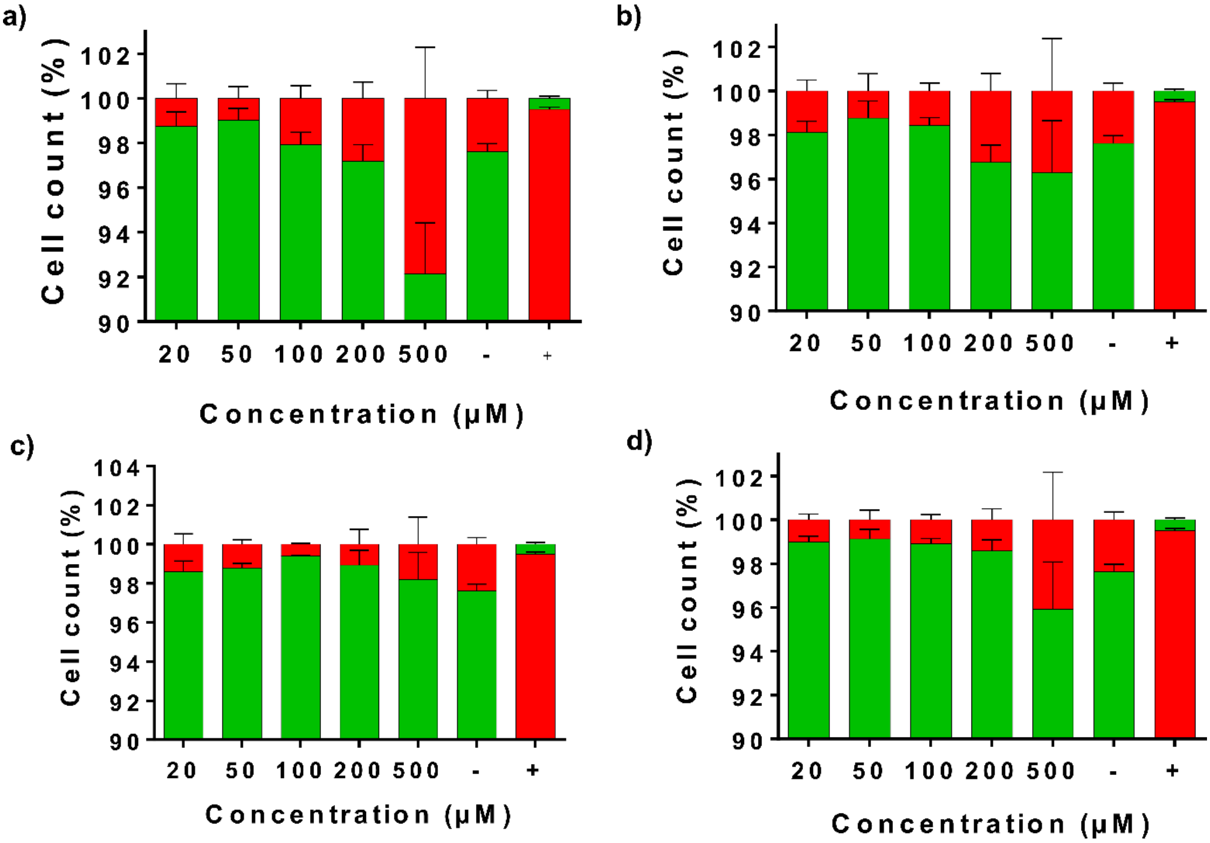

Figure S22. Live/Dead® cell counts for 24 hours treatment with respective peptides in solubilized form using ImageJ analysis, where a) NapFFKY(p)-OH, b) NapffkY(p)-

OH, c) NapFFKY(p)G-OH, d) NapffkY(p)G-OH, +: positive control (70% ethanol), -: negative control (media only), green: % live cell count, red: % dead cell count. Data were collected from 10,000 cells per well using 96 well plates.

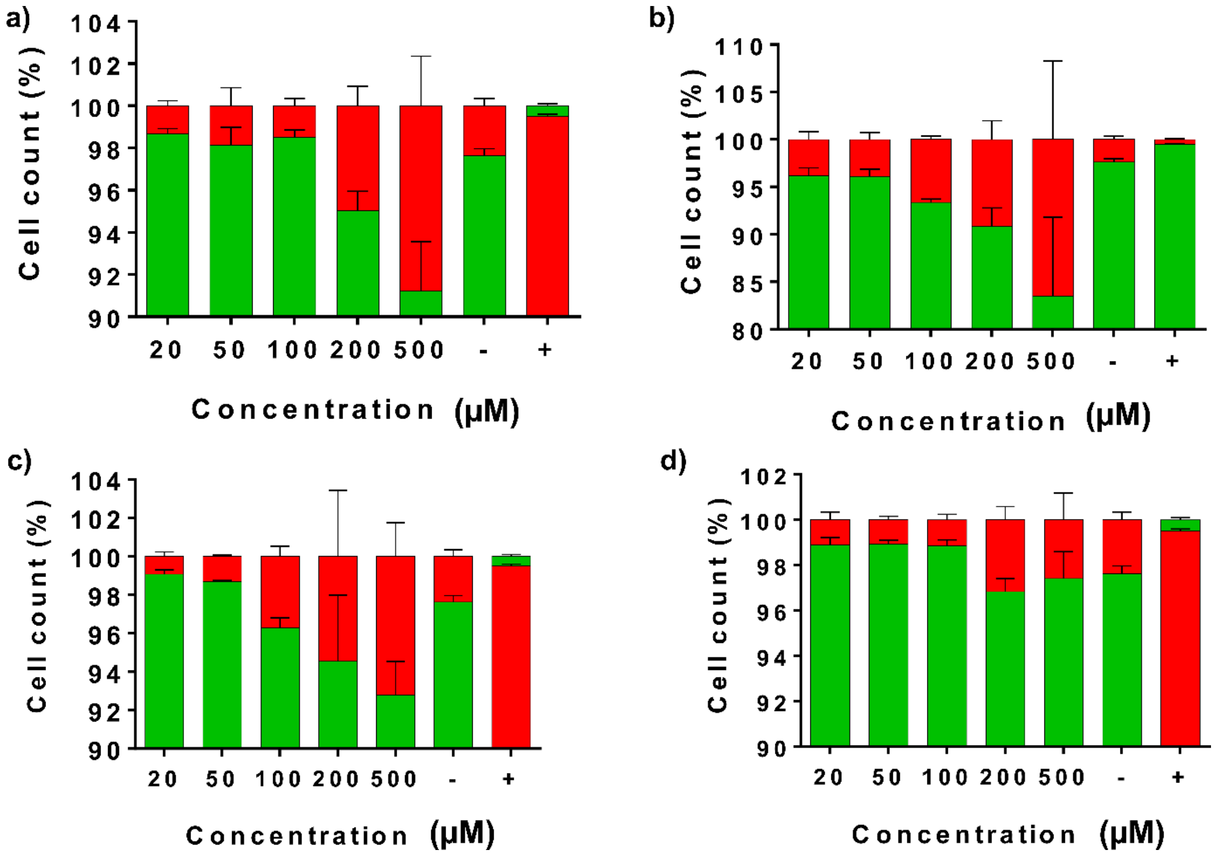

Figure S23. Live/Dead® cell counts for 24 hours treatment with respective peptides in solubilized form using ImageJ analysis, where a) NapFFK(AZT)Y(p)-OH, b) Napffk(AZT)Y(p)-OH, c) NapFFK(AZT)Y(p)G-OH and d) Napffk(AZT)Y(p)G-OH, +: positive control (70% ethanol), -: negative control (media only), green: % live cell count, red: % dead cell count. Data were collected from 10,000 cells per well using 96 well plates.

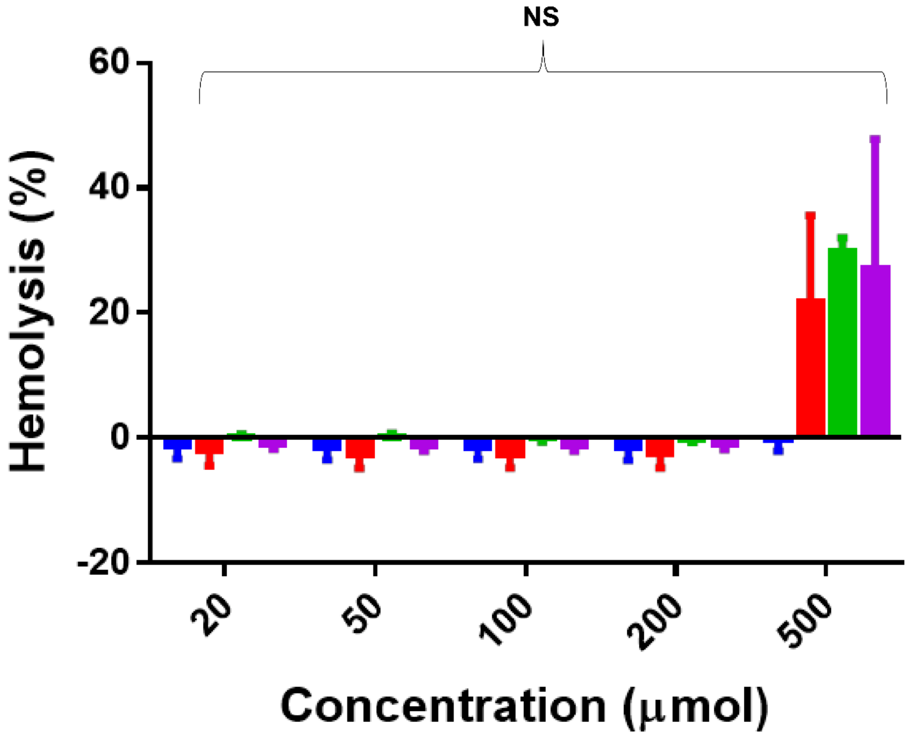

Figure S24. Cell cytotoxicity of peptides using a hemolysis assay (1 hour). Means ± SD provided. Key: a – c) NapFFKY(p)-OH: blue, NapffkY(p)-OH: red, NapFFKY(p)G-OH: green, NapffkY(p)G-OH: purple. NS: no significant ($p > 0.05$), difference between the peptides and the negative control (PBS).

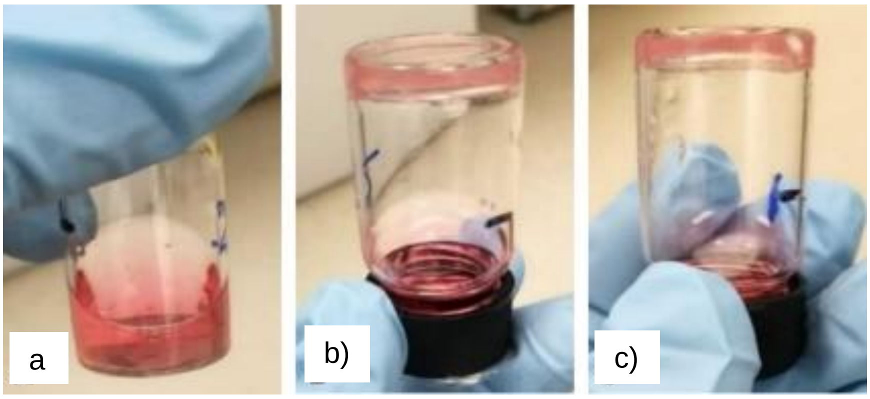

Figure S25. 2.0% w/v NapFFKYG-OH hydrogel, as used in an MTS 56 day indirect toxicity study. a) shows the addition of 2 mL MEM to the hydrogel, after overnight formation with 2 U of alkaline phosphatase at 37 °C and 5.0% CO_2. b) shows the self-supporting hydrogel holding in the bottom of the glass vial after the addition of MEM. c) shows the hydrogel at the termination of the study, day 56.

In vitro drug release

Table S5. Zidovudine release from each hydrogel, based on theoretical loading, at 72, 168 and 672 hours presented as a percentage and the number of milligrams according to the cumulative release.

Sequence	Quantity of zidovudine released at t = 72 h		Quantity of zidovudine released at t = 168 h		Quantity of zidovudine released at t = 672 h	
	**%**	**μg/mL**	**%**	**μg/mL**	**%**	**μg/mL**
NapFFKY-OH + AZT[a]	96.4	395.1	97.1	402.5	100.7	425.7
NapffkY-OH+ AZT[a]	100.6	395.3	101.0	397.5	103.8	412.9
NapFFKYG-OH + AZT[a]	91.54	384.5	97.44	409.3	101.31	425.49
NapffkYG-OH + AZT[a]	79.3	333.2	83.10	349.0	96.57	405.6
NapFFK(AZT)Y-OH	45.0	184.7	46.32	189.9	50.99	209.0
Napffk(AZT)Y-OH	51.5	211.3	53.70	220.2	62.13	254.7
NapFFK(AZT)YG-OH	33.4	140.3	35.87	150.6	40.46	169.9
Napffk(AZT)YG-OH	47.3	198.7	48.5	203.8	52.9	222.3

[a] corresponds to physical encapsulation (Fig. 5a)

Table S6. Model fitting performed using KinetDS 3.0 rev. 2010 software with the R^2 value displayed for each model

Sequence	Model					
	Zero order	First order	Ritger-Peppas	Weibull	Hixson-Crowell	Higuchi
	R^2	R^2	R^2	R^2	R^2	R^2
NapFFKY-OH + AZT[a]	0.3257	0.0416	0.9044	0.9451	0.1858	-0.4886
NapffkY-OH+ AZT[a]	0.2672	0.0413	0.9110	0.9787	0.1587	-0.6241
NapFFKYG-OH + AZT[a]	0.3312	0.0417	0.9100	0.9684	0.1771	-0.5348
NapffkYG-OH + AZT[a]	0.4285	0.0510	0.9238	0.9422	0.2147	-0.0686
NapFFK(AZT)Y-OH	0.3476	0.0336	0.8885	0.8958	0.1546	-1.9058
Napffk(AZT)Y-OH	0.4242	0.0360	0.9043	0.9135	0.1883	-0.9475
NapFFK(AZT)YG-OH	0.4594	0.0367	0.8820	0.8879	0.2037	-1.1380
Napffk(AZT)YG-OH	0.3258	0.0322	0.8957	0.9026	0.1394	-2.2059

[a] corresponds to physical encapsulation (Fig. 5a)

Table S7. The parameters fitted with the Ritger-Peppas model of drug release using KinetDS software for four-week release profiles for each sequence.

Sequence	R^2	*K*	*n*
NapFFKY-OH + AZT[a]	0.9044	4.30±0.59	0.94±0.085
NapffkY-OH+ AZT[a]	0.9110	4.29±0.57	0.94±0.082
NapFFKYG-OH + AZT[a]	0.9100	4.22±0.57	0.94±0082
NapffkYG-OH + AZT[a]	0.9238	2.62±0.49	0.96±0.07
NapFFK(AZT)Y-OH	0.8885	2.84±0.58	0.91±0.09
Napffk(AZT)Y-OH	0.9043	2.82±0.54	0.92±0.08
NapFFK(AZT)YG-OH	0.8820	2.20±0.59	0.88±0.09
Napffk(AZT)YG-OH	0.8957	2.79±0.62	0.92±0.09

***In vivo* drug release**

Sample size calculation: We have applied the following power calculation using the La Mortes Power Calculation where the following assumptions have been made:

Control: 10 days with a standard deviation of 5 days

Treated: 30 days with a standard deviation of 10 days

La Mortes power calculation:

[(Standard deviation A^2 - Standard deviation B^2) x $(Z1\text{-alpha}/2+Z1\text{-beta})^2$]/(Mean A-Mean B)^2

The $(Z1\text{-alpha}/2+Z1\text{-beta})^2$ was taken from standard tables, where alpha is the confidence level and beta is the statistical power. Therefore, for example based on 0.05 for both alpha and beta this value is 13.

So: $[(10^2 +5^2) \times 13]/(30-10)^2$

= 4.06 mice per group to attain 95% confidence levels.

We have opted for 98% alpha confidence intervals where 6 mice are needed in each treatment group.

HPLC Method: A HPLC assay method was established for determining the concentration of zidovudine in plasma using an Agilent 1260 Series system (UV detector, quaternary pump, auto sampling injector and Agilent Chemstation). An ODS-3 analytical column (250 mm × 4.6 mm internal diameter, 5 µm packing; InertClone™, Phenomenex, USA) was also utilized The UV detector was operated at 266 nm at room temperature and a flow rate of 1.25 mL/min. The mobile phase was acetonitrile – 0.1% ortho-phosphoric acid in water (15 – 85 v/v) was employed as the eluent.[5]

Preparation of zidovudine standards and zidovudine extraction from plasma: Zidovudine stock solution (1 mg/mL) was prepared in Milli-Q water. This stock solution was diluted in Milli-Q water to the final concentrations required for the standard calibration curve, in the range 500 to 50000 ng/mL. 10 µL aliquots of each standard were added to 90 µL of rat plasma in 200 µL microtubes. The mixture was vortexed at 1400 rpm for 30 min at 25 °C using an Eppendorf Theromomixer comfort

and a final calibration standard concentration range of 50 to 5000 ng/mL was attained (Figure S22). Then, 50 μL of perchloric acid was added to each microtube to precipitate the plasma proteins. The mixture was vortexed at 1400 rpm for 20 min at 25°C. Finally, the samples were centrifuged at 13,400 rpm for 20 min at room temperature (Eppendorf minispin). The supernatants were collected into Supelco HPLC glass inserts inside HPLC vials. Nine calibrations were constructed over 3 days to validate the analytical method and ensure its reproducibility**.**

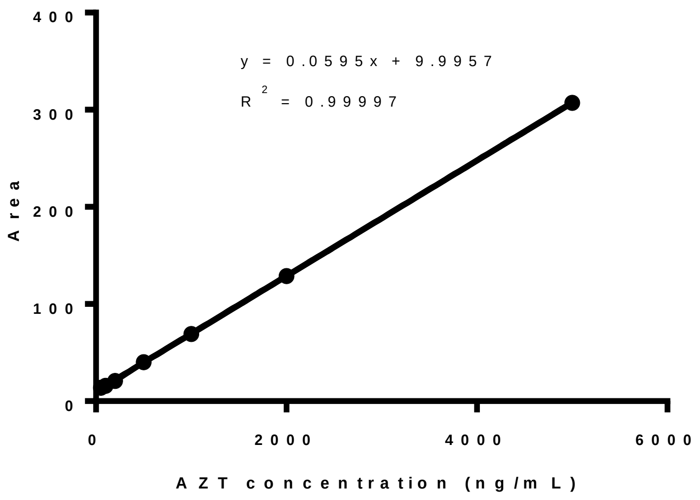

Figure S26. Calibration curve developed for zidovudine extracted from Sprague Dawley rat plasma (n = 9).

***In vivo* plasma preparation and zidovudine extraction:** Rat plasma samples were thawed to room temperature and 100 μL was transferred to microtubes. 50 μL of Perchloric acid was added to each microtube and vortexed at 1400 rpm for 30 min at 25 °C to precipitate the plasma proteins. Then, the samples were centrifuged at 13400 rpm for 20 min, and the supernatant collected in HPLC glass inserts inside HPLC vials. The pharmacokinetic parameters, area under the plasma concentration-time curve from time zero to the time of last measurable concentration ($AUC_{0\text{-}t}$), area under the plasma concentration-time curve from time zero to infinity ($AUC_{0\text{-}\infty}$), plasma half-life ($t_{1/2}$), total body clearance (CL_{tot}), apparent volume of distribution (V_d), and mean residence time (MRT) were computed by a noncompartmental model applying the program PKSolver 2.0.[6]

Table S8. Comparison of the *in vivo* pharmacokinetic parameters of zidovudine after intravenous administration of drug and subcutaneous administration of chemically conjugated peptide Napffk(AZT)YG-OH to female Sprague Dawley rats. Means ± SEMs, displayed for each group (n = 6).

Parameter	Unit	Zidovudine intravenous ± SEM	Napffk(AZT)YG-OH subcutaneous ± SEM
$t_{1/2}$	h	1.7±0.53	674.48±99.61
T_{max}	h	0.58 ± 0.08	264 ± 108.75
C_{max}	µg/mL	6.48 ± 1.67	0.71 ± 0.15
$AUC_{0\text{-}t}$	µg/mL/h	15.48 ± 2.60	207.60 ± 64.36
$AUC_{0\text{-}\infty,obs}$	µg/mL/h	16.55 ± 2.73	280.20 ± 58.86
$MRT_{0\text{-}\infty,obs}$	h	1.58 ± 0.50	1089.5 ± 188.19

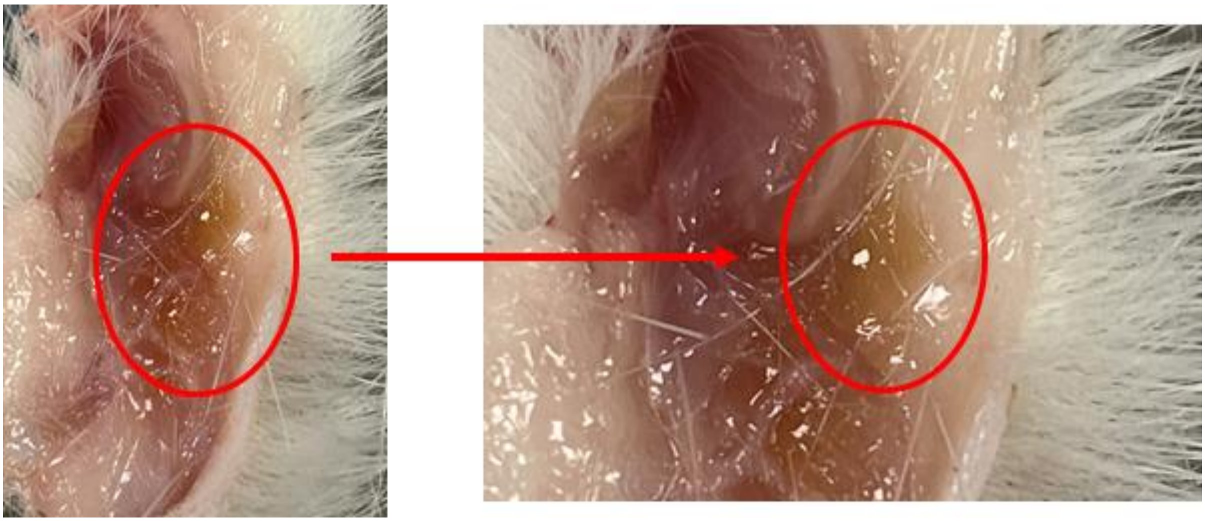

Figure S27. Image of the remaining Napffk(AZT)YG-OH hydrogel (red circle) at the injection site at the termination of the 35 day study in Sprague Dawley rats.

References

[1] V. K. Sarin, S. B. Kent, J. P. Tam, R. B. Merrifield, *Anal Biochem* **1981**, *117*, 147.

[2] P. Sieber, *Tetrahedron Letters* **1987**, *28*, 6147.

[3] M. Quibell, in *Fmoc solid phase peptide synthesis: A practical approach.* (Ed: W. Chan, White, P.), Oxford University Press., Oxford, United Kingdom. **1999**.

[4] J. Li, X. Li, Y. Kuang, Y. Gao, X. Du, J. Shi, B. Xu, *Advanced Healthcare Materials* **2013**, *2*, 1586.

[5] S. Wannachaiyasit, P. Chanvorachote, U. Nimmannit, *AAPS PharmSciTech* **2008**, *9*, 840.

[6] Y. Zhang, M. Huo, J. Zhou, S. Xie, *Comput Methods Programs Biomed* **2010**, *99*, 306.
